# Mechanisms of action and resistance in histone methylation-targeted therapy

**DOI:** 10.1038/s41586-024-07103-x

**Published:** 2024-02-21

**Authors:** Makoto Yamagishi, Yuta Kuze, Seiichiro Kobayashi, Makoto Nakashima, Satoko Morishima, Toyotaka Kawamata, Junya Makiyama, Kako Suzuki, Masahide Seki, Kazumi Abe, Kiyomi Imamura, Eri Watanabe, Kazumi Tsuchiya, Isao Yasumatsu, Gensuke Takayama, Yoshiyuki Hizukuri, Kazumi Ito, Yukihiro Taira, Yasuhito Nannya, Arinobu Tojo, Toshiki Watanabe, Shinji Tsutsumi, Yutaka Suzuki, Kaoru Uchimaru

**Affiliations:** 1 Laboratory of Viral Oncology and Genomics, Department of Computational Biology and Medical Sciences, Graduate School of Frontier Sciences, The University of Tokyo, Tokyo, Japan; 2 Laboratory of Tumor Cell Biology, Department of Computational Biology and Medical Sciences, Graduate School of Frontier Sciences, The University of Tokyo, Tokyo, Japan; 3 Laboratory of Systems Genomics, Department of Computational Biology and Medical Sciences, Graduate School of Frontier Sciences, The University of Tokyo, Tokyo, Japan; 4 Division of Hematopoietic Disease Control, The Institute of Medical Science, The University of Tokyo, Tokyo, Japan; 5 Department of Hematology, Kanto Rosai Hospital, Kanagawa, Japan; 6 Division of Endocrinology, Diabetes and Metabolism, Hematology and Rheumatology, Second Department of Internal Medicine, Graduate School of Medicine, University of the Ryukyus, Okinawa, Japan; 7 Department of Hematology/Oncology, IMSUT Hospital, The Institute of Medical Science, The University of Tokyo, Tokyo, Japan; 8 Department of Hematology, Sasebo City General Hospital, Nagasaki, Japan; 9 IMSUT Clinical Flow Cytometry Laboratory, The Institute of Medical Science, The University of Tokyo, Tokyo, Japan; 10 Organic and Biomolecular Chemistry Department, Daiichi Sankyo RD Novare, Tokyo, Japan; 11 Translational Science I, Daiichi Sankyo, Tokyo, Japan; 12 Tokyo Medical and Dental University, Tokyo, Japan; 13 Department of Practical Management of Medical Information, Graduate School of Medicine, St Marianna University, Kanagawa, Japan

**Keywords:** Haematological cancer, Epigenetics

## Abstract

Epigenomes enable the rectification of disordered cancer gene expression, thereby providing new targets for pharmacological interventions. The clinical utility of targeting histone H3 lysine trimethylation (H3K27me3) as an epigenetic hallmark has been demonstrated^1–7^. However, in actual therapeutic settings, the mechanism by which H3K27me3-targeting therapies exert their effects and the response of tumour cells remain unclear. Here we show the potency and mechanisms of action and resistance of the EZH1–EZH2 dual inhibitor valemetostat in clinical trials of patients with adult T cell leukaemia/lymphoma. Administration of valemetostat reduced tumour size and demonstrated durable clinical response in aggressive lymphomas with multiple genetic mutations. Integrative single-cell analyses showed that valemetostat abolishes the highly condensed chromatin structure formed by the plastic H3K27me3 and neutralizes multiple gene loci, including tumour suppressor genes. Nevertheless, subsequent long-term treatment encounters the emergence of resistant clones with reconstructed aggregate chromatin that closely resemble the pre-dose state. Acquired mutations at the PRC2–compound interface result in the propagation of clones with increased H3K27me3 expression. In patients free of PRC2 mutations, *TET2* mutation or elevated *DNMT3A* expression causes similar chromatin recondensation through de novo DNA methylation in the H3K27me3-associated regions. We identified subpopulations with distinct metabolic and gene translation characteristics implicated in primary susceptibility until the acquisition of the heritable (epi)mutations. Targeting epigenetic drivers and chromatin homeostasis may provide opportunities for further sustained epigenetic cancer therapies.

## Abstract (web summary)

The mechanisms of action and resistance of valemetostat, an EZH1–EZH2 dual inhibitor, in patients with adult T cell leukaemia/lymphoma who initially responded but later showed disease progression are explored.

## Main

H3K27me3 is a cancer hallmark that accumulates around the promoter regions of genes that should be properly expressed. Consequently, the chromatin structure becomes condensed and the genes essential for cell identity and appropriate functions are suppressed^1^. Excessive H3K27me3 is among the principal epigenetic drivers in cancers^2,3^.

The H3K27me3 enzymes EZH1 and EZH2 are compensatory factors that enable stable regulation of methylation patterns^8–10^. EZH2 is a histone modifier that is frequently detected to be abnormal in cancers and alters the entire epigenome by increasing H3K27me3 levels. Targeting EZH2 provides a therapeutic benefit in B cell lymphomas and certain solid tumours with vulnerabilities^4,5^. Valemetostat is a first-in-class EZH1–EZH2 (EZH1/2) dual inhibitor that can block the complementary effects of EZH1/2 (refs. ^10,11^). It is expected to be more efficient than EZH2-selective inhibitors in eliminating H3K27me3 and is highly effective against lymphomas. Clinical trials of valemetostat have shown its sustained safety and efficacy against HTLV-1-associated aggressive adult T cell leukaemia/lymphoma (ATL) and other lymphomas^6,7^.

Next-generation epigenetic therapies targeting H3K27me3 are promising and rapidly developing, and clinical trials are being conducted for various cancer types. However, it remains unclear how the systemic H3K27me3-based therapies affect the tumour epigenome in patients to elicit the clinical efficacy. Moreover, clinical recurrences after a long period have also been observed in the trials; however, the underlying mechanisms still remain unclear.

## Clinical benefits of EZH1/2 inhibition

We conducted timelapse analyses of the patients enrolled for the valemetostat clinical trials. ATL, a rare type of T cell lymphoma with poor prognosis^12,13^ particularly renowned for its increased H3K27me3 levels^14,15^, was selected as the target for the first-in-human clinical trial of valemetostat^6^. In total, ten participants among the enrolled patients in two clinical trials (patients 1–3 (Pt1–3) from phase I as the discovery cohort and Pt4–10 from phase II as the validation cohort) were subjected to intensive molecular monitoring.

Valemetostat was administered orally once daily (200 mg daily) until a sign of disease progression could be observed (Supplementary Table 1). The patient responses to the therapy and tumour dynamics were observed after administration of valemetostat. In all cases, lesions were detected in the peripheral blood, allowing us to collect and analyse peripheral blood mononuclear cells (PBMCs) to directly assess the clinical efficacy and tumour characteristics using less-invasive tests. We applied the HTLV-1 provirus integrated within the tumour genome to improve tumour cell identification accuracy.

**Fig. 1:**
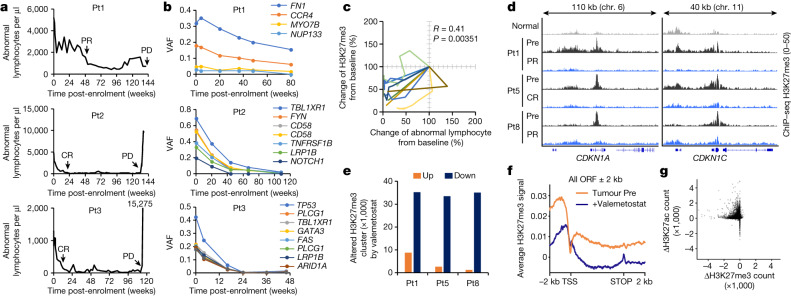
Antitumour effect of valemetostat. **a**, Changes in abnormal lymphocytes of three cases in a first-in-human valemetostat phase I study. Valemetostat was administered orally once daily (200 mg daily) until a sign of disease progression was observed. Clinical diagnoses (partial response (PR), complete response (CR) and progressive disease (PD)) are annotated. **b**, Changes in variant allele frequency (VAF) of major somatic mutations from the initiation of treatment identified by targeted deep sequencing of peripheral blood. **c**, Correlation between changes of abnormal lymphocyte and H3K27me3 from baseline (%) in nine patients. **d**, Representative tracks (*CDKN1A* and *CDKN1C* loci) for H3K27me3 in Pt1, Pt5 and Pt8 before and after valemetostat treatment. Chr., chromosome. **e**, The number of altered H3K27me3 clusters after treatment in three cases detected by ChIP–seq. **f**, Average ChIP–seq signal profiles for H3K27me3 in tumour baseline (Pre) and after treatment (48 weeks) around the TSS and across the gene body. ORF, open reading frame. **g**, Treatment-associated changes of H3K27me3 (*x* axis) and H3K27ac (*y* axis) at ChIP–seq-merged all peaks. Statistics and reproducibility are described in the Methods. Source Data

**Extended Data Fig. 1:**
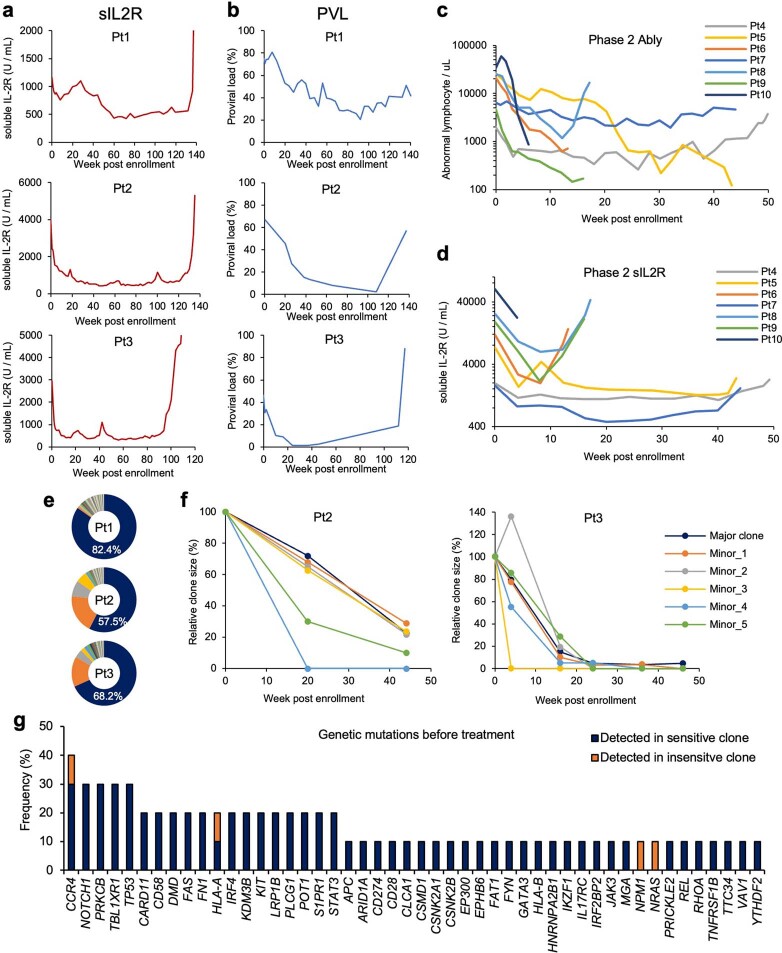
Clinical efficacy and genomic profiling in valemetostat trials. **a**, **b**, Changes over time in soluble IL2 receptor (sIL-2R) (**a**) and proviral loads (PVL) (**b**) of three cases in valemetostat phase 1 study. **c**, **d**, Changes in abnormal lymphocytes (**c**) and sIL-2R (**d**) of 7 cases in valemetostat phase 2 study. **e**, Pie charts show baseline clonalities in three patients. The clonality of HTLV-1-infected cells before valemetostat treatment was calculated as the population size of each clone by counting the extracted reads at host-provirus junction sites using high-throughput sequencing based mapping of proviral integration sites. **f**, Changes over time in the size of top 5 clones in Pt2 (left) and Pt3 (right) after treatment with valemetostat. **g**, Frequency of somatic mutations detected by targeted genome sequencing in valemetostat phase 1 and 2 studies (*n* = 10, biologically independent samples). Source Data

The number of abnormal lymphocytes drastically decreased after 1 week of treatment (Fig. 1a). In parallel, we also observed a significant reduction in soluble IL-2 receptor-α (sIL-2Rα) and HTLV-1-infected cell counts, denoted as proviral load (Extended Data Fig. 1a,b). Pt1 showed a partial response. Pt2 and Pt3 showed complete response at 20 and 12 weeks, respectively. All patients were able to stay on single-agent valemetostat for more than 2 years with acceptable safety and durable response. Likewise, rapid responses were observed and clinically diagnosed as partial response or complete response in the validation cohort (*n* = 7) (Extended Data Fig. 1c,d).

All PBMC samples (*n* = 104) were collected from the patients immediately before (Pre) and during treatment, as well as during the progressive disease stage (Supplementary Table 1). Whole-genome sequencing of the Pre-samples revealed a significant number of somatic mutations as single-base mutations (3.9 mutations per megabyte per sample on average) or copy number variations typical to ATL^16^ (Supplementary Tables 2 and 3). Pt3 was characterized as a poor prognostic type harbouring a homozygous *TP53* mutation and *PDL1* (also known as *CD274*) structural variation^17^. Targeted deep sequencing revealed that the variant allele frequency of the major clones decreased in parallel with decreasing abnormal lymphocytes and proviral load dynamics (Fig. 1b). We further used HTLV-1 provirus frequency to quantify the size of multiple clones^18^. Major clones and unexpanded subclones were significantly depleted by valemetostat with similar dynamics (Extended Data Fig. 1e,f). Targeted genome profiling showed that the mutation pattern of the responders was similar to that of the general cohort^16^. Malignant clones with poor prognostic variations, including *PRKCB*, *TP53*, *IRF4* and *PDL1* (ref. ^19^), were diminished after treatment (Extended Data Fig. 1g and Supplementary Table 4).

**Extended Data Fig. 2:**
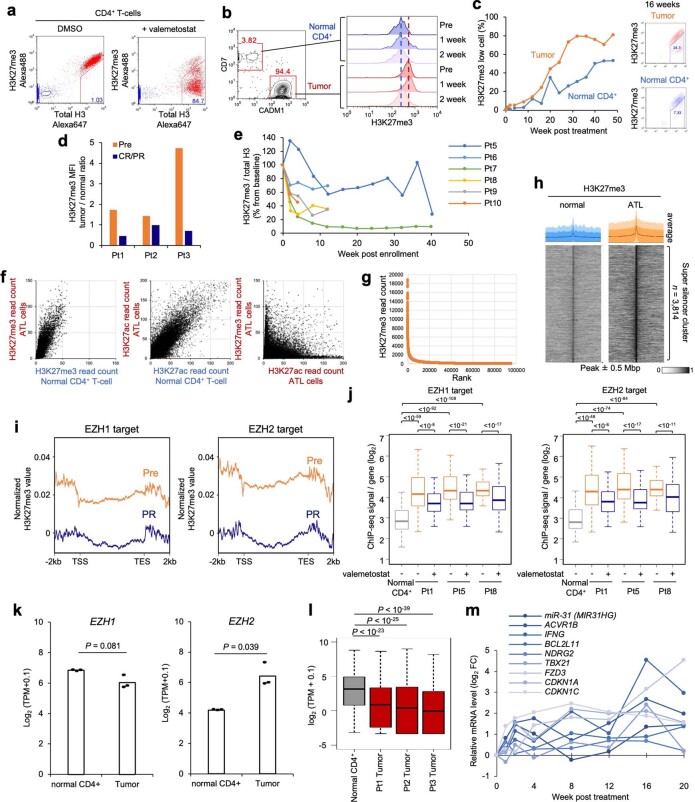
H3K27me3 reduction by valemetostat. **a**, Establishment of H3K27me3-flow system. Intracellular H3K27me3 / total histone H3 were stained with specific antibodies in PHA-activated PBMC. Treatment with 100 nM valemetostat for 7 days significantly reduced the H3K27me3 level. Red cells, antibody + ; blue cells, isotype control. **b**, H3K27me3 levels in tumor (CD4^+^/CADM1^+^/CD7^−^) and normal (CD4^+^/CADM1^−^/CD7^+^) cell populations at 0, 1, and 2 weeks post valemetostat treatment in Pt1. Dashed lines indicate baselines. **c**, Changes over time in population size of low H3K27me3 cells (%) in Pt1. **d**, Tumor H3K27me3 levels (mean fluorescence intensity, MFI) normalized by the level of normal CD4^+^ T-cells at pre-treatment (Pre) and at the time of clinical response (PR/CR). **e**, Changes of tumor H3K27me3 level (% from baseline) in valemetostat phase 2 study. **f**, ChIP-seq signal values of H3K27me3 (left panel) and H3K27ac (middle panel) at all gene promoters (32,747 genes) in Pt1 tumor cells (y-axis) versus normal CD4^+^ T-cells (x-axis). The distribution of the H3K27me3 and H3K27ac peaks was mutually exclusive (right panel). **g**, **h**, H3K27me3 enriched super-silencer clusters in ATL cells. Rank ordering of H3K27me3 ChIP-seq signals identified the super-silencer clusters (**g**). Heatmaps and average profiles show ChIP-seq signals centered on peaks, ranked by mean H3K27me3 level, in normal CD4^+^ T-cells and ATL cells (**h**). **i**, **j**, Average ChIP-seq TSS-TES plot (**i**) and H3K27me3 log_10_ signals at promoter regions (**j**) bound by EZH1 (*n* = 347 genes) and EZH2 (*n* = 269 genes) in Pt1, Pt5, and Pt8. EZH1 and EZH2 ChIP data resources^10^ were re-analyzed. **k**, **l**, Baseline expression [Log_2_(TPM + 0.1)] of *EZH1* and *EZH2* mRNA (**k**) and H3K27me3 target genes (*n* = 630) (**l**) in tumor cells from three patients and normal CD4^+^ T cells from healthy donors. Tumor-specific RNA was collected by CD4^+^/CADM1^+^/CD7^−^ cell sorting and analyzed by RNA-seq. **m**, Relative expression changes (log_2_ fold-change) of the representative H3K27me3 target genes post valemetostat treatment in Pt1. The middle lines within box plots correspond to the medians; lower and upper hinges correspond to the first and third quartiles. The upper whisker extends from the hinge to the largest value no further than 1.5 * IQR. The lower whisker extends from the hinge to the smallest value at most 1.5 * IQR. Statistics and reproducibility are described in Methods. Source Data

We used a flow cytometry-based H3K27me3 assay^20^ to quantify the methylation changes in the ATL cells. The observed tumour H3K27me3 level was generally high in the Pre-samples, but the valemetostat treatment reduced it to a normal level (Extended Data Fig. 2a–c). Rapid reduction of H3K27me3 levels was observed in the tested patients (*n* = 9), and a statistical correlation between H3K27me3 loss and clinical benefit was observed (Fig. 1c and Extended Data Fig. 2d,e).

We next performed chromatin immunoprecipitation followed by sequencing (ChIP–seq) to further assess how valemetostat affects the tumour epigenome. Clinical specimens from Pt1, Pt5 and Pt8 were available, and tumour cells before treatment and at clinical response were sorting-enriched (more than 95%) and analysed for tumour-specific H3K27me3. The pre-treatment tumour cells showed an overall increasing trend in H3K27me3 compared with normal T cells (Extended Data Fig. 2f–h). The H3K27 acetylation (H3K27ac) pattern inversely correlated with H3K27me3. In particular, the H3K27me3-mediated super-silencer cluster^21^ was observed spanning over 1 Mb around several regions.

H3K27me3 profiling before the treatment showed that H3K27me3 clusters averaging more than 10 kb (0.199–288 kb) in length were established mainly around the transcription start site (TSS). Valemetostat treatment significantly reduced H3K27me3 levels in tumour suppressor genes (TSGs), showing a genome-wide reduction of H3K27me3 peaks (more than 35,000 peaks) in all cases (Fig. 1d,e). Focusing on the gene loci, an overall decrease was observed around the TSS and across the gene body (Fig. 1f,g). Moreover, H3K27ac levels increased in the regions where H3K27me3 levels were reduced. Both EZH1 and EZH2 targets were alleviated similarly (Extended Data Fig. 2i,j). Tumour-specific bulk RNA sequencing (RNA-seq) confirmed that typical target genes^10^ were restored immediately after the treatment (Extended Data Fig. 2k–m). These results indicate that valemetostat sufficiently restored the epigenome of the tumour cells close to the healthy state, leading to clinical improvements.

## Chromatin reprogramming by valemetostat

**Fig. 2:**
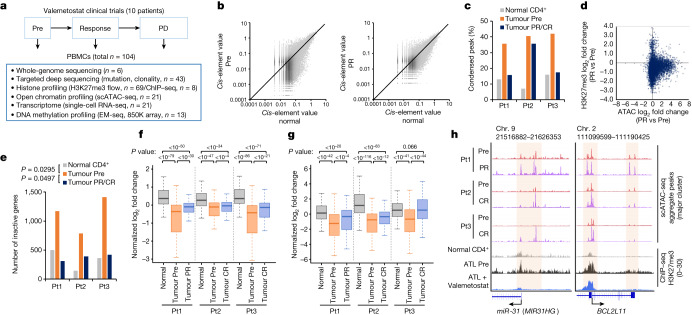
Chromatin decondensation by valemetostat. **a**, The workflow illustrates the collection and processing of fresh peripheral blood samples from a clinical trial and the following multilayered experimental platform. ChIP–seq, chromatin immunoprecipitation with sequencing. EM-seq, enzymatic methyl sequencing. **b**, All ATAC peak values (total 69,544 peaks) of tumour cells (*y* axis) at pre-treatment (left) and after treatment (48 weeks; right) versus normal CD4^+^ T cells (*x* axis) in a representative case (Pt1). **c**, Proportion of chromatin-condensed peaks (*cis*-element value < 0.01) from scATAC-seq data in three patients. **d**, Scatter plot of log_2_ fold changes of ATAC (*x* axis) and H3K27me3 (*y* axis) at partial response (48 weeks) for all gene promoter regions in Pt1. **e**, Numbers of chromatin inactive genes (promoter sum < 0.01) in three patients. **f**,**g**, Box plots summarize normalized log_2_ fold changes of scATAC-seq promoter activities (**f**) and scRNA-seq gene expression (**g**) at H3K27me3 target genes (563 genes) in three patients. Statistical significance is provided only for main combinations. The middle line within the box plots corresponds to the median; the lower and upper hinges correspond to the first and third quartiles; the upper whisker extends from the hinge to the largest value no further than 1.5 times the interquartile range (IQR); and the lower whisker extends from the hinge to the smallest value at most 1.5 times the IQR. **h**, Aggregate scATAC tracks and H3K27me3 distribution before and after valemetostat treatment at the representative H3K27me3 target loci (*miR-31* and *BCL2L11*) in three patients. Highlighted regions show chromatin decondensation by valemetostat. Statistics and reproducibility are described in the Methods. Source Data

**Extended Data Fig. 3:**
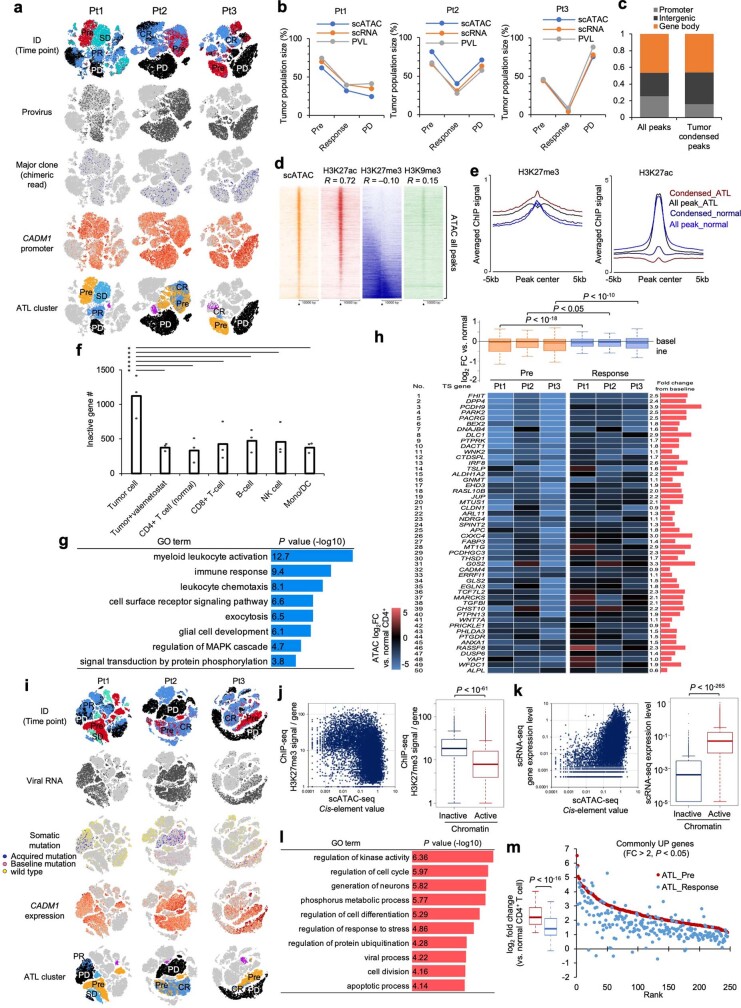
Characterization of tumor chromatin architecture and transcriptome. **a**, Summary of scATAC-seq clustering (*n* = 10 from 3 patients). Cells were colored according to sample timepoint, provirus, host-virus chimeric reads, *CADM1* promoter activity, and assigned major tumor clusters. **b**, Tumor population size (%) before and after valemetostat treatment inferred by scATAC-seq, scRNA-seq, and PVL. **c**, Features of tumor-associated chromatin condensed peaks. **d**, Heatmaps of all ATAC 69,545 peaks and associated ChIP values of H3K27me3, H3K9me3, H3K27ac in Pt1 tumor cells. Each correlation coefficient (*R*) between histone mark and ATAC peak are provided. **e**, Average ChIP-seq signal profiles for H3K27me3 (left) and H3K27ac (right) at chromatin condensed peaks in tumor cells and normal CD4^+^ T-cells around peak center. **f**, Numbers of chromatin-inactivated genes (Promoter sum <0.01) in peripheral blood cell lineage from scATAC-seq data (*n* = 3 biologically independent samples, * *P* < 0.05). **g**, Bar graph shows enriched gene ontology terms with one-sided Fisher’s exact *P* values (−Log_10_) for commonly inactivated genes (Promoter sum <0.01 in three patients). **h**, Reactivation of TSG by valemetostat treatment. Boxplot (top panel) shows normalized log_2_ fold changes of scATAC-seq promoter activities by valemetostat treatment in TSG (*n* = 716 genes). Heatmap (bottom panel) show scATAC-seq promoter activities (log_2_ fold-change) of top 50 TSG in major clones of three patients before and after valemetostat treatment. **i**, Summary of scRNA-seq clustering (*n* = 10 from 3 patients). Cells were colored according to sample timepoint, viral RNA, mutated RNA, *CADM1* expression, and assigned major tumor clusters. **j**, Data integration of scATAC-seq and H3K27me3 ChIP-seq in Pt1. Scatter plot (left) shows scATAC-seq promoter activity (x-axis) and H3K27me3 ChIP-seq signals (y-axis) for all gene promoter regions. Boxplot (right) summarizes H3K27me3 signals at active (Promoter sum > 0.01, 11,310 gene) and inactive (Promoter sum <0.01, 1,032 gene) promoter regions. **k**, Data integration of scATAC-seq and scRNA-seq in Pt1. Scatter plot (left) shows scATAC-seq promoter activity (x-axis) and scRNA-seq expression level (y-axis) for all genes. Boxplot (right) summarizes gene expression levels at active (Promoter sum > 0.01) and inactive (Promoter sum <0.01) genes. **l**, Bar graph shows enriched gene ontology terms with one-sided Fisher’s exact *P* values (−log_10_) for commonly overexpressed genes in ATL cells (Gene expression log_2_ fold-change > 2 versus normal T-cells in three patients, 246 genes, *P* < 0.05). **m**, Average (*n* = 3 biologically independent samples) of normalized log_2_ fold changes for commonly overexpressed genes before and after valemetostat treatment. The middle lines within box plots correspond to the medians; lower and upper hinges correspond to the first and third quartiles. The upper whisker extends from the hinge to the largest value no further than 1.5 * IQR. The lower whisker extends from the hinge to the smallest value at most 1.5 * IQR. Statistics and reproducibility are described in Methods. Source Data

We performed single-cell assay for transposase-accessible chromatin with sequencing (scATAC-seq) to evaluate the influence of valemetostat on the chromatin structure and gene regulation. We collected live PBMC samples (total *n* = 10 from phase I) at Pre, at the time of clinical response (partial response or complete response) and at progressive disease and sequenced 85,480 cells (Fig. 2a). In general, an average of 86,351 peaks per sample could be detected. These peaks included those at the promoter regions of the marker genes in the ATL cells (for example, *CADM1*), confirming that those cells were indeed ATL cells. The HTLV-1 provirus reads were also useful in detecting minimal residual disease, thus allowing the identification and analysis of even those tumour cells that were reduced to less than 5% as a result of the treatment (Extended Data Fig. 3a,b). Provirus and host genome chimeric reads^18^ gave helpful information for identifying the clonal origin of the cells.

Compared with normal CD4^+^ T cells, the propagated tumour cells before the treatment showed an aggregated chromatin structure across the entire genomic regions in all cases (an average of 39.4% of the detected ATAC peaks, *P* < 0.05) (Fig. 2b,c and Extended Data Fig. 3c). Referring to the ChIP–seq data of the same case, the ATAC peaks were negatively correlated with the H3K27me3 levels. By contrast, the H3K27me3 mark accumulated in the tumour-associated condensed chromatin regions (Extended Data Fig. 3d,e). After valemetostat treatment, the condensed regions decreased in all cases. Pt1 and Pt3, whose H3K27me3 levels were markedly diminished by valemetostat, showed relaxed chromatin structures comparable with normal cells after the treatment (Fig. 2b,c). Focusing on the promoter regions, a substantial number of gene promoters were inactivated in the tumour cells at Pre (1,121 genes on average). The reduction in H3K27me3 levels by valemetostat was correlated with chromatin relaxation, reducing the number of inactivated genes to the level of normal cells in all cases (*P* < 0.05) (Fig. 2d,e and Extended Data Fig. 3f). The inactivated genes included several genes associated with T cell function and immune response. Certain TSGs^22^ were inactivated at Pre but were restored by valemetostat (Extended Data Fig. 3g,h).

To further investigate the significance of chromatin structural changes on gene expression, we performed single-cell RNA-seq (scRNA-seq) for the PBMC samples (*n* = 10, 98,358 cells) (Fig. 2a). Tumour cell clusters could be identified in the *t*-distributed stochastic neighbour embedding (*t*-SNE) planar by gene mutations, viral reads and marker gene expressions (Extended Data Fig. 3i). Integration with the corresponding scATAC-seq data showed a positive correlation between gene expression and promoter activity (*R* = 0.608). Expression of the loci with condensed chromatin promoters (*cis*-element value < 0.01) was inactivated with H3K27me3 (Extended Data Fig. 3j,k). The integrated data demonstrated that valemetostat relaxed the chromatin structure and induced expression at the loci where H3K27me3 had been accumulated (Fig. 2f,g). The chromatin accessibility of the representative H3K27me3 target genes (*miR-31*, *BCL2L11*, among others)^10,14^ was increased by valemetostat (Fig. 2h).

As abnormal H3K27me3 enrichment was common at the loci of transcription factors and microRNAs, changes in H3K27me3 had various secondary effects^2,10,23^. A total of 246 genes (fold change > 2, *P* < 0.05) were commonly upregulated in ATL cells before treatment, including genes associated with cell growth and apoptosis regulation. By rectifying the epigenomic regulations upon valemetostat treatment, 89.4% of these abnormal genes were repressed (Extended Data Fig. 3l,m).

**Extended Data Fig. 4:**
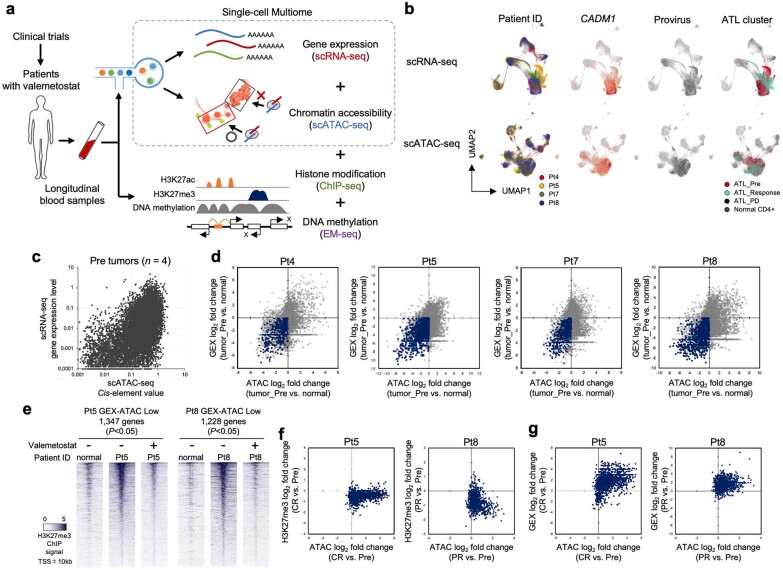
Integrated single-cell analyses for phase 2 validation cohort. **a**, Workflow illustrates single-cell analyses platform for phase 2 study. Chromatin structure (ATAC) and gene expression (GEX) data from total 109,830 cells were connected at the single-cell level by the 10x Genomics scMultiome platform. Epigenetic profiling data (ChIP-seq and EM-seq) of corresponding samples are integrated. **b**, Summary of scMultiome clustering for 11 libraries from 4 patients. Cells were colored according to patient ID, *CADM1* expression, provirus read, and assigned major tumor clusters. **c**, Scatter plot shows scATAC-seq promoter activity (x-axis) and scRNA-seq expression level (y-axis) for all genes in the four tumors before treatment. **d**, Scatter plots show Log_2_ fold changes of ATAC (x-axis) and GEX (y-axis) for all genes in Pre tumors vs. normal cells. Gene clusters silenced by the chromatin aggregation (*P* < 0.05) are indicated by dark blue. **e**, Heatmaps depict sorted H3K27me3 peaks centered on TSS (20-kb windows) at chromatin inactivated gene clusters in Pt5 and Pt8 before and after valemetostat treatment. **f**, Scatter plots show Log_2_ fold changes from baseline of ATAC (x-axis) and H3K27me3 (y-axis) for chromatin inactivated gene clusters at clinical response in Pt5 and Pt8. **g**, Scatter plots show Log_2_ fold changes from baseline of ATAC (x-axis) and gene expression (y-axis) for chromatin inactivated gene clusters at clinical response in Pt5 and Pt8.

For the validation cohort, we further analysed the relationship between chromatin structure and gene expression patterns using single-cell multiome analysis, which involves the simultaneous detection of ATAC and gene expression profiles. We sequenced 109,830 cells and obtained ATAC and gene expression data for the same individual cells^24^. We found that gene expression correlated with chromatin structure (Extended Data Fig. 4a–c). Furthermore, we demonstrated that changes in the chromatin structure are responsible for gene expression in tumorigenesis. We identified key genes (*P* < 0.05) for tumorigenesis that were silenced by aggregation of chromatin structure (Extended Data Fig. 4d). Integration of the corresponding ChIP–seq data subsequently supported that regions of tumour-specific silencing had accumulated H3K27me3 marks. The decrease in H3K27me3 levels was induced by valemetostat, thereby loosening chromatin structure and inducing gene expression (Extended Data Fig. 4e–g). Collectively, the results directly showed that aberrant H3K27me3 marks were eliminated in patients who received valemetostat treatment, and chromatin aggregation and gene silencing were released.

## Acquired PRC2 mutation for resistance

Although all tested cases showed a durable response, these responses were eventually interrupted due to the recurrence of ATL. We investigated how the relapse occurred, allowing ATL cells to find fitness against the epigenetic therapy.

**Fig. 3:**
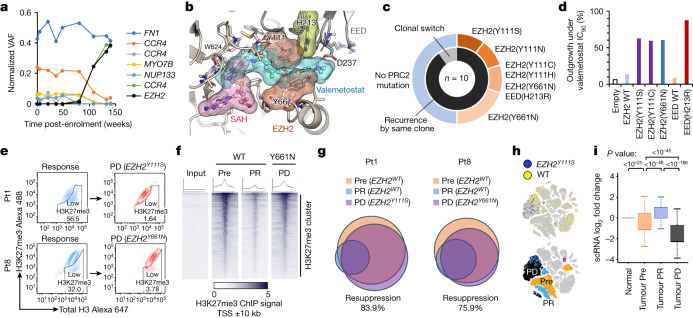
Mechanisms of resistance to valemetostat. **a**, Chronological transition of VAF values (normalized by proviral load) for somatic mutations identified by deep sequencing in Pt1 in relation to treatment with valemetostat. **b**, Model of the PRC2–valemetostat complex superimposed on the PRC2–*S*-adenosyl-l-homocysteine (SAH) complex, with molecular surfaces of ligands and mutation sites on EZH2 and/or EED identified in the clinical trials. **c**, Nested pie chart shows the proportion of PRC2 mutations and clonal characteristics at progressive disease. **d**, ATL cells (TL-Om1) with PRC2 mutations were treated with valemetostat (90% inhibitory concentration (IC_90_) or more) and monitored for outgrowth for 37 days. The bar graph shows the percentage of recovered outgrowth clones (outgrowth activity among 96 clones) for each cell with PRC2 mutations. WT, wild type. **e**, H3K27me3 staining of PBMCs in Pt1 and Pt8 gated on CD4^+^CADM1^+^CD7^−^ tumour cell populations at clinical response and at progressive disease. **f**, Heat maps of H3K27me3 ChIP–seq peaks centred on the TSS (20-kb windows) at H3K27me3 clusters in tumours from Pt8 at Pre, partial response and progressive disease (*EZH2*^*Y661N*^). **g**, Venn diagram depicts overlapped chromatin-condensed inactive genes (promoter sum < 0.01) in tumour cells from Pt1 and Pt8 at Pre, partial response and progressive disease. **h**, *t*-SNE projection of scRNA-seq data in Pt1, with cells coloured according to *EZH2*^*Y111S*^ RNA status (top) and assigned major tumour clusters (bottom). **i**, Normalized log_2_ fold changes of scRNA-seq gene expression at the chromatin-condensed inactive genes from Pt1 (scATAC-seq promoter sum < 0.01 before treatment, *n* = 1,080 genes). The middle line within box plots corresponds to the median; the lower and upper hinges correspond to the first and third quartiles; the upper whisker extends from the hinge to the largest value no further than 1.5 times the IQR; and the lower whisker extends from the hinge to the smallest value at most 1.5 times the IQR. Statistics and reproducibility are described in the Methods. Source Data

**Extended Data Fig. 5:**
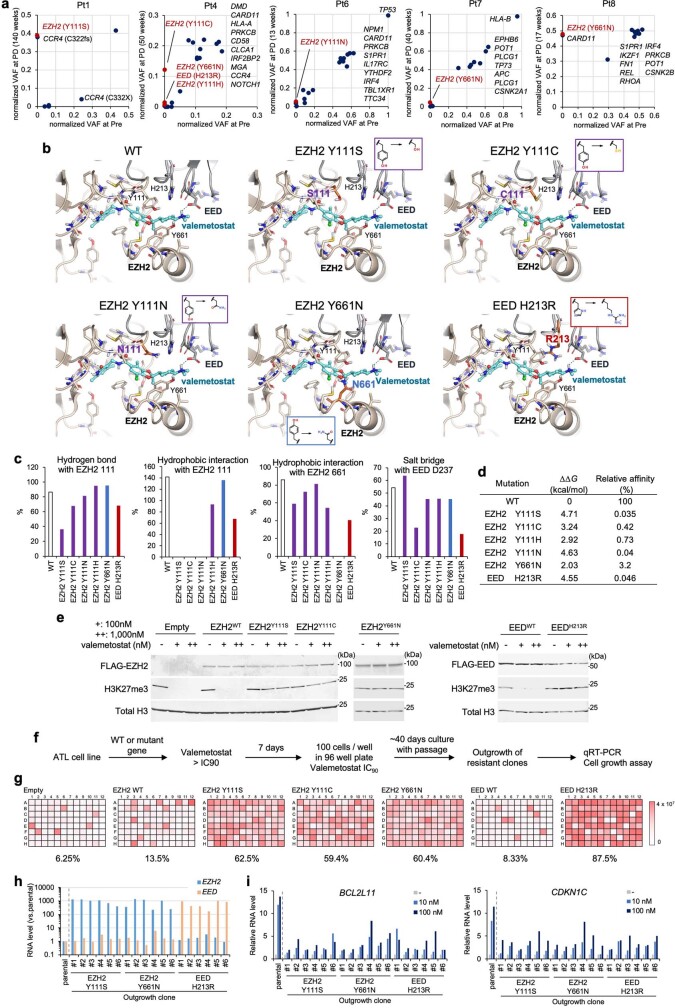
Acquired *EZH2* mutation in resistance to valemetostat. **a**, Normalized VAF values for major somatic mutations at pre-treatment (Pre, x-axis) and recurrence (PD, y-axis) in cases with acquired PRC2 mutations (*n* = 5). **b**, Valemetostat-bound PRC2 structural models for clinically identified amino acid substitutions of EZH2 (Y111S, Y111C, Y111N, Y661N) and EED (H213R). **c**, Bar charts show the normalized frequency of interactions (hydrogen bonds, hydrophobic contacts, and salt-bridges) between valemetostat and wild-type or mutant PRC2 subunits over the molecular dynamics simulation time. The bar height can be greater than 100% if the protein makes more than one contact with valemetostat. **d**, Binding free energy changes (ΔΔ*G*, kcal/mol) and relative affinity (%) of valemetostat to PRC2 mutants relative to wild-type PRC2 (WT) predicted by FEP+ simulation. **e**, H3K27me3 levels in 293 T cells expressing EZH2 and EED mutants in the presence or absence of valemetostat. Data are representative of two independent experiments. For gel source data, see Supplementary Fig. 1. **f**, Workflow for valemetostat outgrowth assay. **g**, Heatmaps represent recovered outgrowth cell numbers in TL-Om1 cells expressing PRC2 mutants in 96-well plate culture. **h**, Normalized *EZH2* and *EED* RNA levels in randomly collected outgrowth clones quantified by qRT-PCR. **i**, Parental cells and the recovered outgrowth clones were treated with valemetostat (10 nM, 100 nM) for 7 days. Bar graphs show relative expression levels of the H3K27me3 target genes (*BCL2L11* and *CDKN1C*). Source Data

We first identified a characteristic somatic mutation at the Y111 amino acid residue of EZH2 during clonal repopulation at progressive disease in Pt1 (Fig. 3a). Among the ten cases, somatic mutations were detected in the core components of the Polycomb repressive complex 2 (PRC2) gene complex around the valemetostat-binding pocket in five cases (50%) (Fig. 3b,c, Extended Data Fig. 5a,b and Supplementary Table 4). All these mutations emerged within the populations of the originating clones that existed before the treatment. Tumour-specific deep sequencing (more than 2,500 coverage) could not detect such PRC2 mutations before treatment, suggesting that the mutations had newly emerged in the original clones and propagated during treatment. These mutations have not been reported in malignant lymphomas or any other cancers in vivo. In vitro studies on other EZH2 inhibitors have reported the Y111 substitution for other amino acids^25,26^.

The clinically identified amino acid substitutions (EZH2(Y111S/Y111C/Y111H/Y111N), EZH2(Y661N) and EED(H213R)) were located at the interface among EZH2, EED and valemetostat. Binding affinities predicted using the free-energy perturbation algorithm showed that the EZH2–EED interface provides a favourable platform for valemetostat. Substitution of the amino acids would significantly reduce these interactions with valemetostat (Extended Data Fig. 5b–d). In addition, an EED mutation was identified in this study. The EED(H213R) substitution causes: (1) a reduction in the hydrophobic interaction of valemetostat with EZH2(Y111) and EZH2(Y661), and (2) a reduction in the rate of salt bridge formation with EED(D237), thus leading to a reduced relative affinity (0.046%).

We further examined the effects of mutations at the PRC2 interface on H3K27me3. In 293T cells expressing the EZH2 or EED mutants, cellular H3K27me3 levels were retained at the untreated level, even in the presence of valemetostat (Extended Data Fig. 5e). In addition, we established an ATL cell line stably expressing PRC2 mutants and performed a resistant outgrowth assay^27,28^ in the presence of valemetostat. We found that PRC2 mutations caused the emergence of tolerant cells. These emerged cells were less sensitive to the epigenetic response invoked by valemetostat and thus were less susceptible to reactivation of the target TSGs (Fig. 3d and Extended Data Fig. 5f–i). Direct evaluation of the H3K27me3 level in the progressive disease clinical clones harbouring *EZH2*^*Y111S*^ (Pt1) and *EZH2*^*Y661N*^ (Pt8) further confirmed that tumour cells in a hypermethylated state were repopulating. The H3K27me3-low cells detected during the clinical response almost disappeared (Fig. 3e). In addition, H3K27me3 ChIP–seq showed that these repopulated cells with the resistant mutation resumed the similar pattern detected before treatment, indicating that the recovery of H3K27me3 caused clinical relapse (Fig. 3f).

We further examined the effects of the acquired mutations on the chromatin structure. The scATAC-seq timelapse data showed that the promoters inactivated before the treatment were significantly reactivated by valemetostat. Upon the occurrence of the mutation, strong chromatin compaction of the same gene set occurred in the rapidly propagating resistant clone, demonstrating a marked tendency to revert to the original chromatin structure (Fig. 3g). Furthermore, the scRNA-seq analysis confirmed the expression of the mutant *EZH2* in the progressive disease clone (Fig. 3h). In this progressive disease clone, the expression of epigenetically inactivated genes before the treatment was also strongly silenced, corresponding well with the scATAC-seq data (Fig. 3i). The evidence collectively indicates that the clonally selected mutations in PRC2 genes should be responsible for resistance, almost completely reversing the effects of valemetostat by chromatin recondensation.

## Epigenetic homeostasis by DNA methylation

**Fig. 4:**
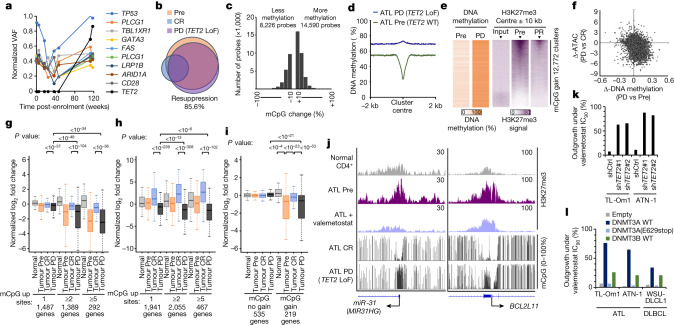
Non-genetic mechanisms of resistance to valemetostat. **a**, Chronological transition of normalized VAF values for somatic mutations in Pt3. **b**, Venn diagram depicts overlapped chromatin-condensed inactive genes (promoter sum < 0.01) in tumour cells from Pt3. LoF, loss of function. **c**, Histogram shows differentially methylated (ΔmCpG < −10% or ΔmCpG > 10%) probes in resistant tumour from Pt3 at progressive disease (118 weeks) versus pre-treatment tumour. **d**, Whole-genome DNA methylation profiling detected progressive disease-associated mCpG acquisition clusters. The plot shows average DNA methylation (%) in tumour baseline (Pre) and at progressive disease centred by mCpG gain 12,772 clusters. **e**, Heat maps of DNA methylation and H3K27me3 ChIP–seq peaks (20-kb windows) at progressive disease-associated mCpG gain clusters. The arrowheads indicate cluster centre. **f**, Correlation between Δ-DNA methylation (%) and Δ-scATAC-seq promoter sum in the resistant cells versus pre-treatment cells from Pt3. **g**,**h**, Normalized log_2_ fold changes of scATAC-seq promoter activities (**g**) and scRNA-seq gene expression (**h**) in relation to treatment-associated mCpG gain in Pt3. Statistical significance is provided only for main combinations. **i**, Normalized log_2_ fold changes of scATAC-seq promoter activities of TSGs. Statistical significance is provided only for main combinations. In **g**–**i**, the middle line within box plots corresponds to the median; the lower and upper hinges correspond to the first and third quartiles; the upper whisker extends from the hinge to the largest value no further than 1.5 times the IQR; and the lower whisker extends from the hinge to the smallest value at most 1.5 times the IQR. **j**, Representative tracks for H3K27me3 (ChIP–seq) and methylated CpG tracks (EM-seq) (16 weeks (complete response) and 118 weeks (progressive disease) with *TET2* LoF) around the TSS. **k**,**l**, Bar graphs show the percentage of recovered outgrowth clones (outgrowth activity among 96 clones) under the valemetostat IC_90_ or higher condition for each lymphoma cell with *TET2* knockdown (**k**) and ectopic DNMT3A or DNMT3B expression (**l**). DLBCL, diffuse large B cell lymphoma; shCtrl, control shRNA. Statistics and reproducibility are described in the Methods. Source Data

**Extended Data Fig. 6:**
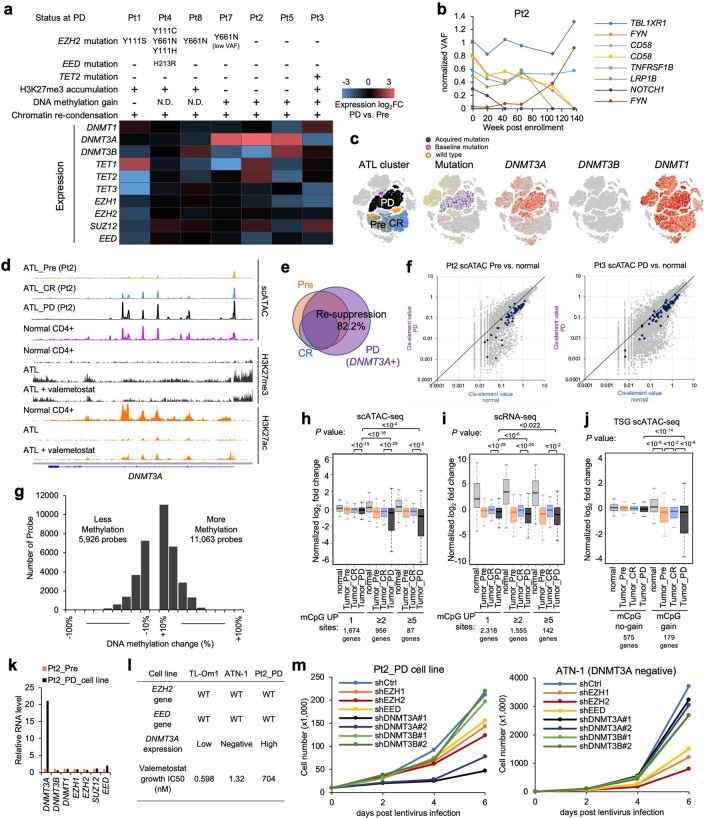
Acquired *DNMT3* overexpression in resistance to valemetostat. **a**, Characteristics of clinically resistant clones based on single-cell analysis and genomic profiling. **b**, Chronological transition of normalized VAF values for somatic mutations in Pt2 in relation to treatment with valemetostat. **c**, tSNE projection for Pt2 scRNA-seq data. *DNMT3A* expression was upregulated in the recurrent clone (*P* < 10^−9^). **d**, scATAC-seq and ChIP-seq tracks at *DNMT3A* locus. **e**, Venn diagram depicts overlapped chromatin-condensed inactivated genes (Promoter sum <0.01) in Pt2 tumor cells at Pre, CR, and PD (*DNMT3A* expression). **f**, Scatter plots show all gene promoter activities in recurrent clones (y-axis) in Pt2 (left) and Pt3 (right) versus corresponding normal cells (x-axis). The gene loci with decreased copy numbers as defined from the WGS data are indicated in dark purple. **g**, Histogram shows differentially methylated (ΔmCpG <−10%, or >10%) probes in Pt2 resistant tumor at PD (135 weeks) versus pre-treatment tumor. **h**, **i**, Boxplots summarize normalized log_2_ fold changes of scATAC-seq promoter activities (**h**) and scRNA-seq gene expression (**i**) in relation to treatment-associated mCpG gain in Pt2. The genes for which integrated data were available were evaluated. Statistical significance is provided only for main combinations. **j**, Normalized log_2_ fold changes of scATAC-seq promoter activities of tumor suppressor genes (TSG) in relation to treatment-associated mCpG gain in Pt2. The genes for which integrated data were available were evaluated. Statistical significance is provided only for main combinations. **k**, Pt2-derived resistant cell line was successfully established. The same clonal origin and the absence of PRC2 mutations were confirmed by targeted sequencing. Bar graph shows relative RNA levels of DNMT family and PRC2 genes. **l**, Table summarizes characteristics of ATL cell lines. Pt2_PD cells showed low sensitivity to valemetostat. **m**, Knockdown and DNMT family and PRC2 genes were induced by lentivirus-mediated shRNA. After lentivirus infection, growth cell numbers for 6 days were calculated. The middle lines within box plots correspond to the medians; lower and upper hinges correspond to the first and third quartiles. The upper whisker extends from the hinge to the largest value no further than 1.5 * IQR. The lower whisker extends from the hinge to the smallest value at most 1.5 * IQR. Statistics and reproducibility are described in Methods. Source Data

The characteristics of the resistant clones are summarized in Extended Data Fig. 6a. We noticed that some patients showed a relapse even though the tumour levels of H3K27me3 were maintained low. In those cases, whole-genome sequencing and deep sequencing of the recurrent clones in the other patients showed no acquired mutations in PRC2 genes (Fig. 3c). Nevertheless, the recurrent clones also genetically evolved, showing an increased single-nucleotide variant/insertion and deletion and copy number variations (Supplementary Tables 2 and 3). Focusing on the epigenome-related genes, we detected a biallelic loss of function of the *TET2* gene in the selected progressive disease clone in Pt3 (Fig. 4a). The *TET2* mutation could be rarely found in ATL^16^ and was not detected before the treatment, suggesting that the mutation was acquired during the treatment-induced selection. In Pt2, although no epigenome-related gene mutations could be detected, the scRNA-seq analysis identified robust *DNMT3A* expression, specifically in the recurrent clone (Extended Data Fig. 6b,c). The scATAC-seq data showed that the enhancers of the *DNMT3A* locus were activated and epigenetically evolved in the recurrent clone (Extended Data Fig. 6d). In the validation cohort, patients with no detectable PRC2 mutations (Pt2 and Pt5) and a patient with an *EZH2* mutation but low variant allele frequency (Pt7) showed elevated *DNMT3A* and decreased *TET2* levels (Extended Data Fig. 6a).

**Extended Data Fig. 7:**
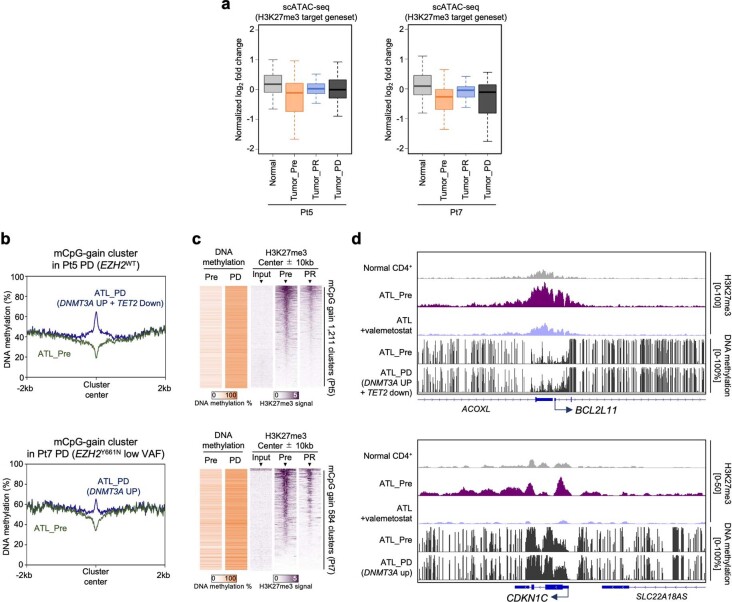
DNA methylation profiling in phase 2 cohort. **a**, Boxplots summarize normalized log_2_ fold changes of scATAC-seq promoter activities at H3K27me3 target genes (*n* = 630) in Pt5 and Pt7. **b**–**d**, Whole genome DNA methylation profiling (28.3 M CpG sites/sample on average) detected PD-associated mCpG acquisition clusters in Pt5 (upper) and Pt7 (lower). Plots show average DNA methylation (%) in tumor baseline (Pre) and at PD centered by mCpG gain clusters (**b**). Heatmaps depict DNA methylation and H3K27me3 ChIP-seq peaks (20-kb windows) at PD-associated mCpG gain clusters (**c**). Representative tracks show H3K27me3 and methylated CpG tracks around the TSS of tumor suppressor genes (**d**). The middle lines within box plots correspond to the medians; lower and upper hinges correspond to the first and third quartiles. The upper whisker extends from the hinge to the largest value no further than 1.5 * IQR. The lower whisker extends from the hinge to the smallest value at most 1.5 * IQR. Statistics and reproducibility are described in Methods.

We interpreted the evolutionary impact of these epigenetic factors from scATAC-seq and found that the recurrent clones without PRC2 mutations also showed chromatin re-aggregation (Fig. 4b and Extended Data Figs. 6e and 7a). By contrast, the acquired copy number reduction observed after the relapse was more common at the active loci and not associated with the gene inactivation (Extended Data Fig. 6f).

Considering that abnormalities in the DNA methylation pathway were detected, we investigated the relationship between chromatin recondensation and DNA methylation in relapse. We performed the 850K DNA methylation array to quantify methylated CpG (mCpG) levels near the TSS. The progressive disease cells without PRC2 mutations showed increased DNA methylation near the TSS, where H3K27me3 was enriched (Fig. 4c and Extended Data Fig. 6g). We further profiled the whole-genome DNA methylation status of 28.3 million CpG sites per sample on average (96.4% of all CpG sites) using EM-seq and detected progressive disease-associated mCpG acquisition clusters in the target region of valemetostat where H3K27me3 originally accumulated (Fig. 4d,e and Extended Data Fig. 7b–d). Progressive disease cells (EZH2(Y111S)) from Pt1 showed no such preferential mCpG distribution.

We also examined the influence of mCpG on the open chromatin structure and identified a moderate negative correlation between the increase in mCpG with recurrence and chromatin accessibility (*R* = −0.421). The genes characterized by the H3K27me3-related chromatin condensation were restored by valemetostat but were again strongly recondensed by mCpG gain at progressive disease (Fig. 4f,g and Extended Data Fig. 6h). The corresponding scRNA-seq data confirmed that this epigenetic transition was reflected in the gene expression (Fig. 4h and Extended Data Fig. 6i). Consequently, the gene regulation of several important TSGs restored by valemetostat was again silenced by focal compensatory mCpG acquisition (Fig. 4i,j and Extended Data Figs. 6j and 7d).

To directly verify that the DNA hypermethylation was responsible for resistance, we established a Pt2-derived progressive disease cell line. The same clonal origin and absence of PRC2 mutations were confirmed, and this cell line showed high *DNMT3A* expression and low sensitivity to valemetostat. Cell growth was inhibited by *DNMT3A*-targeting short hairpin RNA (shRNA), indicating that the cells became dependent on DNMT3A rather than on PRC2 (Extended Data Fig. 6k–m).

## DNMT3A and TET2 in acquired resistance

**Extended Data Fig. 8:**
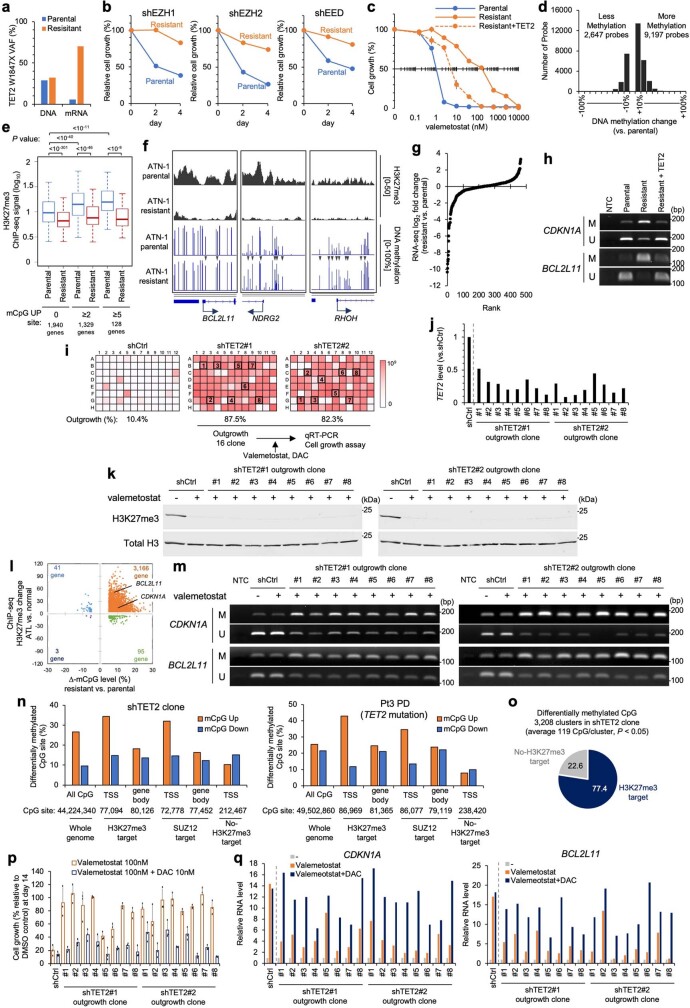
Establishment and characterization of TET2-deficient valemetostat resistant models. **a**, ATL cell lines were cultured in growth media supplemented with 10 nM of valemetostat for two months. Inhibitor-resistant outgrowth was observed at 100 nM. Bar graph shows VAF values of *TET2*^W1847X^ DNA and expressed mRNA in ATN-1 parental and valemetostat-resistant cells. **b**, shRNA targeting EZH1, EZH2, or EED were introduced by lentivirus vectors in parental and resistant cell line (ATN-1_R). Graphs show cell growth (%) relative to control shRNA. **c**, Growth inhibition rate (%) by valemetostat (0 ~ 10,000 nM) in ATN-1 parental and valemetostat-resistant cells. *n* = 3 independent experiments, mean ± SD. For gene rescue experiment, *TET2* cDNA were transduced by lentivirus vector. **d**, Histograms show differentially methylated (ΔmCpG <−10%, or > 10%) probes in resistant ATN-1 versus parental cells. **e**, **f**, H3K27me3 occupancy was analyzed by ChIP-seq for the parental and valemetostat-resistant ATN-1 cells. Boxplot shows H3K27me3 log_10_ signals in relation to resistance-associated mCpG gain (**e**). The genes for which integrated data were available were evaluated. Statistical significance is provided only for main combinations. Representative tracks for H3K27me3 and methylated CpG tracks are shown in (**f**). Arrowheads indicate representative CpG sites with methylation gain. **g**, Log_2_ fold-changes of RNA-seq expression level (TPM) at mCpG gain genes (mCpG UP sites > 2) in resistant ATN-1 cells compared to parental cells. **h**, MSP was performed for DNA isolated from parental or resistant ATN-1 cells in the presence or absence of *TET2*. Amplified DNA was visualized by agarose gel electrophoresis for TSG loci with primer sets specific for methylated state (M) or unmethylated state (U). Data are representative of two independent experiments. NTC: no template control. **i**, Heatmaps represent recovered outgrowth cell numbers in ATN-1 cells expressing shTET2 (#1, #2) in 96-well plate culture. Collected outgrowth clones (*n* = 16) are indicated. **j**, *TET2* RNA level in randomly collected outgrowth clones quantified by qRT-PCR. **k**, H3K27me3 level in valemetostat outgrowth clones. **l**, Scatter plot shows DNA methylation changes (x-axis) and accumulation of H3K27me3 (y-axis) in the promoter proximal region (TSS ± 1 kbp) of each gene in the outgrowth shTET2 clone #1. Values are averaged per gene and represented only differentially methylated genes (ΔmCpG < −5% or >5%). **m**, MSP assay for H3K27me3 target genes (*CDKN1A* and *BCL2L11*) in valemetostat outgrowth clones. **n**, Bar graphs show differentially methylated CpG sites in shTET2 outgrowth clone (left) and Pt3 PD clone (right) in single nucleotide resolution analysis using EM-seq data. Percentages were compiled from all CpG sites (filter depth > 5) in the TSS and downstream gene body regions (center ± 1 kbp). Target genes were defined based on H3K27me3, SUZ12, and H3K27ac ChIP-seq data. **o**, Pie chart shows the percentage of epigenomic domains of CpG islands near the TSS with increased methylation (*P* < 0.05). **p**, **q**, Control cells (shCtrl) and the recovered outgrowth clones were treated with valemetostat (100 nM) and DAC (10 nM). Bar graphs show relative cell growth at 14 days (**p**, *n* = 3, independent experiments, mean ± SD) and relative expression levels of the H3K27me3 target genes (*CDKN1A* and *BCL2L11*) at 7 days (**q**). The middle lines within box plots correspond to the medians; lower and upper hinges correspond to the first and third quartiles. The upper whisker extends from the hinge to the largest value no further than 1.5 * IQR. The lower whisker extends from the hinge to the smallest value at most 1.5 * IQR. Statistics and reproducibility are described in Methods. For gel source data, see Supplementary Fig. 1. Source Data

To validate whether DNA methylation was induced by the epigenetic selective pressure of the H3K27me3 change, we established two resistant ATL cell lines by long-term exposure (over 2 months) to valemetostat. ATN-1 cells were transformed into resistant cells preferentially expressing loss-of-function *TET2* mRNA, similar to the results observed in Pt3 and Pt5. This resistant clone showed a low sensitivity to PRC2 knockdown and treatment with valemetostat. Instead, the *TET2* gene transfer increased sensitivity to valemetostat, suggesting that the clone is dependent on *TET2* (Extended Data Fig. 8a–c). mCpG was increased near the TSS (Extended Data Fig. 8d). ChIP–seq and RNA-seq for the resistant cell lines showed that although the H3K27me3 level remained low even after valemetostat removal, a high mCpG level on the H3K27me3 sites still had a compensatory role in repressing the expression (Extended Data Fig. 8e–g). Methylation-specific PCR supported the role of TET2 in the regulation of DNA methylation (Extended Data Fig. 8h).

We also collected data to clarify the function of TET2 in the acquired resistance to valemetostat by molecular genetic analyses. ATN-1 cells with *TET2* knockdown were cultured for an extended period (2 months) to induce epigenetic evolution. The *TET2*-knockdown cells showed a significant resistant outgrowth capacity and a large number of resistant clone cells emerged in the presence of valemetostat (Fig. 4k and Extended Data Fig. 8i–k). The emerged outgrowth clones showed reduced *TET2* expression and low levels of H3K27me3. Whole-genome profiling by EM-seq and methylation-specific PCR-based validation in multiple clones demonstrated the compensatory mCpG acquisition at H3K27me3 targets (Extended Data Fig. 8l,m). Single-nucleotide resolution analysis of the *TET2*-targeting shRNA and clinically resistant Pt3 clones preferentially detected mCpG gain in the TSS regions of Polycomb targets (H3K27me3 and SUZ12) but not in outside regions (Extended Data Fig. 8n and Supplementary Table 5). Furthermore, clustered methylation at 3,208 CpG islands near the TSS was detected (*P* < 0.05), many of which were identified in H3K27me3 targets (Extended Data Fig. 8o). Interestingly, these cells were sensitive to a low dose of the DNA methylation inhibitor decitabine. Growth inhibition and the reactivation of key TSGs were detected in all 16 tested clones (Extended Data Fig. 8p,q).

**Extended Data Fig. 9:**
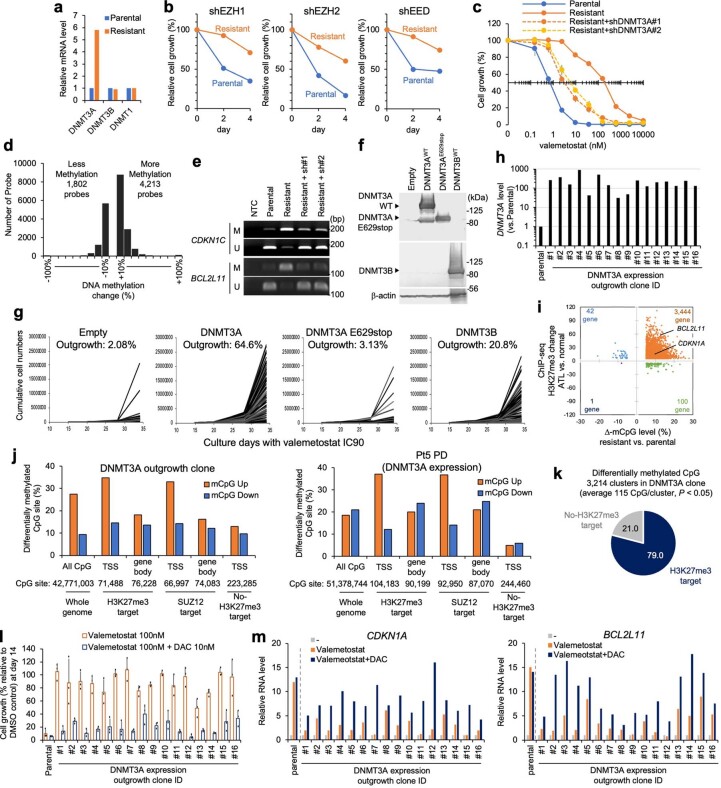
Establishment and characterization of DNMT3A-dependent valemetostat resistant models. **a**, Relative expression levels of *DNMT* family genes in TL-Om1 parental and valemetostat-resistant cells quantified by qRT-PCR. **b**, shRNA targeting EZH1, EZH2, or EED were introduced by lentivirus vectors in parental and resistant cell line (TL-Om1_R). Graphs show cell growth (%) relative to control shRNA. **c**, Growth inhibition rate (%) by valemetostat (0 ~ 10,000 nM) in TL-Om1 parental and valemetostat-resistant cells. shRNA targeting DNMT3A or DNMT3B were transduced by lentivirus vector. *n* = 3, independent experiments, mean ± SD. **d**, Histograms show differentially methylated (ΔmCpG <−10%, or > 10%) probes in resistant TL-Om1 versus parental cells. **e**, MSP assay in TL-Om1_R with shDNMT3A. Amplified DNA was visualized by agarose gel electrophoresis for TSG loci with primer sets specific for methylated state (M) or unmethylated state (U). Data are representative of two independent experiments. **f**, DNMT3A (WT and E629stop which lacks enzymatic domain) and DNMT3B expressing cell models were established by lentivirus vectors in ATL-derived TL-Om1 and ATN-1 cells and DLBCL-derived WSU-DLCL2 cells. DNMT3A and DNMT3B protein levels were analyzed by immunoblotting (ATN-1). Data are representative of two independent experiments. **g**, Cell growth curves show recovered outgrowth cell numbers in ATN-1 cells in 96-well plate culture. **h**, *DNMT3A* RNA level in randomly collected outgrowth clones (*n* = 16) quantified by qRT-PCR. **i**, Scatter plot shows DNA methylation changes (x-axis) and accumulation of H3K27me3 (y-axis) in the promoter proximal region (TSS ± 1 kbp) of each gene in the outgrowth DNMT3A clone #1. Values are averaged per gene and represented only differentially methylated genes (ΔmCpG < −5% or >5%). **j**, Bar graphs show differentially methylated CpG sites in DNMT3A outgrowth clone (left) and Pt5 PD clone (right) in single nucleotide resolution analysis using EM-seq data. Percentages were compiled from all CpG sites (filter depth > 5) in the TSS and downstream gene body regions (center ± 1 kbp). Target genes were defined based on H3K27me3, SUZ12, and H3K27ac ChIP-seq data. **k**, Pie chart shows the percentage of epigenomic domains of CpG islands near the TSS with increased methylation (*P* < 0.05). **l**, **m**, Parental cells and the recovered outgrowth clones were treated with valemetostat (100 nM) and DAC (10 nM). Bar graphs show relative cell growth at 14 days (**l**, *n* = 3, independent experiments, mean ± SD) and relative expression levels of the H3K27me3 target genes (*CDKN1A* and *BCL2L11*) at 7 days (**m**). Statistics and reproducibility are described in Methods. For gel source data, see Supplementary Fig. 1. Source Data

By long-term exposure to valemetostat, we also developed another resistant cell line with increased *DNMT3A* expression and hypermethylated CpG sites, as observed in Pt2, Pt5 and Pt7. This resistant cell model showed a low sensitivity to PRC2 knockdown and treatment with valemetostat, and susceptibility was resumed by shRNA experiments against DNMT3A. The resistance-associated DNA methylation was cancelled by *DNMT3A*-targeting shRNA (Extended Data Fig. 9a–e).

Furthermore, forced expression of DNMT3A alone caused a robust resistant outgrowth in different lymphoma models (Fig. 4l and Extended Data Fig. 9f–h). However, the outgrowth was completely inhibited when the catalytically active enzymatic domain of DNMT3A was deleted. This effect was also detected in DNMT3B-expressing cells and, although to a lesser extent, in all cell lines. Indeed, all the randomly selected clones (*n* = 16) showed a high DNMT3A level and were resistant to valemetostat. EM-seq demonstrated that mCpG was compensatory acquired in H3K27me3 targets, with increased methylation of mCpG clusters (Extended Data Fig. 9i–k and Supplementary Table 5). Co-treatment with decitabine significantly inhibited cell growth and reactivated epigenetically suppressed TSGs (Extended Data Fig. 9l,m). These results indicated that DNMT3A causes an epigenetic acquisition of resistance.

Overall, we concluded that despite using different genes, the common chromatin structure-based mechanism was used to acquire the resistance.

## Subpopulations with differential susceptibility

All abnormalities detected in resistant cells were heritable traits selected after several months under constant treatment-related pressure. The slow emergence of such heritable clones indicates the existence of primary tolerance caused by differences in susceptibility.

**Fig. 5:**
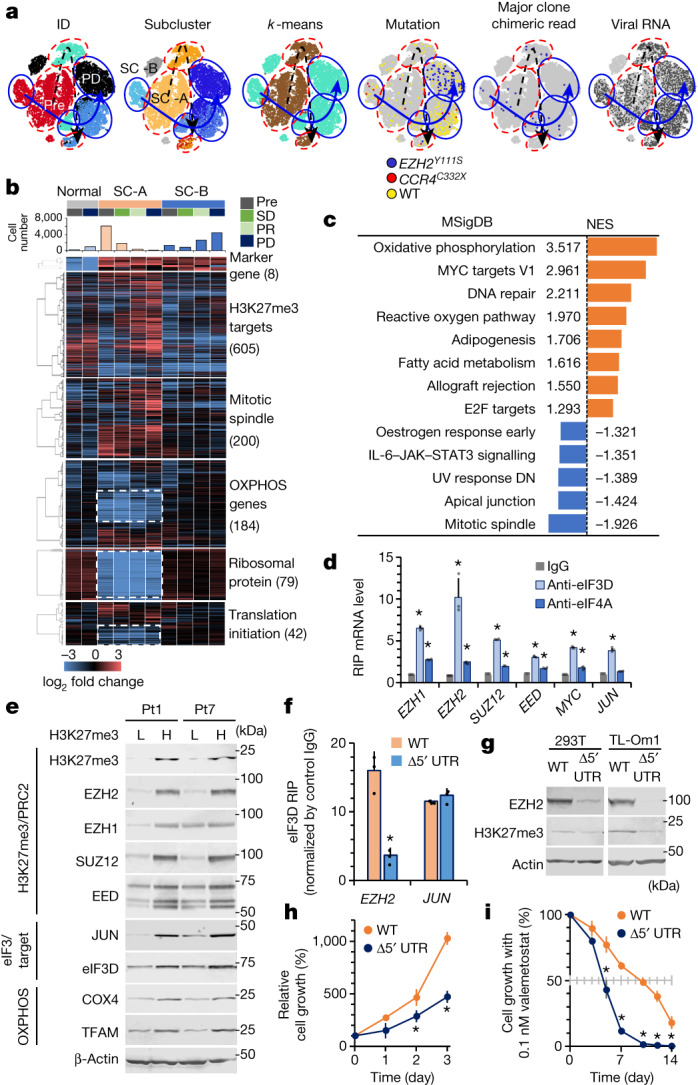
Intrinsic subpopulations with differential susceptibility. **a**, *t*-SNE projection of scRNA-seq data in Pt1, with cells coloured according to sample ID, subclustering based on clinical time order or *k*-means, and profiles of mutations and virus reads. Black dashed arrows indicate clinical time order of SC-A; blue solid arrows indicate clinical time order of SC-B. **b**, Clustered heat maps depict expression levels of genes involved in differentially enriched categories in subclusters SC-A and SC-B in Pt1. Genes highlighted by white dashed lines indicate genes significantly decreased in SC-A. SD, stable disease. **c**, Hallmark gene set enrichment analysis of scRNA-seq data from Pt1 SC-B before valemetostat treatment compared with SC-A. For all pathways shown, significantly enriched gene sets were evaluated by normalized enrichment score (NES) and nominal *P* value (*P* < 0.001). DN, down-regulated. **d**, RIP assay for PRC2 gene mRNA. eIF complexes were immunopurified from TL-Om1 cells using antibodies to eIF3D and eIF4A. eIF-associated mRNA was quantified by quantitative PCR. The graph shows the fold change in the enrichment relative to the control IgG. *n* = 3 independent experiments, mean ± s.d., *P < 0.05. **e**, Immunoblots show protein levels of H3K27me3, PRC2, eIF3D and OXPHOS mitochondrial factors in H3K27me3 higher (H) and lower (L) cells from Pt1 and Pt7 after valemetostat treatment. Electrophoresis experiments with independent patient samples were performed once. **f**, Quantification of eIF3D-bound PRC2 gene mRNA in 293T cells with *EZH2* WT and *EZH2* Δ5′ UTR by RIP assay. *n* = 3 independent experiments, mean ± s.d., *P = 0.00861. **g**, Protein levels of EZH2 and H3K27me3 in cells with *EZH2* WT and *EZH2* Δ5′ UTR. **h**, Relative cell growth rate (%) over time in TL-Om1 cells with *EZH2* WT and *EZH2* Δ5′ UTR. *n* = 3 independent experiments, mean ± s.d., *P < 0.05. **i**, Growth inhibition rate (%) over time by 0.1 nM valemetostat in TL-Om1 cells with *EZH2* WT and *EZH2* Δ5′ UTR. *n* = 3 independent experiments, mean ± s.d., *P < 0.05. Statistics and reproducibility are described in the Methods. For gel source data, see Supplementary Fig. 1. Source Data

**Extended Data Fig. 10:**
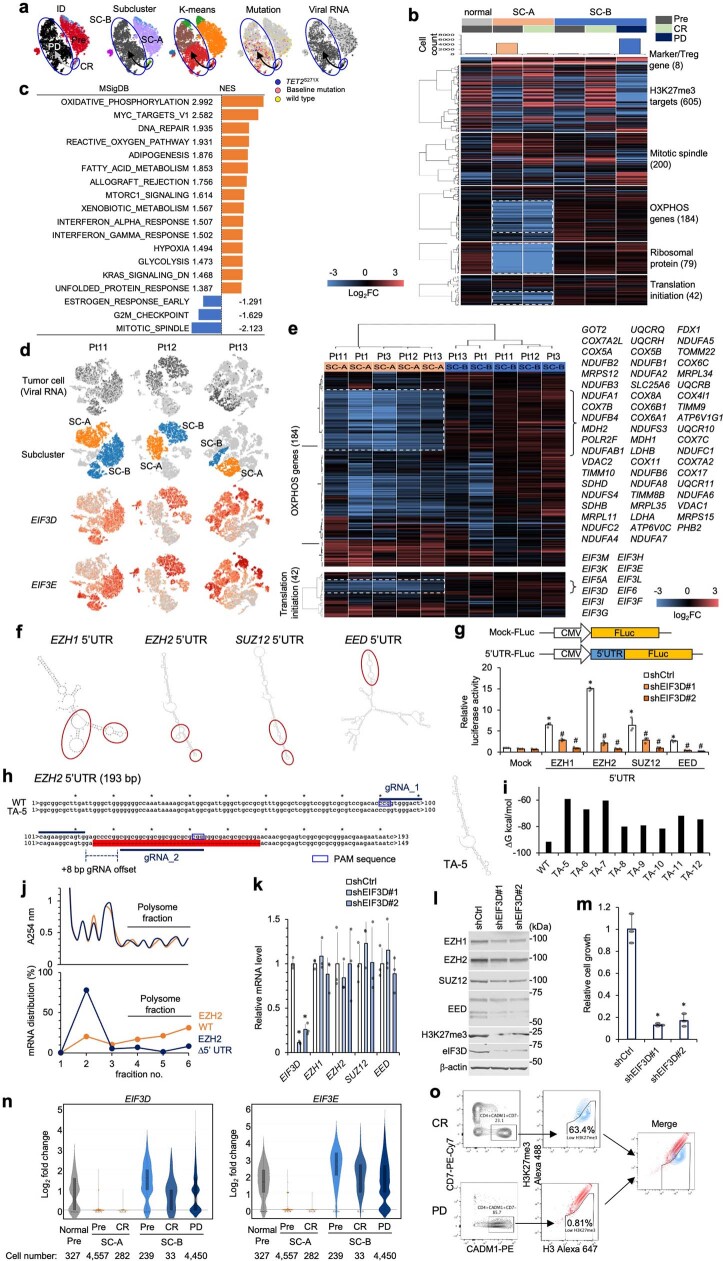
Intrinsic subpopulations and translational regulation of PRC2 genes. **a**, t-SNE projection of scRNA-seq data in Pt3, with cells colored according to sample ID, sub-clustering based on clinical time order or K-means, and profiles of mutations and virus reads. **b**, Clustered heatmaps depict expression levels of genes involved in differentially enriched categories in subclusters SC-A and SC-B in Pt3. **c**, Hallmark GSEA of scRNA-seq data from Pt3 SC-B before valemetostat treatment compared with SC-A. For all pathways shown, significantly enriched gene sets were evaluated by normalized enrichment score (NES) and nominal *P* value (*P* < 0.001). **d**, t-SNE projection of scRNA-seq data in Pt11, Pt12, and Pt13, with cells colored according to subclusters, viral reads, and expression of *EIF3D* and *EIF3E*. **e**, Clustered heatmaps depict expression levels of genes involved in OXPHOS and translation initiation in subclusters SC-A and SC-B in ATL patients (*n* = 5). **f**, Secondary structures of 5′UTR of PRC2 factor genes. Red circles indicate representative stem-loop structures easily recognized by eIF3. **g**, Reporter-based 5′UTR activity screening. A series of 5′UTR -luciferase reporters was transfected in 293 T cells with or without shEIF3D shRNA vector. Relative values of dual-luciferase assay are presented. *n* = 3, independent experiments, mean ± SD. *, versus Mock, *P* < 0.05; #, versus shCtrl, *P* < 0.05. **h**, **i**, CRISPR-nickase (Cas9 D10A)-based deletion of endogenous *EZH2* 5′UTR. Representative *EZH2* 5′UTR sequence of an established clone (TA-5) and gRNA regions are illustrated (**h**). Estimated mfold free energies (ΔG kcal/mol) of established Δ5′UTR clones are shown by bar graph (**i**). **j**, Translation activity was evaluated by polysome analysis. The amount of mRNA in the ribosomal and polysomal fractions was quantified using sucrose density gradient centrifugation. Absorbance at 254 nM of collected fractions (upper) and *EZH2* mRNA level (% distribution, lower) are shown (representative data, *n* = 2 biological replicates). **k**–**m**, Effect of eIF3D knockdown (KD) in TL-Om1 cells. The qRT-PCR (**k**) and immunoblotting (**l**) showed reduced expression of PRC2 genes and H3K27me3 at the protein level. The eIF3D KD cells showed reduced growth activity (**m**). *n* = 3 independent experiments, mean ± SD, * *P* < 0.05. **n**, Violin and box plots show scRNA-seq expression distribution of *eIF3D* and *eIF3E* genes in Pt3. **o**, H3K27me3 staining of PBMCs in Pt3 gated on CD4^+^/CADM1^+^/CD7^−^ tumor cell populations at 16 weeks (CR) and 118 weeks (PD) post valemetostat treatment. The middle lines within box plots correspond to the medians; lower and upper hinges correspond to the first and third quartiles. The upper whisker extends from the hinge to the largest value no further than 1.5 * IQR. The lower whisker extends from the hinge to the smallest value at most 1.5 * IQR. Statistics and reproducibility are described in Methods. For gel source data, see Supplementary Fig. 1. Source Data

Reclustering the scRNA-seq data, including those remaining after treatment, yielded two subclusters with mutually distinct expression patterns (Fig. 5a and Extended Data Fig. 10a). The two subclusters at Pre shared the same major somatic mutations and viral integration sites, indicating that they were originally derived from the same clone. Comparing the characteristics of these subpopulations, along with a ‘clinical time order’, revealed that subcluster B (SC-B) was infrequent before treatment and at the time of response. However, the SC-B pre-existed when the eventual resistant somatic mutations emerged. The clone, which subsequently expanded and caused the relapse by acquiring the resistant mutation, shared the same mutation patterns with SC-B. Therefore, SC-B should be the origin of the resistant clones. The two clusters did not differ significantly in the expression of regulatory T lineage marker genes, which is characteristic of ATL cells. H3K27me3 target gene expression was slightly lower in SC-B than in SC-A at Pre, and this feature was maintained all the time until progressive disease (Fig. 5b and Extended Data Fig. 10b).

To characterize the molecular features differentiating SC-B from SC-A as the possible origin of the progressive disease clone, we conducted a gene set enrichment analysis. Significant enrichment of the genes associated with metabolism was observed in SC-B. In particular, the gene expression levels were higher for oxidative phosphorylation (OXPHOS) genes and mitochondria-related genes in SC-B (Fig. 5c and Extended Data Fig. 10c). These characteristics were maintained until the relapse phase. Furthermore, the expression of the ribosomal protein genes differed significantly between these two clusters. We inspected the genes associated with translation initiation and found that SC-B exhibited a relatively high expression of the eIF3 family genes. As eIF3D and eIF3E are responsible for promoting the translation of genes related to the metabolic pathways^29,30^, this result was consistent with the characteristics of OXPHOS.

To characterize the transcriptional features, we analysed previous scRNA-seq data from another cohort^18^ (*n* = 3). Results showed that similar subpopulations were observed in other cases, indicating the presence of such heterogeneity (Extended Data Fig. 10d,e). SC-B showed enhanced OXPHOS characteristics and expression of eIF3 genes. Of note, the different transcriptome was not supported as changes in chromatin accessibility in the corresponding scATAC-seq data. Thus, this heterogeneity appears to be a plastic feature that is not epigenetically defined.

The 5′ untranslated regions (UTRs) of PRC2 genes were predicted to be bound to eIF3D^31^ (Extended Data Fig. 10f). Insertion of the 5′ UTR into the upstream of luciferase resulted in increased expression, which was attenuated by eIF3D knockdown (Extended Data Fig. 10g). Furthermore, RNA immunoprecipitation (RIP) of H3K27me3-high ATL cells showed that the mRNAs of PRC2 factors were selectively captured by eIF3D as much or more than *JUN* mRNA, which is a known target of eIF3D^30,31^ (Fig. 5d). To directly examine how tumour cell subpopulations identified in scRNA-seq may be linked to responsiveness to valemetostat, cells with depleted or relatively high H3K27me3 levels were sorted from two samples after administration but before acquisition of *EZH2* mutations (see Methods)^32^. The results showed that the low-susceptible cell population had high protein levels of OXPHOS mitochondrial factors (COX4 and TFAM), as well as eIF3 and their targets such as PRC2 factors and JUN (Fig. 5e).

To examine the role of the 5′ UTR, we established EZH2 5′ UTR deletion models (Δ5′ UTR) by expressing two adjacent guide RNAs and CRISPR-nickase (Cas9 D10A)^33^. The 5′ UTR in the generated cells lost the bulb structure necessary for binding with eIF3D and were also less stable (Extended Data Fig. 10h,i). RIP of the Δ5′ UTR cells showed that *EZH2* mRNA selectively reduced incorporation into the eIF3D complex and decreased polysome formation and translational efficacy (Fig. 5f,g and Extended Data Fig. 10j). EZH2 and H3K27me3 levels were decreased. The Δ5′ UTR ATL cells also showed decreased proliferative capacity and early response to a low concentration of valemetostat, thereby indicating that the enhanced eIF3 activity and the 5′ UTR of EZH2 are involved in sensitivity (Fig. 5h,i). Moreover, knockdown of eIF3D reduced PRC2 proteins and H3K27me3 and significantly decreased cellular proliferative activities (Extended Data Fig. 10k–m). Consistent with these data, progressive disease cells in Pt3 repopulated as eIF3D-high expressing cells showed characteristics of resistant cells with high H3K27me3 (Extended Data Fig. 10n,o). These results support the idea that the transcriptional differences in the subclusters are involved in valemetostat sensitivity.

Note that valemetostat was sufficiently effective in Pt2 who showed high OXPHOS and eIF3D/eIF3E. The clinically expected tumour reduction was also achieved, even in the presence of SC-B in Pt1 and Pt3. Although these non-genetic and non-epigenetic features may confer differences in relative susceptibility, they do not appear to act directly on resistance.

## Conclusions

This study illustrates the molecular and cellular dynamics in patients in response to an inhibitor designed for histone methyltransferases. Integration of the multilayered omics analyses and clinical resources revealed that eliminating H3K27me3 leads to the reprogramming of the cancer epigenome, thereby exerting a sustained clinical benefit. This concept is consistent with previous reports indicating that H3K27me3 exhibits a primary role in chromatin compaction^1,34^. The genome-wide chromatin decondensing directly leads to the restoration of TSG and could even enhance the efficacy of cytotoxic agents^35^. Furthermore, both treatment-naive and treatment-adapted patients displayed the characteristic condensed chromatin structures, suggesting that chromatin compaction is indispensable for tumour maintenance and growth. The resistant mutations appeared on the docking interface of EZH2 itself or on very pivotal epigenome factors with cooperative roles in gene silencing, which is particularly intriguing. Moreover, resistance emergence took a long time. The cancer cells may have limited path options to escape from the inhibitor. If that would be the case, the potential for applying combination therapies to target epigenomic abnormalities could be more significant than that of other previous anticancer drugs^36,37^. We hope that the epigenetic therapies presented in this study can provide a new avenue of vast opportunities for durable cancer treatment.

## Methods

### Clinical samples and information

Peripheral blood samples were collected from ten patients enrolled in valemetostat phase I (NCT02732275) or phase II (NCT04102150) trials. No statistical methods were used to determine sample size since this study was exploratory. The availability of patient recruitment thus determined the sample size. All patients with relapsed ATL cases were categorized into clinical subtypes according to Shimoyama’s criteria^38^. This translational study was approved by the Institutional Review Board of the institutes (the University of Tokyo, the University of Ryukyus and Daiichi Sankyo Co., Ltd.). Written informed consents were obtained from all patients. PBMCs from patients with ATL were isolated by Ficoll separation (Ficoll-Paque, GE Healthcare). Clinical information, including abnormal lymphocytes and sIL-2R, was provided by the hospitals. The HTLV-1 proviral load measurement was previously described^14^. In brief, quantitative multiplex real-time PCR was performed with two sets of primers specific for the HTLV-1 provirus and the human gene encoding the RNase P enzyme. The proviral load was expressed as copy numbers per 100 PBMCs, assuming that infected cells had one copy of the integrated HTLV-1 provirus per cell. All clinical samples and data are provided in Supplementary Table 1.

### Cell culture

ATL-derived TL-Om1 cells were provided by an established researcher K. Sugamura. ATN-1 cells were purchased from the RIKEN BRC cell bank (RCB1440). The diffuse large B cell lymphoma cell line WSU-DLCL2 was purchased from DSMZ (ACC 575). HEK293T cells were purchased from the American Type Culture Collection (CRL-3216). HEK293FT cells were purchased from Thermo Fisher Scientific (R70007). These cell lines were verified by each cell bank or established researchers and monitored for cross-contamination. The HTLV-1-infected cell lines had been authenticated based on the provirus integration sites and somatic mutations by panel-based targeted sequencing^18^. Cell-surface expressions of CD4 and CADM1 were validated by flow cytometry. HTLV-1-infected, patient-derived tumour cell lines were established by long-term culture in complete medium RPMI1640 (Invitrogen) with 20% FBS (GIBCO) and 10 ng ml^−1^ IL-2 (Peprotech). Genetic mutations and clonality of the propagating cells were confirmed by targeted sequencing. Commonly misidentified cell lines were not used in this study. The cell lines were also tested for mycoplasma contamination using mycoplasma detection PCR (6601, Takara) and were negative for mycoplasma contamination. Normal (HTLV-1-uninfected) CD4^+^ T cells were obtained from Lonza. All lymphoma cell lines were cultured in RPMI1640 with 10% FBS and antibiotics (Gibco). 293T and 293FT cells were cultured in DMEM (Nissui) with 10% FBS and antibiotics. All cell lines and primary cultures were maintained at 37 °C with 5% CO_2_.

### Flow cytometry

ATL cell populations were obtained using a HAS-flow method as previously described^20^. Single-cell suspensions of lymphocytes were stained with fluorescent-labelled antibodies. An unlabelled CADM1 antibody (CM004-6, clone 3E1) and an isotype control chicken IgY antibody (2:100; PM084) were purchased from MBL. These were biotinylated (primary amine biotinylation) using biotin *N*-hydroxysuccinimide ester (Sigma-Aldrich). Anti-CD14–Pacific orange antibody (MHCD1430, clone TuK4) was purchased from Invitrogen. All other antibodies were obtained from BioLegend. Cells were stained using a combination of anti-CADM1–biotin (1:100; CM004-6, MBL), anti-CD7–APC (5:100; clone CD7-6B7), anti-CD3–APC–Cy7 (5:100; clone SK7), anti-CD4–Pacific blue (5:100; clone RPA-T4) and anti-CD14–Pacific orange (5:100) antibodies. After washing, phycoerythrin-conjugated streptavidin (2:100; SA10041, Thermo Fisher Scientific for phase I study; 1:80; 554061, BD Biosciences for phase II study) was applied. Propidium iodide (Sigma-Aldrich) or 7-AAD (51-68981, BD Biosciences) was added to the samples to stain dead cells immediately before flow cytometry.

For intracellular staining of the H3K27me3, we improved the HAS-Flow method. First, PBMCs (5 × 10^6^) were washed and incubated with Ghost Dyes viability dye (Tonbo Biosciences). Then, the cells were stained using a combination of anti-CD3–APC–Cy7, anti-CD4–Pacific blue, anti-CD7–phycoerythrin–Cy7 (5:100; clone M-T701), anti-CD14–Pacific orange (or BV510 for phase II study), anti-CADM1–biotin and streptavidin–phycoerythrin. The surface-stained cells were then fixed and permeabilized using BD Cytofix fixation buffer (554655, BD Biosciences) and BD Phosflow Perm buffer IV (560746, BD Biosciences) according to the manufacturer’s instructions. After washing, the permeabilized cells were stained with anti-H3K27me3–Alexa Flour 488 (1:200; 5499, clone C36B11, Cell Signaling Technology), anti-histone H3–Alexa Fluor 647 (1:400; 12230, clone D1H2, Cell Signaling Technology), anti-rabbit IgG isotype control–Alexa Flour 488 (1:400; 4340, clone DA1E, Cell Signaling Technology) and anti-rabbit IgG isotype control–Alexa Flour 647 (1:400; 3452, clone DA1E, Cell Signaling Technology). FACSAria II or FACSLyric instrument (BD Biosciences) was used for multicolour flow cytometry and fluorescence-activated cell sorting. The collected data were analysed by FlowJo software (v10.7.1, Tree Star). CD4^+^CADM1^+^CD7^−^ cells and CD4^+^CADM1^−^CD7^+^ cells were analysed as malignant ATL cells and non-malignant cells, respectively. Tumour H3K27me3 levels (mean fluorescence intensity) were calculated by normalization with the data of normal CD4^+^ T cells.

### Targeted deep sequencing

Genomic DNA from enriched cell populations, PBMC, buccal swabs and cell lines were extracted using the QIAamp DNA Blood Mini Kit (Qiagen). Target capture was conducted using the SureSelect Target Enrichment System (Agilent Technologies).

To comprehensively cover genes involved in ATL, 280 human genes were selected, including 50 genes frequently mutated in ATL^16^ and 190 genes frequently mutated in haematological and solid malignancies. Agilent SureDesign web-based application was used for capture bait design as previously described^18^. The sequence data were obtained using the HiSeq2500 or NovaSeq 6000 system (Illumina) with 100-bp paired-end reads. The sequenced data were aligned to the human reference genome hg38 by BWA (v0.7.15) software. The PCR duplicates were removed using Picard (v2.92) and SAMtools (v1.2) software^39^. Matched buccal DNA was used as matched normal controls to call somatic mutations. The somatic mutation candidates were called using MuTect2 from GATK (v4.0.12) software^40^ and annotated with ANNOVAR (v20191024)^41^. Candidate mutations, with (1) 5 or more variant reads in tumour samples, (2) a variant allele frequency in tumour samples 0.01 or more, (3) read depth of 200 or more, and (4) tumour variant with a normal variant ratio of 2 or more, were adopted and further filtered by excluding synonymous SNVs.

### Clonality analysis

The clonality analysis of HTLV-1-infected cells was performed by high-throughput sequencing-based mapping of proviral integration sites^18^. To designate the virus integration sites, sequence reads were aligned to human reference genome hg38 and the virus genome (NC_001436.1) by BWA. Paired-end reads spanning the viral and human genomes and soft-clipped reads (15 bp or more soft-clipped region) were extracted using Perl scripts and then validated by Blastn (v2.6.0+). The clonality was calculated as the population size of each clone by counting the extracted reads at host–provirus junction sites. We used PyClone (v0.13.0)^42^ for the analysis of subclonal population structure and reconstruct hierarchical trees. PyClone is based on a Bayesian clustering method, which uses a Markov chain Monte Carlo-based framework to estimate cellular prevalence values using somatic mutations. The somatic mutation candidates for PyClone were called using MuTect2, with (1) 5 or more variant reads in tumour samples, (2) a variant allele frequency in tumour samples of 0.05 or more, (3) a read depth of 200 or more, and (2) tumour variant with a normal variant ratio of 2 or more. The clonal composition was investigated based on the β-binomial emission model, through which a set of clones with a discrete set of mutations (mutational clusters) were imputed together with their estimated clone size. The process of the clonal evolution was estimated by extrapolation of the estimated clone sizes at all tested time points. The hierarchical trees with imputed mutational subclusters were depicted by ClonEvol (v0.99.11) based on the results of clustering and cellular prevalence from the PyClone model.

### Whole-genome sequencing

For whole-genome sequencing, somatic variant detection was carried out using next-generation sequencing by Azenta Japan Corporation (formerly, Genewiz Japan). In brief, genomic DNA from patient PBMC and matched buccal swabs were quantified and qualified by NanoDrop, Qubit dsDNA HS assay (Thermo Fisher) and agarose gel electrophoresis. Of genomic DNA, 1 µg was sheared into approximately 350 bp in size by an ultrasonicator (Covaris) followed by DNA purification and confirmation of DNA fragment size. Essentially, an entire amount of fragmented genomic DNA was used for library preparation with a PCR-free method (MGIEasy PCR-Free DNA Library Prep Set, MGI tech). The resulting whole-genome sequencing libraries were quantified by Qubit dsDNA HS assay and their fragment size distribution was confirmed by TapeStation D1000 ScreenTape (Agilent). The libraries in the double-stranded DNA form were further processed into single-stranded circular DNA, which is the final form of the MGI sequencing library. The single-stranded circular DNA libraries were quantified by Qubit ssDNA Assay Kit (Thermo Fisher) and used for generating DNA nanoballs by rolling circle replication reaction. DNA nanoballs were then loaded into a flow cell for sequencing on DNBSEQ-G400 platform (MGI tech) with 150 bp paired-end configuration, according to the manufacturer′s instructions, yielding approximately 320 Gb in data amount per library. Sequence data cleaning was performed by the Cutadapt software (v1.9.1)^43^. The Sentieon pipeline (https://www.sentieon.com/products/) was used to call germline single-nucleotide variant/indel and somatic variations. Copy number variation was detected by Control-FREEC^44^.

### RNA-seq

Total RNA of each sample was extracted using TRIzol reagent (Invitrogen) and quantified and qualified by the Agilent 2100 Bioanalyzer (Agilent Technologies), NanoDrop (Thermo Fisher Scientific) and 1% agarose gel. Of total RNA with an RNA integrity number (RIN) value above 7, 20 ng was used following library preparation. The library preparation and sequencing were processed and analysed by Genewiz. The libraries with different indices were multiplexed and loaded on an Illumina HiSeq instrument according to the manufacturer’s instructions (Illumina). Sequencing was carried out using a 2 × 150-bp paired-end configuration; image analysis and base calling were conducted by the HiSeq control software (HCS v2.2.38 or later) plus OLB plus GAPipeline-1.6 (Illumina) on the HiSeq instrument. For quality control, to remove technical sequences, including adapters, PCR primers or fragments thereof, and quality of bases lower than 20, pass filter data of fastq format were processed by Trimmomatic (v0.30) to be high-quality clean data. For mapping, Hisat2 (v2.0.1) was used to index the reference genome sequence. Finally, clean data were aligned to the reference genome via the software Hisat2.

### scATAC-seq

The single-cell RNA-seq library was constructed using the Chromium Controller and Chromium NextGEM Single Cell ATAC Reagent Kits v1.1 (10x Genomics) following the standard manufacturer’s protocols. To collect live cells for ATAC-seq, PBMC cryovials (1–10 × 10^6^ cells per 1 ml of CELLBANKER 1 (Zenoaq resource)) were removed from liquid nitrogen or −80 °C freezer and warmed in a 37 °C water bath. Cells were then pelleted by centrifugation at 500*g* for 5 min and resuspended in PBS. After twice washing with PBS, nuclei isolation was conducted by the 10x Chromium standard protocol. Chilled lysis buffer (100 µl) was added to the pellet, then incubated for 3 min on ice. Chilled wash buffer (1 ml) was added immediately to the lysed cell, followed by two washes. Then, the lysed cell was resuspended in an appropriate volume of chilled diluted nuclei buffer, and 1.6 × 10^4^ nuclei were immediately incubated in a transposition mix to recover 10,000 nuclei. After transposition, the sample was loaded onto the 10x Chromium controller to recover 10,000 nuclei. Gel beads were prepared according to standard manufacturer’s protocols. Oil partitions of single nuclei with oligo-coated gel beads (GEMs) were captured and thermal cycling was performed, resulting in single-stranded DNA tagged with a 10x cell barcode. The library was sequenced using the NovaSeq 6000 system (Illumina) according to the manufacturer’s instructions. For ATAC libraries, sequencing was performed using a 50 × 49-bp paired-end configuration following the manufacturer’s protocol. After sequencing analysis, fastq files were created by the Cell Ranger atac ver2.0.1 mkfastq pipeline (10x Genomics). The obtained fastq files were mapped to the reference genome provided by 10x Genomics (GRCh38). The Cell Ranger atac count pipeline (v2.0.1) was used to perform demultiplexing, aligning reads, filtering, peak calling, clustering and motif activity analyses, using default parameters. The Cell Ranger data were imported into the Loupe Cell Browser software (v6.0.0) for *t*-SNE-based clustering, heat map generation and promoter activity plots.

### scRNA-seq

The scRNA-seq library was constructed using the Chromium Controller and Chromium Single Cell 5′ Reagent Kits and 3′ Reagent Kits v2 (10x Genomics) following the standard manufacturer’s protocols. To collect live cells for scRNA-seq, PBMC cryovials (1–10 × 10^6^ cells per 1 ml of CELLBANKER 1) were removed from liquid nitrogen or −80 °C freezer and warmed in a 37 °C water bath. Cells were then pelleted by centrifugation at 500*g* for 5 min and resuspended in PBS. After twice washing with PBS, cells were then pipetted through a 40-μm filter to remove cell doublets and contamination. Cell viability (more than 60%) was confirmed by trypan blue staining. The collected single-cell suspension from PBMCs (1.6 × 10^4^ live cells per sample) was immediately loaded onto the 10x Chromium Controller to recover thousands of cells from each subpopulation for library preparation and sequencing. Gel beads were prepared according to the standard manufacturer’s protocols. Oil partitions of single cell with GEMs were captured and reverse transcription was performed, resulting in cDNA tagged with a cell barcode and unique molecular index (UMI). The library was sequenced using the NovaSeq 6000 system (Illumina) according to the manufacturer’s instructions. Sequencing was carried out using a 1 × 91–98-bp single-end configuration (default setting), which is sufficient to align confidentially to the transcriptome. After sequencing analysis, fastq files were created by the Cell Ranger ver3.1.0 mkfastq pipeline (10x Genomics). The obtained fastq files were mapped to the reference genome provided by 10x Genomics (GRCh38). The Cell Ranger count pipeline (v3.1.0) was used to perform demultiplexing, aligning reads, filtering, clustering and gene expression analyses, using default parameters. In brief, after read trimming, Cell Ranger used an aligner called STAR, which performs splicing-aware alignment of reads to the genome. Cell Ranger further aligned exonic and intronic confidently mapped reads to annotated transcripts by examining their compatibility with the transcriptome. Only uniquely mapping exonic reads were carried forward to UMI counting. After the UMI filtering steps with default parameters and expected cell counts, each observed barcode, UMI and gene combination was recorded as a UMI count in the feature–barcode matrix. The workflow also performed an improved calling cell barcodes algorithm, identified the primary mode of high RNA content cells and also captured low RNA content cells.

After data processing, we recovered quality-assured data for secondary analysis of gene expression. To correct batch effects between time points, we used a Cell Ranger merge algorithm. To regress out the cell–cell variation in gene expression driven by batch and cluster data with corrected data in different time points, we used a standard Seurat v3 integration workflow with functions FindIntegrationAnchors() and IntegrateData(). The Cell Ranger data or batch-corrected data were imported into Loupe Cell Browser software (v6.0.0) for *t*-SNE-based clustering, heat map generation and gene expression distribution plots.

### Single-cell multiome analysis

The single-cell multiome (scMultiome) libraries were constructed by using Chromium Controller and 10x Genomics Chromium Next GEM Single Cell Multiome ATAC plus Gene Expression following the standard manufacturer’s protocols (CG000365 Rev C, CG000338 Rev F, 10x Genomics). The libraries were sequenced using the NovaSeq 6000 system (Illumina) according to the manufacturer’s instructions. For ATAC libraries, sequencing was performed using a 50 × 49-bp paired-end configuration. RNA library sequencing was performed using a 28 × 91-bp paired-end configuration. The scMultiome dataset was first processed using Cell Ranger ARC v2.0.0 (Cell Ranger ARC, 10x Genomics). BCL files were converted into fastq using the command cellranger_ark mkfastq with default parameter. The fastq files were then processed by cellranger_ark count and merged by cellranger-arc aggr. To remove batch effect, the scMultiome RNA dataset was processed by Seurat (v4.3.0)^24^ reciprocal principal component analysis (clustering parameters principal component analysis dimensions 1–30, resolution 0.5). The scMultiome ATAC dataset was recounted by Signac (v1.9.0)^45^ using the merged peak bed files and processed by Harmony (v0.1.1)^46^.

### Single-cell mutation identification and analysis

RNA variants from scRNA-seq data were validated from curated BAM files based on the results of Cell Ranger. For each cell barcode in the filtered Cell Ranger barcode list, and each somatic variant in the targeted sequencing data, variant bases were identified. Only reads with a Chromium cellular barcode tag and a Chromium molecular barcode tag were included. We then obtained the cell-associated tag for downstream analysis of UMIs. Chromium cellular barcode tags with the variant reads extracted by SAMtools were defined as at least one mutant read detected and mapped on each *t*-SNE projection using Loupe Cell Browser software. Almost variants were validated by manual review to identify mutant cells accurately. One-sided Fisher’s exact tests were used to identify cell clusters that were enriched for somatic mutations (*P* < 0.05).

### Virus reads and host–virus chimeric reads from single-cell data

For detection of virus reads from scATAC-seq and scRNA-seq data, we processed Cell Ranger GRCh38-aligned sequence data. No-map and soft-clipped reads (more than 20 bp soft-clipped) were extracted using Python scripts. The pass-filter data of fastq format were processed to remove adopter and polyA sequences. The high-quality clean data were then aligned to the human reference genome (hg38) and virus genome (NC_001436.1) via the software STAR. For detection of cells expressing virus genes, Chromium cellular barcode tags with virus reads were defined as at least one virus read detected. Almost virus-aligned reads were derived from the antisense strand. Both host-aligned and virus-aligned soft-clipped reads were extracted as host–virus chimeric reads. Genomic breakpoints of chimeric reads were analysed from supplementarily mapped data from STAR alignment to link the clone-specific chimeric reads with the viral integration sites identified in the corresponding clones. The extracted Chromium cellular barcode tags with virus antisense reads or clone-specific host–virus chimeric reads were mapped on *t*-SNE projection using the Loupe Cell Browser. One-sided Fisher’s exact tests were used to identify cell clusters that were enriched for virus reads (*P* < 0.05).

### Cluster assignment and single-cell data analysis

Promoter activity (promoter sum) and expression patterns of *CD4*, *CADM1* and *CD7* were used and overlaid on the *t*-SNE to identify ATL tumour clusters using the Loupe Cell Browser. Chromium cellular barcodes with HTLV-1-derived antisense transcripts (scRNA-seq) and proviral DNA reads (scATAC-seq) were overlaid on the *t*-SNE. The HTLV-1-derived reads served for inference of infected cells (*P* < 0.05). Infected clone-specific host–virus chimeric reads were significantly enriched in each cluster (*P* < 0.05). To detect the mutation-harbouring clones estimated by PyClone, RNA variants from scRNA-seq data were validated from curated BAM files based on the results of Cell Ranger. Chromium cellular barcode tags with variant reads were defined as at least one mutant read detected and mapped on each *t*-SNE projection (*P* < 0.05). log_2_ Fold change and median-normalized average values of assigned clusters were obtained via the Loupe Cell Browser and used in the following analysis of differentially expressed genes within each cluster. Manual clustering based on expression patterns was curated by original Python scripts or polygonal selection tool (Loupe Cell Browser interface).

### ChIP–seq

Tumour cells (1 × 10^7^) sorted by surface markers (CD4^+^CADM1^+^CD7^−^) or normal CD4^+^ T cells from HTLV-1-negative healthy donors were fixed by adding 1/10 volume of freshly prepared formaldehyde solution (11% (v/v) formaldehyde, 100 mM NaCl, 1 mM EDTA (pH 8.0) and 50 mM HEPES (pH 7.9)) to the existing media or PBS and incubated for 15 min at room temperature. Fixation was stopped by adding 1/20 volume of a 1.25 M glycine solution and incubating for 5 min at room temperature. Subsequently, cells were collected and washed twice with chilled PBS with 0.5% (v/v) Igepal. The cell pellet was snap-frozen on dry ice. Further processing and ChIP experiments including chromatin extraction, fragmentation, antibody precipitation and library preparation were performed at Active Motif using validated antibodies to H3K27me3 (39155, polyclonal, Active Motif), H3K27ac (39133, polyclonal, Active Motif) and SUZ12 (39357, polyclonal, Active Motif).

Illumina sequencing libraries were prepared from the ChIP and input DNAs by the standard consecutive enzymatic steps of end-polishing, dA-addition and adaptor ligation. After a final PCR amplification step, the resulting DNA libraries were quantified and sequenced on NextSeq 500 from Illumina (75-nt reads, single end). Reads were aligned to the human genome (hg38) using the BWA algorithm (v0.7.12). Duplicate reads were removed, and only uniquely mapped reads (mapping quality ≥ 25) were used for further analysis. Alignments were extended in silico at their 3′ ends to a length of 200 bp, which is the average genomic fragment length in the size-selected library, and assigned to 32-nt bins along the genome. The resulting histograms (genomic ‘signal maps’) were stored in bigWig files. Peak call for H3K27me3 and H3K9me3 were performed using the SICER algorithm (v1.1) with a cut-off *P* = 10^−10^. Peak call for H3K27ac was performed using the MACS algorithm (v2.1.0) with a cut-off *P* = 10^−7^. Peaks that were on the ENCODE blacklist of known false ChIP–seq peaks were removed. Signal maps and peak locations were used as input data to the Active Motifs proprietary analysis program, which creates Excel tables containing detailed information on sample comparison, peak metrics, peak locations and gene annotations. EaSeq software (v1.111)^47^ was also used to calculate each peak value and create heat maps. For the TSS plot, the ChIP–seq dataset was normalized by input data and visualized by Deeptools (v3.3.1)^48^.

### DNA methylation profiling

For DNA methylation profiling, genomic DNA was extracted from enriched tumour cell populations (CD4^+^CADM1^+^CD7^−^) and cell lines using the QIAamp DNA Blood Mini Kit (Qiagen). DNA methylation levels were analysed using the Infinium MethylationEPIC BeadChip (more than 850,000 probes) (Illumina). Quality testing of the double-stranded DNA was performed by measuring absorbance with NanoDrop2000 and fluorescence with Qubit (Thermo Fisher Scientific), followed by quality testing by agarose gel electrophoresis. Genomic DNA was used for bead array analysis by iScan (Illumina) according to the Infinium HD methylation protocol guide, manual protocol (15019519 v01). Bisulfite conversion, hybridization and further data processing were performed at Takara Bio. In brief, bisulfite conversion of 250 ng of genomic DNA was performed using the EZ DNA Methylation Kit (Zymo Research). The bisulfite-converted DNA was alkaline denatured and subjected to enzymatic whole-genome amplification. The amplified genomic DNA was fragmented by enzyme, purified by isopropanol precipitation and resuspended in buffer. The resuspended DNA was heat denatured and applied to the Infinium MethylationEPIC BeadChip for hybridization at 48 °C in an oven for approximately 23 h. After hybridization, the BeadChip was washed with buffer, and a single nucleotide labelled at the probe end was incorporated by a single-nucleotide elongation reaction. The hybridized genomic DNA was then denatured, removed and stained with a fluorescent dye-labelled antibody against the incorporated labelled nucleotide. The stained BeadChip was washed, coated, dried and then fluorescence images were acquired using iScan. Normalization by background subtraction and internal controls was performed using GenomeStudio (V2011.1) or Methylation Module (v1.9.0) to analyse the acquired fluorescence image data. Each CpG site was annotated by distance from the TSS of the genes (hg38). Only CpG sites within ±5 kb of the TSS were used for further integrative analyses. The β-value was used as the methylation level (%), and probes that fluctuated more than 10% were defined as differentially methylated sites. BigWig files were created using the Enhancer Linking by Methylation/Expression Relationship (ELMER) package with the function createBigWigDNAmetArray().

For whole-genome DNA methylation analyses of patient specimens and established resistant models, we performed EM-seq^49^. The libraries of EM-seq were prepared from 50 ng of DNA using the NEBNext Enzymatic Methyl-seq Kit (New England BioLabs). Paired-end sequencing of 150 bp was performed using NovaSeq 6000. The EM-seq dataset was adapter-timmed by Trim Galore v0.6.7 with the default parameters. The trimmed reads were aligned to hg38 using Bismark (v0.22.3)^50^. PCR duplicates were removed using deduplicate_bismark with default parameter. The methylation information was extracted with a bismark_methylation_extractor. The methylation information had a filtered depth of more than five. Differential methylated regions were extracted using metilene (v0.2-8)^51^ (*P* < 0.05). All methylated CpG sites were also analysed at single-nucleotide resolution from the EM-seq data. The methylation information bedGraphs of bismark outputs were converted to BigWig by bedGraphToBigWig and visualized by Integrative Genomics Viewer. Methylation levels of target genes were calculated by Deeptools v3.3.1 and visualized by Deeptools plotProfile.

### Bioinformatic analysis

The Integrative Genomics Viewer tool^52^ was used for visualizing and interpreting the results of DNA-seq, RNA-seq, ChIP–seq and DNA methylation data. For differentially expressed gene analysis, HTSeq (v0.6.1) estimated gene and convert read counts to transcripts per million from the paired-end clean data. Selected genes were subjected to the hierarchical clustering analysis using the iDEP.91 pipeline that contains the DESeq2 package^53^. Gene set enrichment analysis^54^ was performed using GSEA software (v4.1.0) (http://www.broadinstitute.org/gsea) with 1,000 permutations. Gene sets used in this study were selected from the MSigDB hallmark gene sets (http://www.broadinstitute.org/gsea/msigdb/collections.jsp). Significantly enriched gene sets were evaluated by normalized enrichment score (NES) and nominal *P* value (*P* < 0.001). Gene ontology analysis was performed by DAVID Bioinformatics Resources (https://david.ncifcrf.gov/).

### Data visualization

Box plots, beeswarm plots, violin plots, hierarchical clustering and correlation matrix were analysed and visualized by using R (v3.2.3). Box plots are defined as follows: the middle line corresponds to the median; the lower and upper hinges correspond to first and third quartiles; the upper whisker extends from the hinge to the largest value no further than 1.5 times the IQR from the hinge (where the IQR is the interquartile range or distance between the first and third quartiles); and the lower whisker extends from the hinge to the smallest value at most 1.5 times the IQR of the hinge. All data points are overlaid on the box plot.

### Molecular dynamics simulation

The valemetostat-bound PRC2 structure was modelled as previously described^11^. Amino acid residue numbers for EED in the PRC2 model were renumbered based on UniProt O75530 isoform 1 (identifier: O75530-1). Binding free-energy changes (ΔΔ*G*s) of valemetostat to PRC2 single-point mutants (EZH2(Y111S/Y111C/Y111H/Y111N), EZH2(Y661N) and EED(H213R)) relative to wild-type PRC2 were predicted by the free-energy perturbation (FEP) method^55,56^ using FEP protein mutation for ligand selectivity (Schrödinger release 2021-3: FEP+, Schrödinger, 2021) with default settings and the OPLS4 force field^57^. The value of ΔΔ*G* for EZH2(Y111H) was defined as the mean of ΔΔ*G*s for EZH2(Y111) mutated to histidine neutral tautomers, Nδ-protonated (Hid) and Nε-protonated (Hie), respectively. Hydrogen bonds, hydrophobic interactions, ionic interactions, and water bridges between valemetostat and wild-type or mutant PRC2s were examined throughout 5-ns molecular dynamics simulations using edge analysis of FEP protein mutation analysis (Schrödinger release 2021-3: FEP+, Schrödinger) to elucidate the effect of these mutations in PRC2 on valemetostat binding. Structural model figures were generated using PyMOL (v2.4.0, Schrödinger). Relative affinities of valemetostat to PRC2 mutants were predicted by FEP simulations and calculated as wild-type dissociation constant (*K*_d_)/(wild-type or mutant *K*_d_) = exp(−ΔΔ*G*/*RT*), where *R* is the ideal gas constant (1.987 cal K^–1^ mol^–1^) and *T* is the absolute temperature (298.15 K).

### Evaluation of PRC2 mutants

*EZH2* and *EED* cDNAs were subcloned into the pME-FLAG vector. Point mutagenesis for generating resistant mutants was accomplished with the PrimeSTAR Mutagenesis Basal Kit (Takara) and specific primer sets (Supplementary Table 6). The generated mutant cDNAs were confirmed by Sanger sequencing. Transient transfection of FLAG-tagged cDNA in 293T cells was performed by Lipofectamine 2000 (Thermo Fisher). At 24 h after transfection, the medium was replaced with fresh medium supplemented with valemetostat and cultured for 5 days. The subsequent H3K27me3 level was evaluated by immunoblotting with primary antibodies (anti-H3K27me3 (1:1,000; 07-449, Merck/Millipore), anti-histone H3 total (1:1,000; ab10799, Abcam) and anti-FLAG M2 (1:1,000; F1804, Sigma)).

### Generation and evaluation of resistant cell models

ATL cell lines were cultured in growth media supplemented with 10 nM of valemetostat for 2 months. Inhibitor-resistant outgrowth was observed at 100 nM. For knockdown of *TET2*, *DNMT3A*, *DNMT3B* and PRC2 genes, a replication-defective, self-inactivating lentivirus vector (CS-H1-Venus-IRES-Bsd) was used (Riken, BRC). We designed three shRNA sequences (Supplementary Table 6) and cloned them into CS-RfA-EVBsd via pENTR4-H1. For stable expression of wild-type and mutant EZH2 and EED in lymphoma cells, FLAG-tagged cDNAs were subcloned into lentivirus vector CSII-EF-MCS-IRES2-Venus (Riken). For stable expression of DNMT in lymphoma cells, haemagglutinin-tagged *DNMT3A* and *DNMT3B* cDNA were subcloned into the lentivirus vector pHIV-dTomato (Addgene #21374). DNMT3A(E629stop), which lacks the C-terminal enzymatic domain, was also generated for a negative control. A *TET2*-encoded lentivirus vector was purchased from VectorBuilder (pLV-Puro-EF1A-hTET2). The established viral vectors were co-transfected with the packaging plasmid (pCAG-HIVgp) and the VSV-G-expressing and Rev-expressing plasmid (pCMV-VSV-G-RSV-Rev) into 293FT cells. High-titre viral solutions were prepared by centrifugation-based concentration and used for transduction into cell lines. The infection was attained by the spinoculation method and then cultured in an appropriate condition for 5–7 days. Blasticidin (10 μg ml^−1^) was used to select the transduced population. Expression of fluorescent proteins (Venus and dTomato) was confirmed by flow-cytometory using FACSCalibur or FACSymphony A1 (BD Biosciences), or by automated cell counter using Countess 3 FL (Thermo Fisher Scientific). Expression levels of DNMT3A and DNMT3B were evaluated by immunoblotting with primary antibodies as follows: anti-DNMT3A (1:1,000; 3598, Cell Signaling Technology) and anti-DNMT3B (1:1,000; 57868, Cell Signaling Technology). Alternatively, knockdown and gene induction efficiencies were evaluated by qRT–PCR with specific primer sets (Supplementary Table 6). For evaluation of the anti-growth activity of valemetostat, lymphoma cell models (2 × 10^5^) were plated in 12-well flat bottom plates with optimized media with 10% FBS and simultaneously treated with indicated doses of valemetostat solution in DMSO for 14 days. The cells were maintained by passage into fresh media every 3–4 days. The cell numbers were evaluated by Cell Counting Kit-8 (WST-8 assay, Dojindo) following the manufacturer’s protocol. Valemetostat used in this study was synthesized in-house.

### Methylation-specific PCR

Genomic DNA (1 μg) was converted with sodium bisulfite using the EpiTect Bisulfite Kit (Qiagen). The converted DNA (200 ng) was amplified by KOD -multi & Epi- DNA polymerase (Toyobo) with methylated or unmethylated specific primer pairs for CpG islands within *CDKN1A*, *CDKN1C* and *BCL2L11* promoters (Supplementary Table 6). The PCR products were analysed by 2% agarose gel stained with ethidium bromide and visualized under UV light.

### Resistant outgrowth assay

To evaluate the ability of PRC2 mutants, DNMT3A, DNMT3B and *TET2*-targeting shRNA to acquire resistance to valemetostat, a resistance outgrowth assay^27,28^ in the presence of valemetostat was performed. Lymphoma cells expressing each gene or negative control cells were cultured with valemetostat at IC_90_ or higher for 1 week. Then, 10 cells per well were spread on 96-well plates and cultured in the presence of valemetostat with successive passages for more than 1 month. The cumulative cell count in each well was then measured using the WST-8 assay to determine the percentage of wells that outgrowth in the presence of valemetostat. Growth suppression resistance of the randomly collected outgrowth clones was evaluated. Gene expression levels were also evaluated by qRT–PCR. To evaluate the effect of DNA methylation, additional cultures were maintained for 1 week in the presence of a low concentration (10 nM) of 5-aza-2′-deoxycytidine (decitabine from Merck) and subsequent gene expression and cell counts were evaluated.

### Evaluation of translation activity

For the 5′ UTR reporter assay, 5′ UTR sequences of EZH1, EZH2, SUZ12 and EED were amplified from the human genomic DNA region with specific primers (Supplementary Table 6) and inserted into the BamHI site upstream of the start codon of pMIR-REPORT (Promega). The orientation of the inserted 5′ UTR was confirmed by Sanger sequencing. The luciferase activities were quantified by the Dual-Luciferase Reporter Assay System (Promega) 2 days after transfection.

The CRISPR–Cas9-based ‘double nicking’^33^ was applied to delete a part of the endogenous EZH2 5′ UTR. To minimize nonspecific effects of guide RNA (gRNA) and to induce some length of deletion on the UTR, a double-nicking strategy with Cas9 nickase (Cas D10A) and double gRNA was used to introduce double-stand breaks at the target site. The gRNAs were designed using the CRISPR gRNA Design tool (DNA2.0) from the target sequence within EZH2 5′ UTR (cggtgggactcagaaggcagtggagccccggcggcggcggcggcggcgcgcgg; PAM sequences at both ends). The gRNA sequences are provided in Supplementary Table 6. An all-in-one vector (All-in-One Nickase Ninja vector, pD1421-AD), which can express two gRNAs and Cas9, was constructed and introduced into 293T and TL-Om1 cells using Lipofectamine 2000. After 48 h, GFP-positive cells were sorted. The 5′ UTR sequences of ten clones in TA-cloning were analysed by Sanger sequencing to confirm that deletion was occurring. The secondary structure and free-energy change of the 5′ UTR sequence were predicted using the mfold tool^58^. The CRISPR-transduced cells were used as bulk culture and characterized.

For the RIP assay, cells (2 × 10^7^) were washed with PBS and lysed with 1 ml of RNA lysis buffer (25 mM Tris-HCl (pH 7.4), 150 mM KCl, 5 mM EDTA, 0.5% NP-40, 1 mM dithiothreitol, protease inhibitor cocktail and 100 U ml^−1^ RNase inhibitor (Takara)). After incubation on ice for 20 min, the cells were centrifuged at 4 °C at 14,000 rpm for 20 min to obtain cell lysate. Dynabeads protein G (Invitrogen) was added to the lysate and rotated at 4 °C for 15 min to remove proteins nonspecifically bound to the beads. For antibody-bound beads, Dynabeads protein G was washed and inculcated with anti-eIF3D (A301-758A, Bethyl Laboratories), anti-eIF3A (2013, Cell Signaling Technology) or control IgG (2729, Cell Signaling Technology) antibodies for 10 min. The prepared antibody-binding beads were added to the cell lysate and slowly rotated at 4 °C for 1 h. After washing five times with RNA lysis buffer, beads were mixed with 1 ml of TRIzol. The collected RNA was subjected to reverse-transcriptase reaction using ReverTra Ace qRT–PCR Master Mix (Toyobo) with the manufacturer’s protocol. Random primer-based synthesized cDNA was analysed by quantitative PCR using a real-time PCR system (Thermal cycler Dice, Takara). *EZH2* and *JUN* mRNA levels were quantified using gene-specific primers (Supplementary Table 6).

For evaluation of the translation activity of *EZH2* mRNA, the amount of mRNA in the ribosomal and polysomal fractions was quantified using sucrose density gradient centrifugation. The 15–40% sucrose density gradient solution (containing 10 mM Tris-HCl (pH 7.5), 140 mM NaCl, 5 mM MgCl_2_, 1 mM dithiothreitol and 100 μg ml^−1^ cycloheximide) was prepared in a centrifuge tube (Beckman Coulter). Cells (2 × 10^7^) were washed with PBS containing 100 μg ml^−1^ cycloheximide for 5 min, then lysed with polysome lysis buffer (10 mM Tris-HCl (pH 7.5), 140 mM NaCl, 1.5 mM MgCl_2_, 0.5% NP-40, 0.5% deoxycholate, 2 mM dithiothreitol, 100 U ml^−1^ RNase inhibitor, 100 μg ml^−1^ cycloheximide and protease inhibitor) and then placed on top of the density gradient solution. The lysates were then centrifuged at 38,000 rpm for 2 h at 4 °C using SW41Ti rotor (Beckman Coulter). Twenty-four fractions were collected in 500-µl portions, and the absorbance was measured at 254 nm using a NanoDrop. RNA was extracted from each fraction using ISOGEN-LS (Nippon Gene), and the *EZH2* mRNA level in each fraction was quantified by qRT–PCR.

For protein analysis of the subpopulations with different H3K27me3, total proteins from the fixed cells were extracted for immunoblotting according to a previous study^32^. The fixed proteins could be liberated from formaldehyde crosslinking in the presence of high heat, 500 mM Tris and 2% SDS. In brief, H3K27me3-depleted and relatively H3K27me3-high tumour cells were sorted from the two post-dose blood samples and then sonicated in 200 µl of modified fixed tissue lysis buffer (500 mM Tris-HCl (pH 7.4), 100 mM NaCl, 25 mM EDTA, 1% (v/v) Triton X-100, 1% (v/v) IGEPAL, 2% (w/v) SDS and protease inhibitor cocktail). Homogenates were incubated at 90 °C for 120 min, followed by centrifugation at 4 °C. Protein levels of the collected supernatants were analysed by immunoblotting.

For eIF3D knockdown, the lentivirus vector CS-H1-Venus-IRES-Bsd was used with two shRNA sequences (Supplementary Table 6). Protein levels of eIF3D, PRC2 factors and H3K27me3 were analysed by immunoblotting with primary antibodies, as follows; anti-EZH1 (1:1,000; 42088, Cell Signaling Technology), anti-EZH2 (1:1,000; 3147, Cell Signaling Technology), anti-SUZ12 (1:1,000; 3737, Cell Signaling Technology), anti-EED (1:1,000; 85322, Cell Signaling Technology), anti-eIF3D (1:1,000; A301-758A, Bethyl Laboratories), anti-H3K27me3 (1:1,000; 07-449, Merck/Millipore), anti-COX4 (1:1,000; 4850, Cell Signaling Technology), anti-TFAM (1:1,000; 8076, Cell Signaling Technology), anti-JUN (1:1,000; 9165, Cell Signaling Technology) and anti-β-actin (1:1,000; sc-69879, Santa Cruz). Alkaline phosphatase-conjugated anti-mouse (1:2,000; S3721, Promega) and anti-rabbit (1:2,000; S3731, Promega) secondary antibodies and BCIP/NBT substrate (S3771, Promega) were used for detection.

### Statistics and reproducibility

All bar and line graphs that summarize multiple datasets show mean values. The middle lines within box plots indicate median values. Significant differences in gene expression and other biological assays between the two groups were analysed by a two-sided Student’s *t*-test. Adjustments were not made for multiple comparisons. Correlations between two groups were analysed by a two-sided Pearson’s correlation coefficients, and probabilities of overlap between gene sets were statistically tested. For electrophoresis of samples with cell lines, representative data from two to three independent repeat experiments are shown. Because experiments on multiple outgrowth clones are verified for reproducibility by examining multiple samples of interest, electrophoresis was performed only once. In addition, electrophoresis experiments with multiple independent patient samples were performed once.

### Reporting summary

Further information on research design is available in the Nature Portfolio Reporting Summary linked to this article.

## Online content

Any methods, additional references, Nature Portfolio reporting summaries, source data, extended data, supplementary information, acknowledgements, peer review information; details of author contributions and competing interests; and statements of data and code availability are available at 10.1038/s41586-024-07103-x.

### Supplementary information

Supplementary Fig. 1 Gel raw images.

Reporting Summary

Supplementary Tables This file contains Supplementary Tables 1–6.

### Source data

Source Data Fig. 1

Source Data Fig. 2

Source Data Fig. 3

Source Data Fig. 4

Source Data Fig. 5

Source Data Extended Data Fig. 1

Source Data Extended Data Fig. 2

Source Data Extended Data Fig. 3

Source Data Extended Data Fig. 5

Source Data Extended Data Fig. 6

Source Data Extended Data Fig. 8

Source Data Extended Data Fig. 9

Source Data Extended Data Fig. 10

## Data Availability

All sequencing data (fastq format), including Target-seq, RNA-seq, scRNA-seq, scATAC-seq and ChIP–seq, have been deposited in the National Bioscience Database Center Human Database under the accession number JGAS000553. A previous scRNA-seq dataset (JGAS000301) was used for validation. The reference human genome hg38 was downloaded from the UCSC Genome Browser. For gel source data, see Supplementary Fig. 1. Source data are provided with this paper.
