# Supplementary material for: Mechanisms of action and resistance in histone methylation-targeted therapy
Source: Nature. 2024 Feb 21;627(8002):221–8. doi: 10.1038/s41586-024-07103-x (PMC10917674; doi:10.1038/s41586-024-07103-x)

nature portfolio

https://doi.org/10.1038/s41586-024-07103-x

Supplementary information

Mechanisms of action and resistance in histone methylation-targeted therapy

In the format provided by the authors and unedited

Raw data for Fig 5e
Dotted areas denote blots' images included in the manuscript

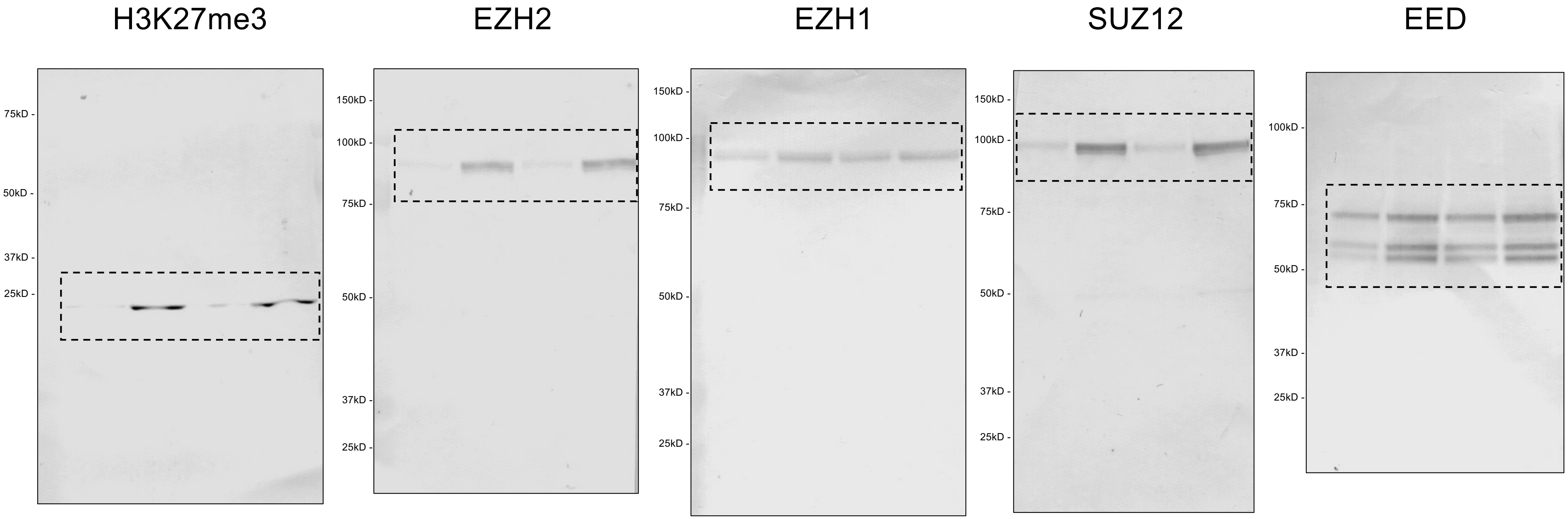

Raw data for Fig 5e
Dotted areas denote blots' images included in the manuscript

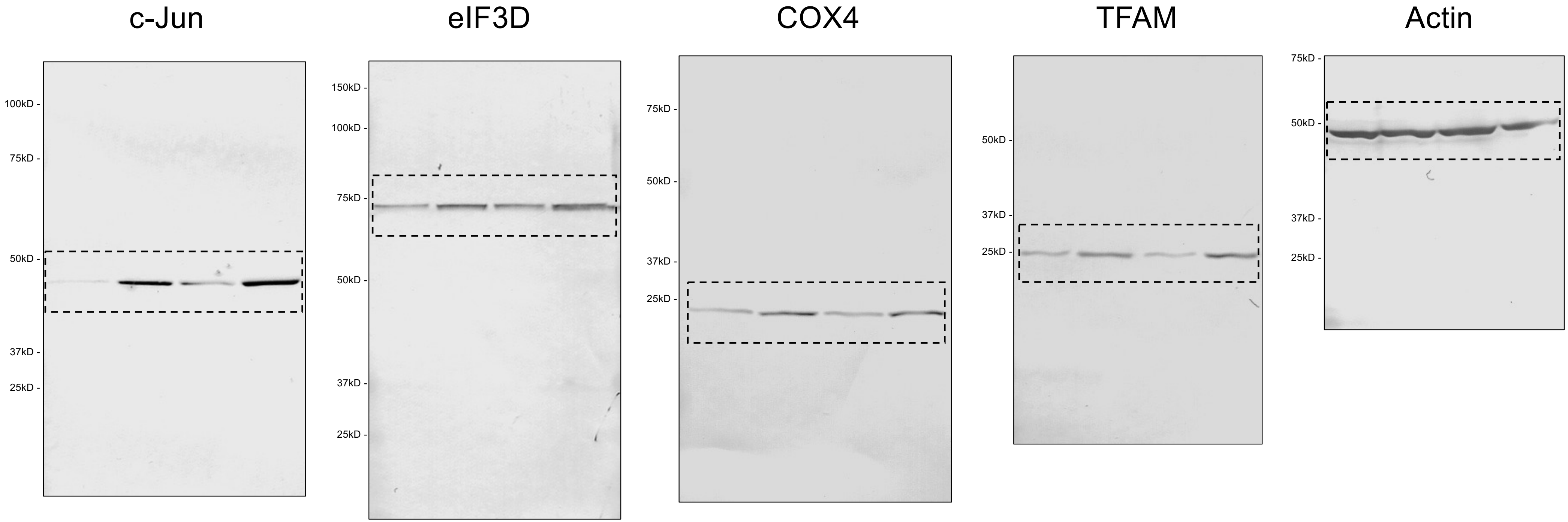

Raw data for Fig 5g
Dotted areas denote blots' images included in the manuscript

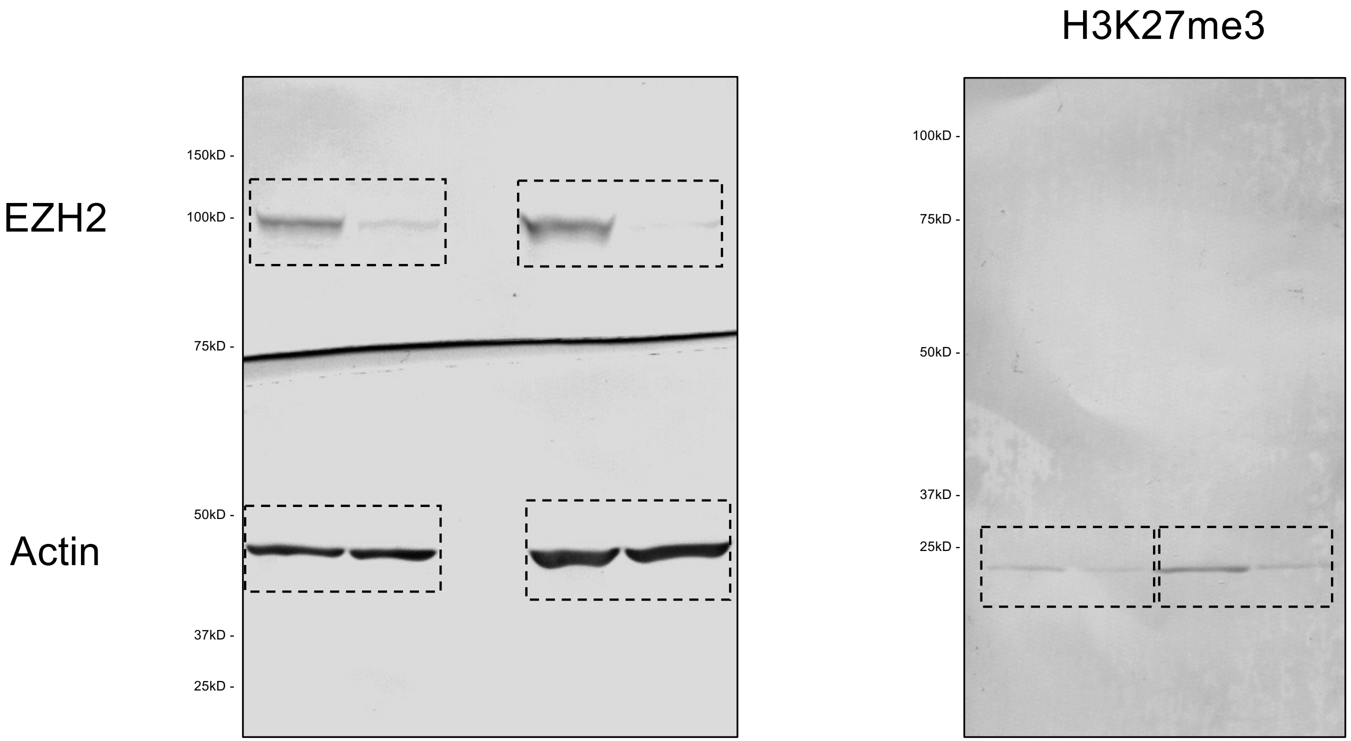

Raw data for Extended Data Fig 5e
Dotted areas denote blots' images included in the manuscript

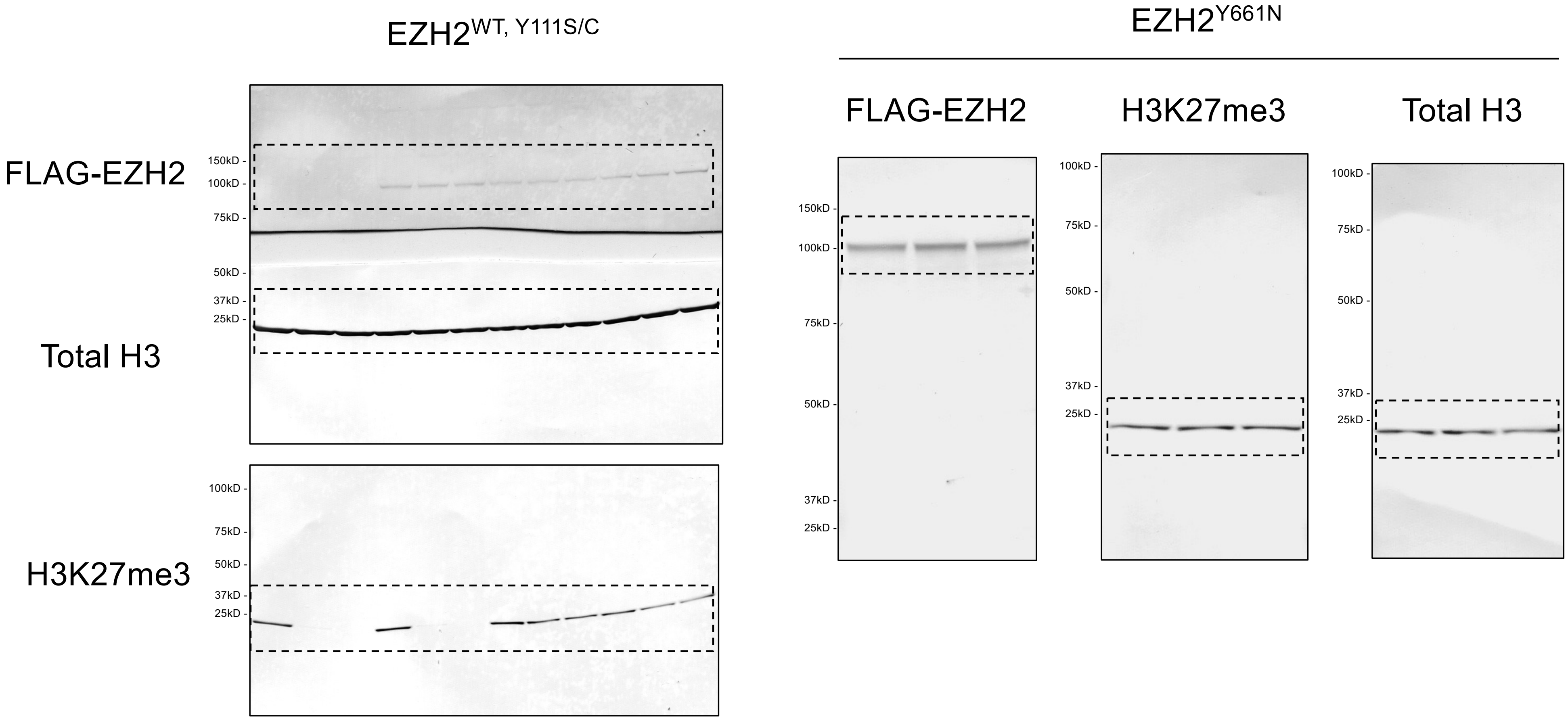

Raw data for Extended Data Fig 5e
Dotted areas denote blots' images included in the manuscript

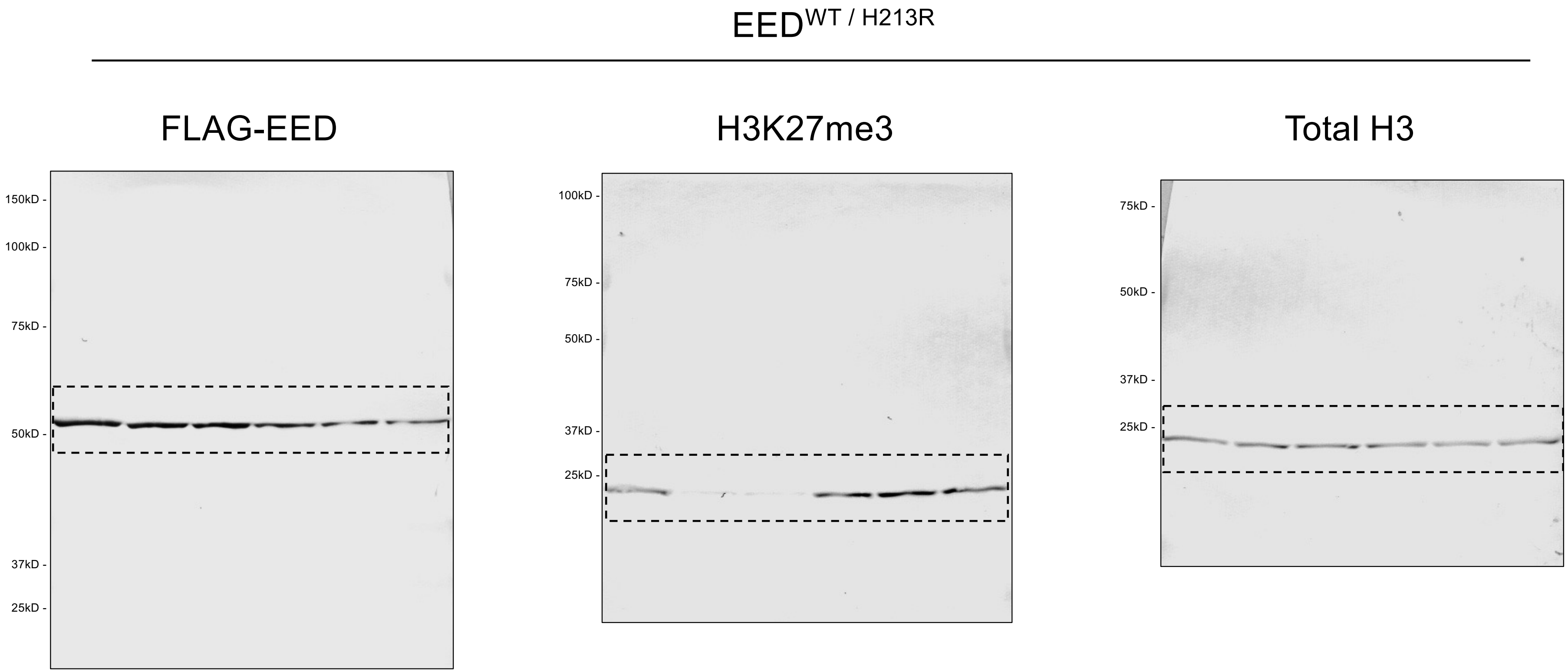

Raw data for Extended Data Fig 8h
Dotted areas denote blots' images included in the manuscript

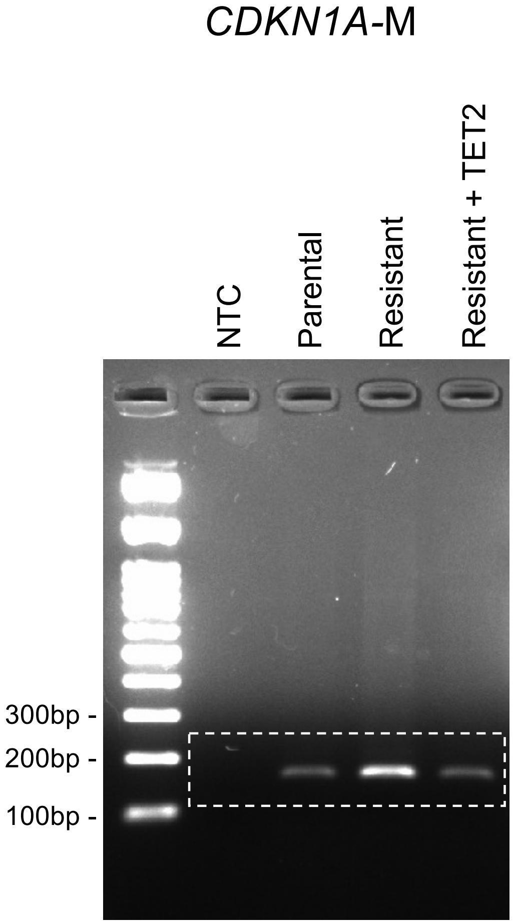

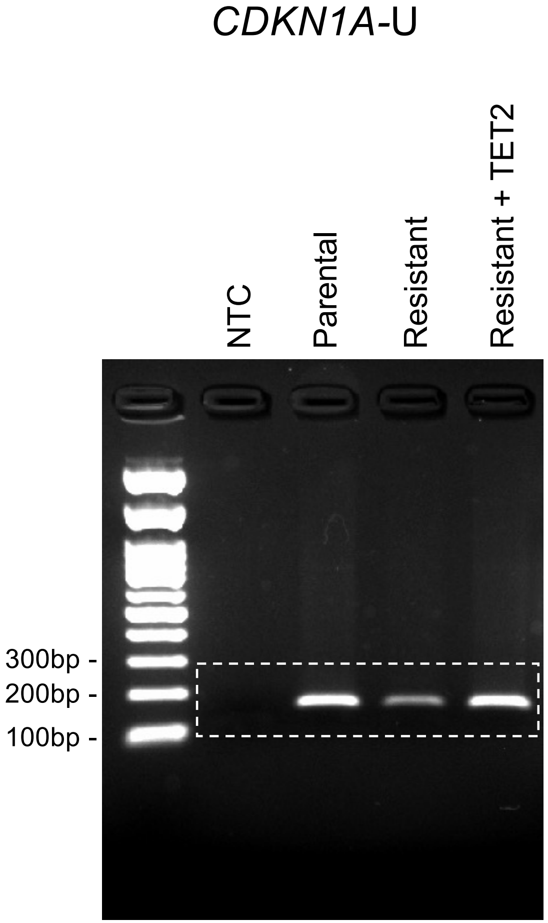

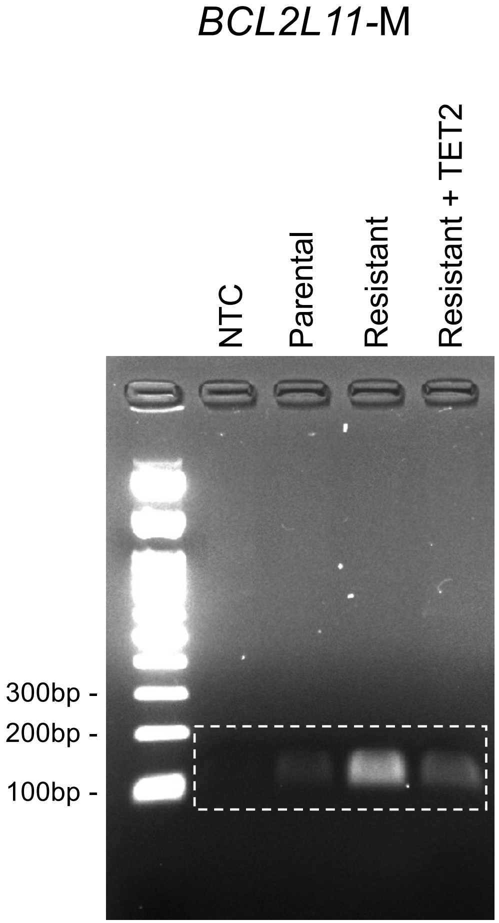

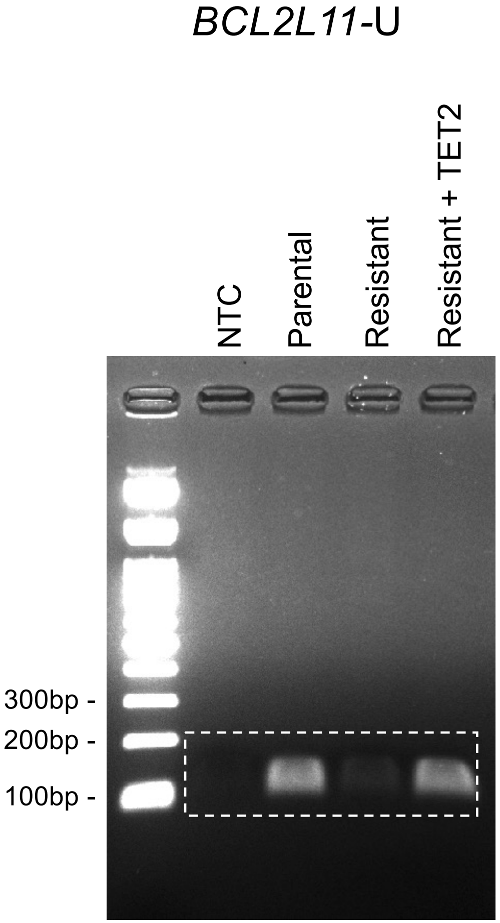

Raw data for Extended Data Fig 8k
Dotted areas denote blots’ images included in the manuscript

shTET2#1 clone

H3K27me3

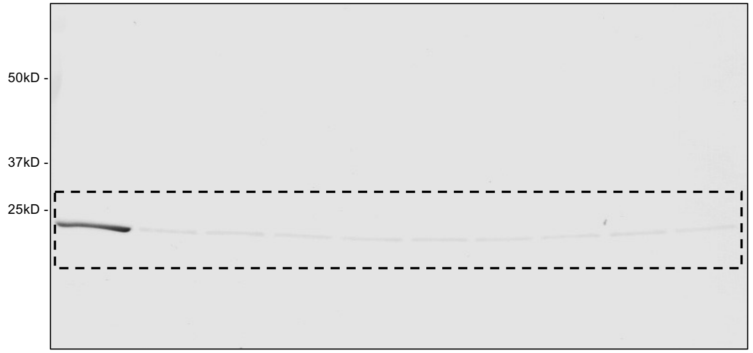

Total H3

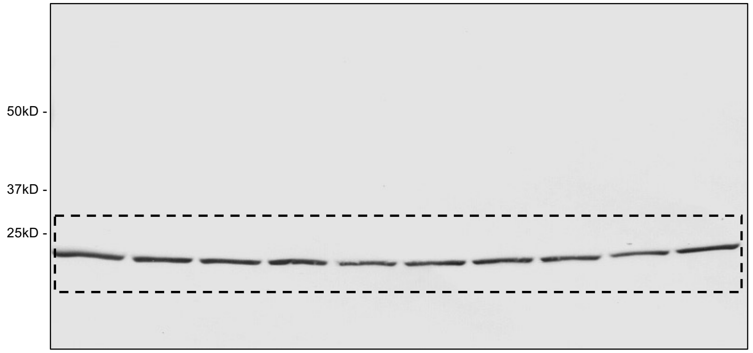

shTET2#2 clone

H3K27me3

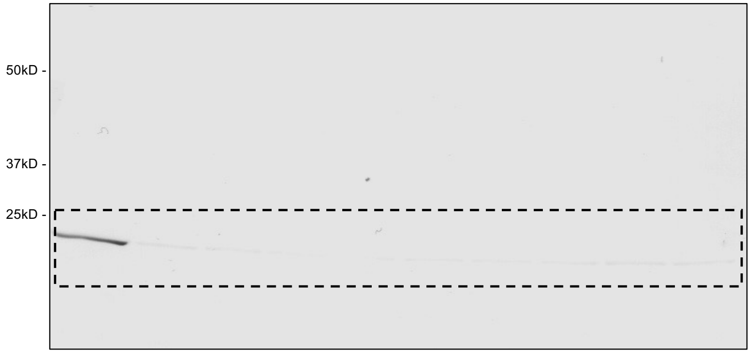

Total H3

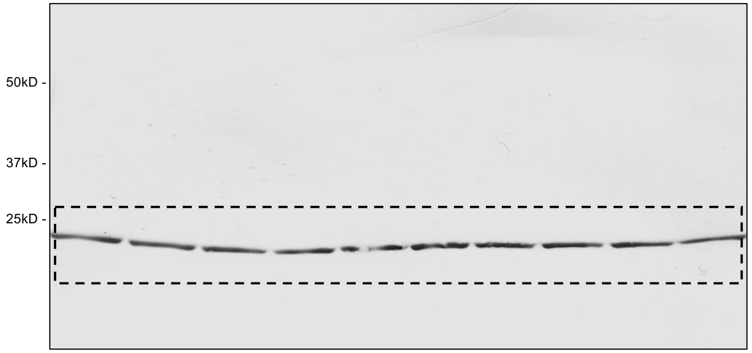

Raw data for Extended Data Fig 8m
Dotted areas denote blots' images included in the manuscript

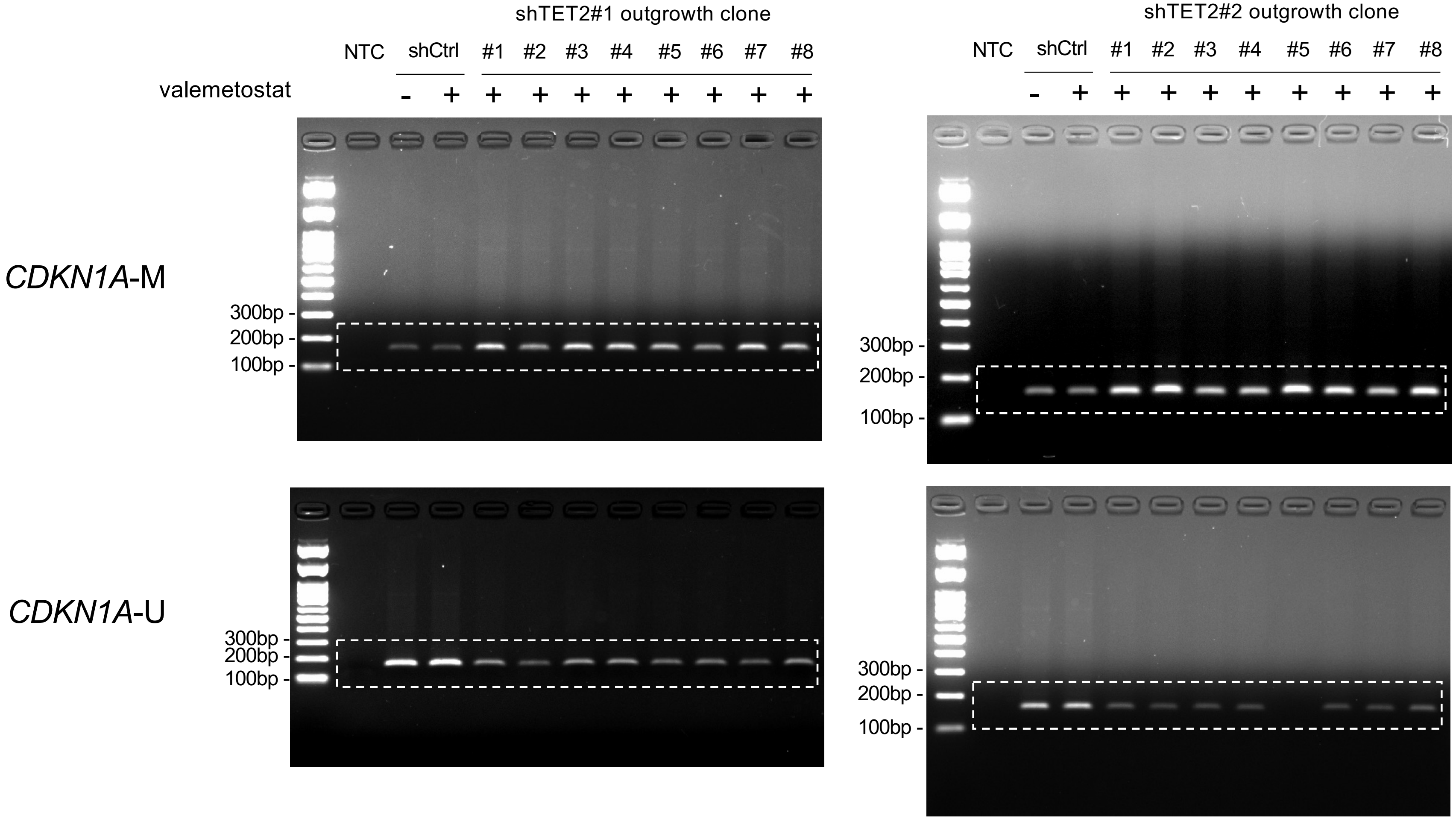

Raw data for Extended Data Fig 8m
Dotted areas denote blots' images included in the manuscript

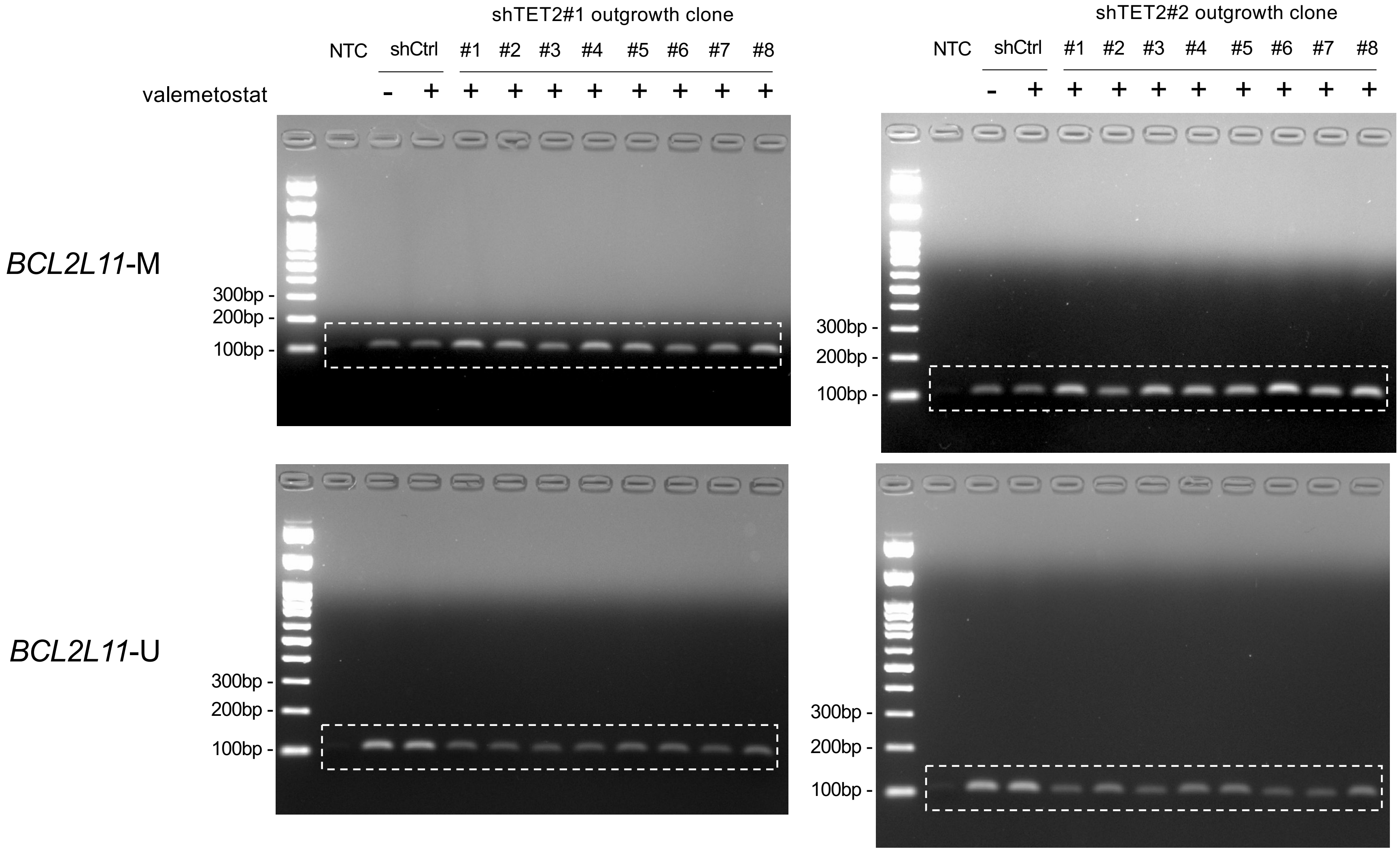

Raw data for Extended Data Fig 9e
Dotted areas denote blots' images included in the manuscript

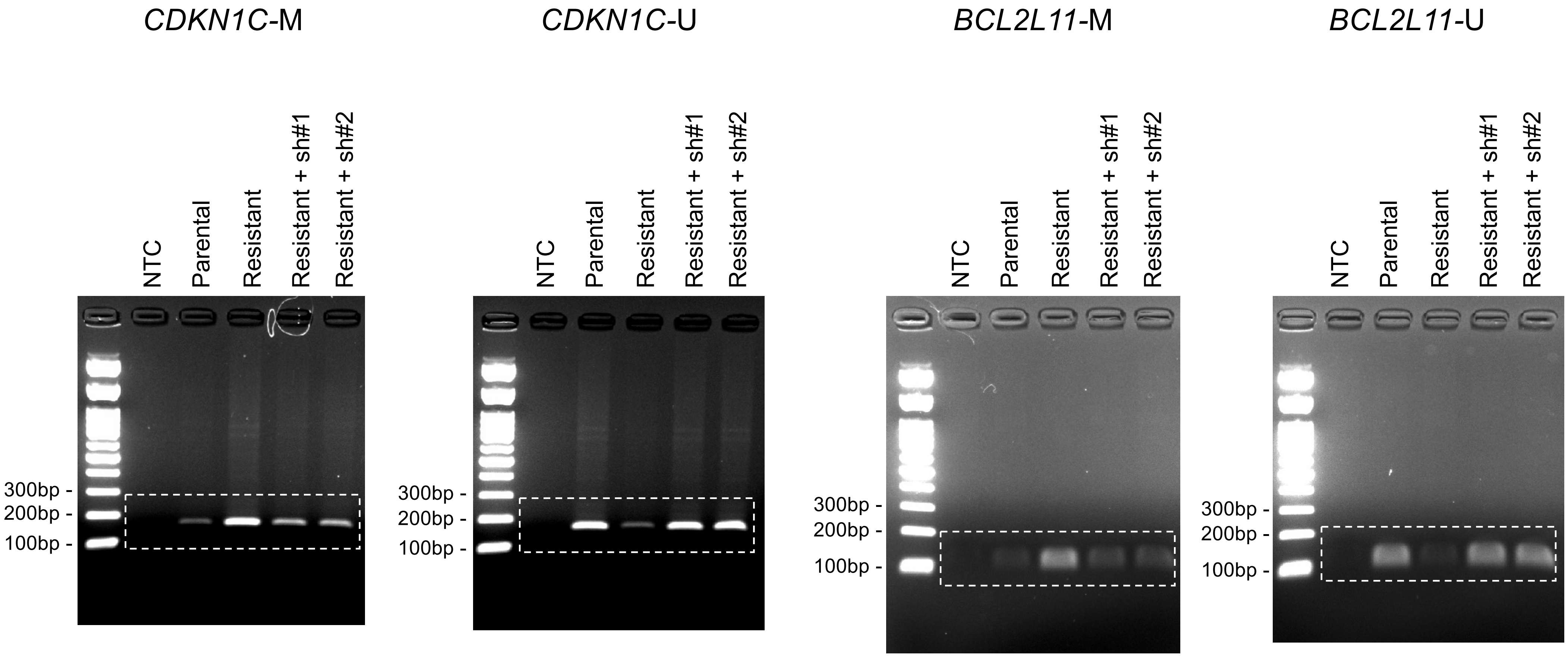

Raw data for Extended Data Fig 9f
Dotted areas denote blots' images included in the manuscript

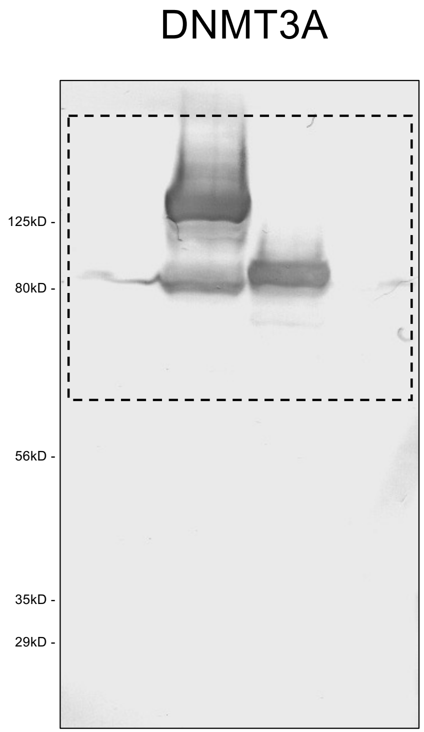

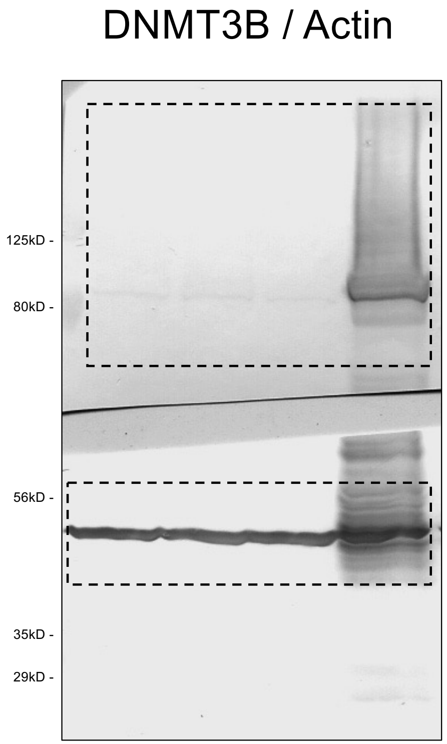

Raw data for Extended Data Fig 10l
Dotted areas denote blots’ images included in the manuscript

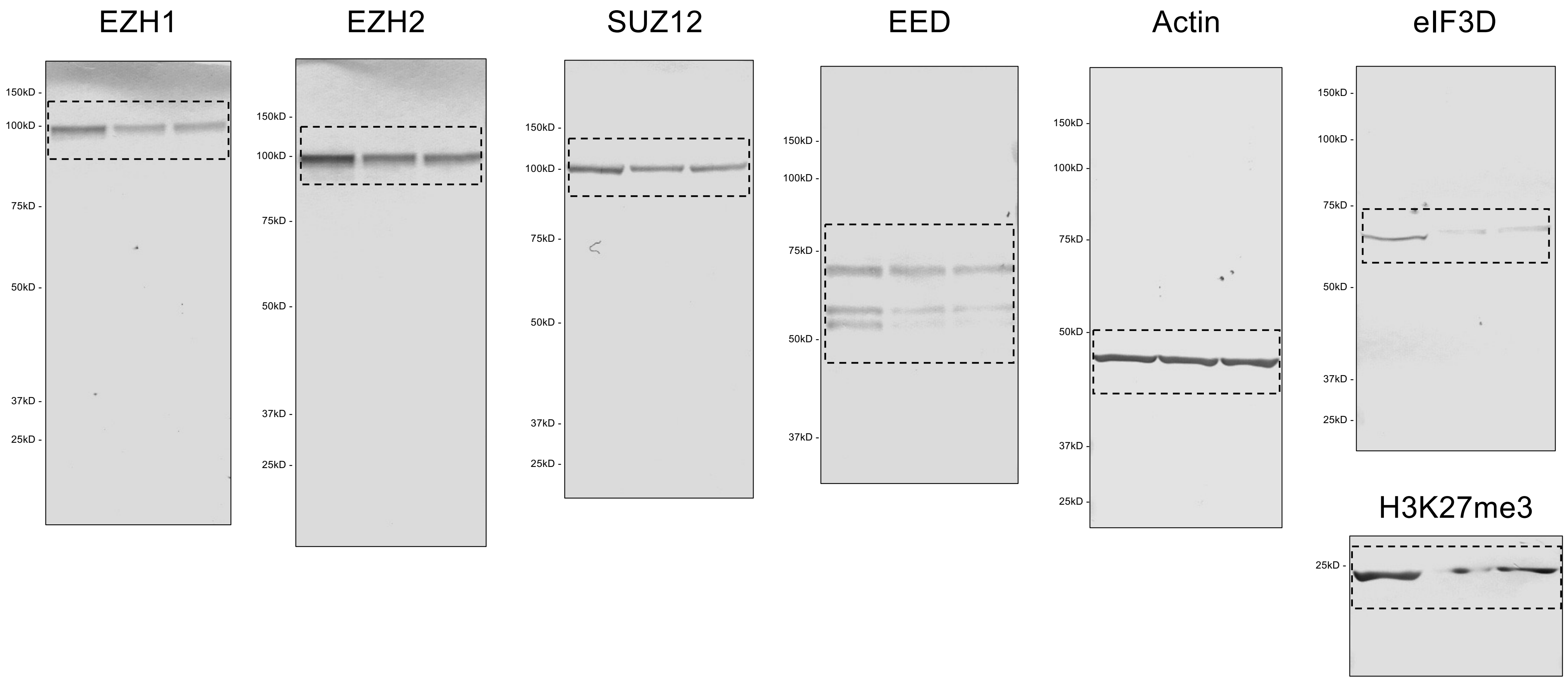

Supplement: Supplementary file 1 — Gel raw images. [file 41586_2024_7103_MOESM1_ESM.pdf]
