# Supplementary material for: Mechanisms of action and resistance in histone methylation-targeted therapy
Source: Nature. 2024 Feb 21;627(8002):221–8. doi: 10.1038/s41586-024-07103-x (PMC10917674; doi:10.1038/s41586-024-07103-x)
Supplement: Supplementary file 2 — Reporting Summary [file 41586_2024_7103_MOESM2_ESM.pdf]

nature portfolio

Corresponding author(s): Makoto Yamagishi
Kaoru Uchimaru
Yutaka Suzuki

Last updated by author(s): Dec 12, 2023

Reporting Summary

Nature Portfolio wishes to improve the reproducibility of the work that we publish. This form provides structure for consistency and transparency in reporting. For further information on Nature Portfolio policies, see our Editorial Policies and the Editorial Policy Checklist.

Statistics

For all statistical analyses, confirm that the following items are present in the figure legend, table legend, main text, or Methods section.

n/a	Confirmed	
☐	☒	The exact sample size (*n*) for each experimental group/condition, given as a discrete number and unit of measurement
☐	☒	A statement on whether measurements were taken from distinct samples or whether the same sample was measured repeatedly
☐	☒	The statistical test(s) used AND whether they are one- or two-sided<br>*Only common tests should be described solely by name; describe more complex techniques in the Methods section.*
☒	☐	A description of all covariates tested
☒	☐	A description of any assumptions or corrections, such as tests of normality and adjustment for multiple comparisons
☐	☒	A full description of the statistical parameters including central tendency (e.g. means) or other basic estimates (e.g. regression coefficient) AND variation (e.g. standard deviation) or associated estimates of uncertainty (e.g. confidence intervals)
☐	☒	For null hypothesis testing, the test statistic (e.g. *F*, *t*, *r*) with confidence intervals, effect sizes, degrees of freedom and *P* value noted<br>*Give P values as exact values whenever suitable.*
☒	☐	For Bayesian analysis, information on the choice of priors and Markov chain Monte Carlo settings
☒	☐	For hierarchical and complex designs, identification of the appropriate level for tests and full reporting of outcomes
☐	☒	Estimates of effect sizes (e.g. Cohen's *d*, Pearson's *r*), indicating how they were calculated

Our web collection on statistics for biologists contains articles on many of the points above.

Software and code

Policy information about availability of computer code

Data collection	For Target-seq, RNA-seq, scATAC-seq, scRNA-seq, scMultiome analysis, and EM-seq, sequencing and base calling were conducted by HiSeq2500 system (HiSeq Control Software v2.2.38 or later, Illumina) or Novaseq 6000 system (Novaseq Control Software v1.6.0 or v1.7.5, Illumina) according to the manufacturer's instruction. Single-cell sequencing was performed on 10x Chromium controller (10x Genomics). DNBSEQ-G400 platform (MGI tech) was used for WGS according to the manufacturer's instructions. FACSAria II or FACSLyric instrument (BD Biosciences) was used for multicolor flow cytometry and fluorescence-activated cell sorting.
Data analysis	Targeted deep sequencing.<br>The sequenced data were aligned to the human reference genome hg38 by BWA (v0.7.15) software. The polymerase chain reaction (PCR) duplicates were removed using Picard (v2.92) and SAMtools (v1.2) software. Matched buccal DNA was used as matched normal controls to call somatic mutations. The somatic mutation candidates were called using MuTect2 from GATK (v4.0.12) software and annotated with ANNOVAR (v20191024). Candidate mutations with (i) ≥ 5 variant reads in tumor samples, (ii) a VAF in tumor samples ≥ 0.01, (iii) read depth ≥ 200, and (iv) tumor variant: normal variant ratio ≥ 2, were adopted and further filtered by excluding synonymous SNVs.<br><br>Clonality analysis.<br>The clonality analysis of HTLV-1-infected cells was performed by high-throughput sequencing based mapping of proviral integration sites. To designate the virus integration sites, sequence reads were aligned to human reference genome hg38 and virus genome (NC_001436.1) by BWA. Paired-end reads spanning the viral and human genomes and soft-clipped reads (> 15 bp soft-clipped region) were extracted using Perl scripts and then validated by Blastn (v2.6.0+). The clonality was calculated as the population size of each clone by counting the extracted reads at host-provirus junction sites. We used PyClone (v0.13.0) for the analysis of subclonal population structure and reconstruct hierarchical trees. PyClone is based on a Bayesian clustering method, which uses a Markov chain Monte Carlo-based framework to estimate cellular

prevalence values using somatic mutations. The somatic mutation candidates for PyClone were called using MuTect2, with (i) ≥ 5 variant reads in tumor samples, (ii) a VAF in tumor samples ≥ 0.05, (iii) read depth ≥ 200, and (iv) tumor variant: normal variant ratio ≥ 2. The clonal composition was investigated based on the beta binomial emission model, through which a set of clones with a discrete set of mutations (mutational clusters) were imputed together with their estimated clone size. The process of the clonal evolution was estimated by extrapolation of the estimated clone sizes at all tested timepoints. The hierarchical trees with imputed mutational subclusters were depicted by ClonEvol (v0.99.11) based on the results of clustering and cellular prevalence from the PyClone model.

Whole-genome sequencing.
Sequence data cleaning was performed by Cutadapt software (version 1.9.1). The Sentieon pipeline was used to call germline SNV/InDel and somatic variations. CNV was detected by Control-FREEC.

RNA sequencing.
For quality control, to remove technical sequences, including adapters, PCR primers, or fragments thereof, and quality of bases lower than 20, pass filter data of fastq format were processed by Trimmomatic (v0.30) to be high-quality clean data. For mapping, Hisat2 (v2.0.1) was used to index the reference genome sequence. Finally, clean data were aligned to the reference genome via software Hisat2.

scATAC sequencing.
After sequencing analysis, fastq files were created by the Cell Ranger atac ver2.0.1 mkfastq pipeline (10x Genomics). The obtained fastq files were mapped to the reference genome provided by 10x Genomics (GRCh38). Cell Ranger atac count pipeline (v2.0.1) was used to perform demultiplexing, aligning reads, filtering, peak calling, clustering, and motif activity analyses, using default parameters. The Cell Ranger data were imported into Loupe Cell Browser Software (v6.0.0) for t-distributed stochastic neighbor embedding (t-SNE) based clustering, heatmap generation, and promoter activity plots.

scRNA sequencing.
After sequencing analysis, fastq files were created by the Cell Ranger ver3.1.0 mkfastq pipeline (10x Genomics). The obtained fastq files were mapped to the reference genome provided by 10x Genomics (GRCh38). Cell Ranger count pipeline (v3.1.0) was used to perform demultiplexing, aligning reads, filtering, clustering, and gene expression analyses, using default parameters. Briefly, after read trimming, Cell Ranger used an aligner called STAR, which performs splicing-aware alignment of reads to the genome. Cell Ranger further aligned exonic and intronic confidently mapped reads to annotated transcripts by examining their compatibility with the transcriptome. Only uniquely mapping exonic reads were carried forward to UMI counting. After the UMI filtering steps with default parameters and expected cell counts, each observed barcode, UMI, gene combination was recorded as a UMI count in the feature-barcode matrix. The workflow also performed an improved Calling Cell Barcodes algorithm, identified the primary mode of high RNA content cells and also captured low RNA content cells. After data processing, we recovered quality-assured data for secondary analysis of gene expression. To correct batch effects between timepoints, we used a Cell Ranger merge algorithm. To regress out the cell–cell variation in gene expression driven by batch and cluster data with corrected data in different timepoints, we used standard Seurat v3 integration workflow with functions FindIntegrationAnchors() and IntegrateData(). The Cell Ranger data or batch-corrected data were imported into Loupe Cell Browser Software (v6.0.0) for t-SNE-based clustering, heatmap generation, and gene expression distribution plots.

Single-cell multiome (scMultiome) analysis.
scMultiome dataset was first processed using Cell Ranger ARC ver 2.0.0 (Cell Ranger ARC, 10x Genomics). BCL files were converted into fastq using the command cellranger_ark mkfastq with default parameter. The fastq files were then processed by cellranger_ark count and merged by cellranger-arc aggr. To remove batch effect, scMultiome RNA dataset was processed by Seurat (v4.3.0) reciprocal PCA (clustering parameters PCA dimensions 1~30, resolution 0.5). scMultiome ATAC dataset was recounted by Signac (v1.9.0) using the merged peak bed files and processed by Harmony (v0.1.1).

Single-cell mutation identification and analysis.
RNA variants from scRNA-seq data were validated from curated BAM files based on the results of Cell Ranger. For each cell barcode in the filtered Cell Ranger barcode list, and each somatic variant in the targeted sequencing data, variant bases were identified. Only reads with a Chromium Cellular Barcode (CB) tag and a Chromium Molecular Barcode (UB) tag were included. We then obtained the cell-associated tag for downstream analysis of UMIs. CB tags with the variant reads extracted by SAMtools were defined as at least one mutant read detected and mapped on each t-SNE projection using Loupe Cell Browser Software. Almost variants were validated by manual review to identify mutant cells accurately. One-sided Fisher exact tests were used to identify cell clusters that were enriched for somatic mutations ($P < 0.05$).

Virus reads and host-virus chimeric reads from single-cell data.
For detection of virus reads from scATAC-seq and scRNA-seq data, we processed Cell Ranger GRCh38-aligned sequence data. No-map and soft-clipped reads (> 20 bp soft-clipped) were extracted using Python scripts. The pass filter data of fastq format were processed to remove adopter and polyA sequences. The high-quality clean data were then aligned to the human reference genome (hg38) and virus genome (NC_001436.1) via software STAR. For detection of cells expressing virus genes, CB tags with virus reads were defined as at least one virus read detected. Almost virus-aligned reads were derived from the antisense strand. Both host- and virus-aligned soft-clipped reads were extracted as host-virus chimeric reads. Genomic breakpoints of chimeric reads were analyzed from supplementarily mapped data from STAR alignment to link the clone-specific chimeric reads with the viral integration sites identified in the corresponding clones. The extracted CB tags with virus antisense reads or clone-specific host-virus chimeric reads were mapped on t-SNE projection using Loupe Cell Browser. One-sided Fisher exact tests were used to identify cell clusters that were enriched for virus reads ($P < 0.05$).

Cluster assignment and single-cell data analysis.
Promoter activity (Promoter Sum) and expression patterns of CD4, CADM1, and CD7 were used and overlaid on the t-SNE to identify ATL tumor clusters using the Loupe Cell Browser. CBs with HTLV-1-derived antisense transcripts (scRNA-seq) and proviral DNA reads (scATAC-seq) were overlaid on the t-SNE. The HTLV-1-derived reads served for inference of infected cells ($P < 0.05$). Infected clone-specific host-virus chimeric reads were significantly enriched in each cluster ($P < 0.05$). To detect the mutation-harboring clones estimated by PyClone, RNA variants from scRNA-seq data were validated from curated BAM files based on the results of Cell Ranger. CB tags with variant reads were defined as at least one mutant read detected and mapped on each t-SNE projection ($P < 0.05$). Log2 fold-change and median-normalized average values of assigned clusters were obtained via Loupe Cell Browser and used in the following analysis of differentially expressed genes within each cluster. Manual clustering based on expression patterns was curated by original Python scripts or polygonal selection tool (Loupe Cell Browser interface).

ChIP sequencing.

Reads were aligned to the human genome (hg38) using the BWA algorithm (v0.7.12). Duplicate reads were removed, and only uniquely mapped reads (mapping quality >= 25) were used for further analysis. Alignments were extended in silico at their 3′-ends to a length of 200 bp, which is the average genomic fragment length in the size-selected library, and assigned to 32-nt bins along the genome. The resulting histograms (genomic "signal maps") were stored in bigWig files. Peak call for H3K27me3 were performed using the SICER algorithm (v1.1) with a cutoff of P value = 10–10. Peak call for H3K27ac were performed using the MACS algorithm (v2.1.0) with a cutoff of P value = 10–7. Peaks that were on the ENCODE blacklist of known false ChIP-seq peaks were removed. Signal maps and peak locations were used as input data to Active Motifs proprietary analysis program, which creates Excel tables containing detailed information on sample comparison, peak metrics, peak locations and gene annotations. EaSeq software (v1.111) was also used to calculate each peak value and create heatmaps.

DNA methylation profiling.
Normalization by Background Subtraction and Internal Controls was performed using GenomeStudio (V2011.1) / Methylation Module (v1.9.0) to analyze the acquired fluorescence image data. Each CpG site was annotated by distance from the TSS of the genes (hg38). Only CpG sites within ±5 Kbp of the TSS were used for further integrative analyses. The [illegible]-value was used as the methylation level (%), and probes that fluctuated more than 10% were defined as differentially methylated sites. Bigwig files were created using the Enhancer Linking by Methylation/Expression Relationship (ELMER) package with the function createBigWigDNAmetArray().
For whole genome DNA methylation analysis, EM-seq dataset was adapter-timmed by Trim Galore ver0.6.7 with the default parameters. The trimmed reads were aligned to hg38 using Bismark (v0.22.3). PCR duplicates were removed using deduplicate_bismark with default parameter. The methylation information was extracted with a bismark_methylation_extractor. The methylation information was filtered depth > 5. Differential methylated regions (DMRs) were extracted using metilene (v0.2-8) ($P < 0.05$). The methylation information bedGraphs of bismark outputs were converted to bigwig by bedGraphToBigWig and visualized by IGV. Methylation levels of target genes were calculated by Deeptools ver3.3.1 and visualized by Deeptools plotProfile.

Bioinformatic analysis and statistics.
Integrative Genomics Viewer (IGV) tool was used for visualizing and interpreting the results of DNA-seq, RNA-seq, ChIP-seq, and DNA methylation data. For differentially expressed gene analysis, HTSeq (v0.6.1) estimated gene and convert read counts to transcripts per million (TPM) from the pair-end clean data. Selected genes were subjected to the hierarchical clustering analysis using iDEP.91 pipeline that contains DESeq2 package. Gene set enrichment analysis (GSEA) was performed using GSEA software (v4.1.0) (http://www.broadinstitute.org/gsea) with 1,000 permutations. Gene sets used in this study were selected from the MSigDB hallmark gene sets (http://www.broadinstitute.org/gsea/msigdb/collections.jsp). Significantly enriched gene sets were evaluated by normalized enrichment score (NES) and nominal P value ($P < 0.001$). Gene Ontology analysis was performed by DAVID Bioinformatics Resources (https://david.ncifcrf.gov/). Significant differences in gene expression and other biological assays between the two groups were analyzed by a two-sided Student's t-test. Adjustments were not made for multiple comparisons. Correlations between two groups were analyzed by a two-sided Pearson's correlation coefficients and probabilities of overlap between gene sets were statistically tested.

Flow cytometry.
The collected flow cytometry data were analyzed by FlowJo software (v10.7.1, Tree Star).

For manuscripts utilizing custom algorithms or software that are central to the research but not yet described in published literature, software must be made available to editors and reviewers. We strongly encourage code deposition in a community repository (e.g. GitHub). See the Nature Portfolio guidelines for submitting code & software for further information.

Data

Policy information about availability of data

All manuscripts must include a data availability statement. This statement should provide the following information, where applicable:

- Accession codes, unique identifiers, or web links for publicly available datasets
- A description of any restrictions on data availability
- For clinical datasets or third party data, please ensure that the statement adheres to our policy

All sequencing data (fastq format), including Target-seq, RNA-seq, scRNA-seq, scATAC-seq, and ChIP-seq, have been deposited in the National Bioscience Database Center (NBDC) Human Database under an accession number JGAS000553 (https://humandbs.biosciencedbc.jp/en/hum0252-v2). Previous scRNA-seq dataset (JGAS000301) was used for validation. The reference human genome hg38 was downloaded from UCSC Genome Browser. For gel raw data, see Supplementary Figure 1. Other source data are provided with this paper.

Human research participants

Policy information about studies involving human research participants and Sex and Gender in Research.

Reporting on sex and gender	The sex of the clinical specimens available is provided in Supplementary Table 1.
Population characteristics	Peripheral blood samples were collected from patients enrolled in valemetostat phase 1 (NCT02732275) and phase 2 (NCT04102150) trials in Japan. Covariate-relevant population characteristics of the human research participants are provided in Supplementary Table 1.
Recruitment	Peripheral blood samples were collected from ten patients enrolled in valemetostat phase 1 (NCT02732275) or phase 2 (NCT04102150) trials. All patients with relapsed or refractory ATL cases were categorized into clinical subtypes according to Shimoyama's criteria.

Ethics oversight	This translational study was approved by the Institutional Review Board of the institutes (the University of Tokyo, the University of Ryukyus, and Daiichi Sankyo Co., Ltd.). Written informed consents were obtained from all patients.

Note that full information on the approval of the study protocol must also be provided in the manuscript.

Field-specific reporting

Please select the one below that is the best fit for your research. If you are not sure, read the appropriate sections before making your selection.

☒ Life sciences ☐ Behavioural & social sciences ☐ Ecological, evolutionary & environmental sciences

For a reference copy of the document with all sections, see nature.com/documents/nr-reporting-summary-flat.pdf

Life sciences study design

All studies must disclose on these points even when the disclosure is negative.

Sample size	No statistical methods were used to determine sample size since this is an exploratory study. Sample size was thus determined by the availability of patient recruitment. We enrolled individuals who provided consent for our study during the enrollment period. List of used clinical samples are provided in Supplementary Table 1.
Data exclusions	All data was included in the current study.
Replication	The experimental findings were reliably and independently reproduced. The replication numbers were described in the corresponding figure legends.
Randomization	Not applicable since this is a case-series study which was therefore not planned to detect any difference in effects between the cohorts with and without intervention. No animal studies were conducted in this study, all experiments were in vitro except those using clinical specimens, and no randomization was required. Thus, randomization was not relevant to the study design.
Blinding	Blinding was not relevant to our study with clinical samples because it was essential to understand underlying confounding variables in our associations, such as clinical subtype, sex, etc. No animal studies were conducted in this study, all experiments were in vitro except those using clinical specimens, and no blinding was required.

Reporting for specific materials, systems and methods

We require information from authors about some types of materials, experimental systems and methods used in many studies. Here, indicate whether each material, system or method listed is relevant to your study. If you are not sure if a list item applies to your research, read the appropriate section before selecting a response.

Materials & experimental systems

n/a	Involved in the study	
☐	☒	Antibodies
☐	☒	Eukaryotic cell lines
☒	☐	Palaeontology and archaeology
☒	☐	Animals and other organisms
☐	☒	Clinical data
☒	☐	Dual use research of concern

Methods

n/a	Involved in the study	
☐	☒	ChIP-seq
☐	☒	Flow cytometry
☒	☐	MRI-based neuroimaging

Antibodies

Antibodies used	Flow cytometry.<br>An unlabeled CADM1 antibody (CM004-6, clone 3E1) and an isotype control chicken immunoglobulin Y (IgY) antibody (PM084) were purchased from MBL. These were biotinylated (primary amine biotinylation) using biotin N-hydroxysuccinimide ester (Sigma-Aldrich). Anti-CD14-Pacific Orange antibody (MHCD1430, clone TuK4) was purchased from Invitrogen. All other antibodies were obtained from Biolegend. Cells were stained using a combination of anti-CADM1-biotin (MBL, CM004-6), anti-CD7-APC (clone CD7-6B7), anti-CD3-APC-Cy7 (clone SK7), anti-CD4-Pacific Blue (clone RPA-T4), and anti-CD14-Pacific Orange (MHCD1430, clone TuK4) antibodies. After washing, phycoerythrin (PE)-conjugated streptavidin (SA10041, Thermo Fisher Scientific for phase 1 study; Cat#554061, BD Biosciences, for phase 2 study) was applied. Propidium iodide (PI, Sigma-Aldrich) or 7-AAD (BD Biosciences, Cat#51-68981) was

added to the samples to stain dead cells immediately before flow cytometry.

Intracellular staining of the H3K27me3.
PBMCs (5 ×106) were washed and incubated with Ghost DyesTM viability dye (TONBO Biosciences). Then, the cells were stained using a combination of anti-CD3-APC-Cy7, anti-CD4-Pacific Blue, anti-CD7-PE-Cy7 (clone M-T701), anti-CD14-Pacific Orange (or - BV510 for phase 2 study), anti-CADM1-biotin, and Streptavidin-PE. The surface-stained cells were then fixed and permeabilized using BD CytofixTM Fixation Buffer (BD Biosciences, 554655) and BD Phosflow Perm buffer IV (BD Biosciences, 560746) according to the manufacturer's instructions. After washing, the permeabilized cells were stained with anti-H3K27me3-Alexa Flour 488 (CST, #5499, clone C36B11), anti-Histone H3-Alexa Fluor 647 (CST, #12230, clone D1H2), anti-Rabbit IgG Isotype Control-Alexa Flour 488 (CST, #4340, clone DA1E), and anti-Rabbit IgG Isotype Control-Alexa Flour 647 (CST, #3452, clone DA1E).

H3K27me3 level was evaluated by immunoblotting with primary antibodies [anti-H3K27me3 (07-449, MERCK/Millipore), anti-histone H3 total (ab10799, Abcam), anti-FLAG M2 (F1804, Sigma)].

For ChIP-seq, validated antibodies against H3K27me3 (AM#39155, polyclonal, Active Motif), H3K27ac (AM#39133, polyclonal, Active Motif), and SUZ12 (AM#39357, polyclonal, Active Motif) were used.

For RNA immunoprecipitation (RIP) assay, Dynabeads Protein G was inculcated with anti-eIF3D (Bethyl Laboratories, A301-758A), anti-eIF3A (CST, #2013), or control IgG (CST, #2729) antibodies for 10 minutes.

Expression levels of DNMT3A and DNMT3B were evaluated by immunoblotting with primary antibodies as follows; anti-DNMT3A (#3598, Cell Signaling Technology) and anti-DNMT3B (#57868, Cell Signaling Technology).

For eIF3D knockdown, protein levels of eIF3D, PRC2 factors, and H3K27me3 were analyzed by immunoblotting with primary antibodies, as follows; anti-EZH1 (#42088, Cell Signaling Technology), anti-EZH2 (#3147, Cell Signaling Technology), anti-SUZ12 (#3737, Cell Signaling Technology), anti-EED (#85322, Cell Signaling Technology), anti-eIF3D (A301-758A, Bethyl Laboratories), anti-H3K27me3 (07-449, MERCK/ Millipore), anti-COX4 (#4850, Cell Signaling Technology), anti-TFAM (#8076, Cell Signaling Technology), anti-c-Jun (#9165, Cell Signaling Technology), and anti-b-actin (sc-69879, Santa Cruz). Alkaline phosphatase-conjugated anti-mouse (S3721, Promega) and anti-rabbit (S3731, Promega) secondary antibodies and BCIP/NBT substrate (S3771, Promega) were used for detection.

Validation

All antibodies used were validated for their use in flow-cytometery and western blotting experiments with human samples, as shown on the website provided by the respective companies.

Eukaryotic cell lines

Policy information about cell lines and Sex and Gender in Research

Cell line source(s)

ATL-derived TL-Om1 cells were kindly provided by an established researcher Dr. Sugamura. ATN-1 cells were purchased from the RIKEN BRC cell bank (RCB1440). HEK293T cells were purchased from ATCC (CRL-3216). HEK293FT cells were purchased from Thermo Fisher Scientific (R70007). DLBCL cell line WSU-DLCL2 was purchased from DSMZ (ACC 575). Normal (HTLV-1-uninfected) CD4+ T-cells were obtained from Lonza.

Authentication

These cell lines were verified by each cell bank or established researchers and monitored for cross-contamination. The HTLV-1-infected cell lines had been authenticated based on the provirus integration sites and somatic mutations by panel-based targeted sequencing. Cell surface expressions of CD4 and CADM1 were validated by flow cytometry.

Mycoplasma contamination

The cell lines were tested for mycoplasma contamination using mycoplasma detection PCR (TAKARA, #6601) and were negative for mycoplasma contamination.

Commonly misidentified lines
(See ICLAC register)

Commonly misidentified cell lines were not used in this study.

Clinical data

Policy information about clinical studies
All manuscripts should comply with the ICMJE guidelines for publication of clinical research and a completed CONSORT checklist must be included with all submissions.

Clinical trial registration

Peripheral blood samples were collected from ten patients enrolled in valemetostat phase 1 (NCT02732275) or phase 2 (NCT04102150) trials. All patients with relapsed ATL cases were categorized into clinical subtypes according to Shimoyama's criteria. This translational study has been approved by the Institutional Review Board of each hospital, research institute, and Daiichi Sankyo Co., Ltd. Written informed consents were obtained from all patients.

Study protocol

Study protocol is accessible on Clinical Trials website. More information is available from the corresponding author upon reasonable request.

Data collection

This study was conducted in parallel with phase 1 (NCT02732275) and Phase 2 trials (NCT04102150) of valemetostat. Details on the settings and places where the data were collected, as well as the periods of time for recruitment and data collection, are available at

https://clinicaltrials.gov/study/NCT02732275
https://clinicaltrials.gov/study/NCT04102150

Clinical information, including Ably and sIL-2R, was provided from the hospitals.

Outcomes

This study was conducted in parallel with Phase 1 and Phase 2 trials of valemetostat. Information of clinical outcomes were collected independently of the clinical trials.

The primary and secondary outcomes in the clinical studies were pre-defined and measured. Details of the clinical study design are available at

https://clinicaltrials.gov/study/NCT02732275
https://clinicaltrials.gov/study/NCT04102150

The clinical outcomes of the Phase 1 and 2 studies have been published in reference 6 and 7, respectively.

ChIP-seq

Data deposition

☒ Confirm that both raw and final processed data have been deposited in a public database such as GEO.

☒ Confirm that you have deposited or provided access to graph files (e.g. BED files) for the called peaks.

Data access links
May remain private before publication.

All sequencing data (fastq format), including ChIP-seq, have been deposited in the National Bioscience Database Center (NBDC) Human Database, which is associated with DNA DataBank of Japan (DDBJ) under an accession number JGAS000553.

Files in database submission

Pt1_0w_Input
Pt1_0w_H3K27me3
Pt1_4w_H3K27me3
Pt1_48w_H3K27me3
Pt1_0w_H3K27ac
Pt1_4w_H3K27ac
Pt5_Pre_Input
Pt5_Pre_H3K27me3
Pt5_CR_H3K27me3
Pt8_Pre_Input
Pt8_Pre_H3K27me3
Pt8_PR_H3K27me3
Pt8_PD_H3K27me3
normalCD4T_Input
normalCD4T_H3K27me3
normalCD4T_H3K27ac
ATN-1_parental_Input
ATN-1_parental_H3K27me3
ATN-1_resistant_Input
ATN-1_resistant_H3K27me3
ATN-1_SUZ12

Genome browser session
(e.g. UCSC)

no longer applicable

Methodology

Replicates

All ChIP-seq analyses for clinical samples has only one biological replicate due to limiting cell number.

Sequencing depth

Sample name; Total number of reads; Unique alignments without duplicate reads; Number of peaks; Length of reads; Single or Paired end
Pt1_0w_Input; 28,512,266; 22,766,989; NA; 75; SE
Pt1_0w_H3K27me3; 35,908,583; 29,122,042; 36,744; 75; SE
Pt1_4w_H3K27me3; 38,573,941; 29,464,784; 33,378; 75; SE
Pt1_48w_H3K27me3; 37,797,753; 22,885,099; 25,052; 75; SE
Pt5_Pre_Input; 49,139,727; 36,841,200; NA; 75; SE
Pt5_Pre_H3K27me3; 45,078,946; 25,086,619; 32,757; 75; SE
Pt5_CR_H3K27me3; 43,995,808; 23,175,982; 23,229; 75; SE
Pt8_Pre_Input; 43,193,123; 32,662,597; NA; 75; SE
Pt8_Pre_H3K27me3; 44,729,283; 23,761,441; 17,818; 75; SE
Pt8_PR_H3K27me3; 44,036,022; 26,133,922; 22,389; 75; SE
Pt8_PD_H3K27me3; 44,664,914; 22,558,540; 14,903; 75; SE

Pt1_0w_H3K27ac; 63,567,867; 31,085,484; 29,907; 75; SE
Pt1_4w_H3K27ac; 54,266,367; 15,815,434; 34,677; 75; SE
normalCD4T_Input; 39,012,608; 31,700,350; NA; 75; SE
normalCD4T_H3K27me3; 32,436,482; 12,477,625; 18,446; 75; SE
normalCD4T_H3K27ac; 36,341,149; 15,708,615; 31,040; 75; SE
ATN-1_parental_Input; 40,533,891; 32,783,024; NA; 75; SE
ATN-1_parental_H3K27me3; 36,323,297; 17,833,017; 30,891; 75; SE
ATN-1_resistant_Input; 49,565,134; 40,365,933; NA; 75; SE
ATN-1_resistant_H3K27me3; 37,634,603; 20,716,973; 13,335; 75; SE
ATN-1_SUZ12; 47,449,339; 35,777,362; 6,495; 75; SE

Antibodies

Processing and ChIP experiments including chromatin extraction, fragmentation, antibody-precipitation, and library preparation were performed at Active Motif (Carlsbad, CA) using validated antibodies against H3K27me3 (AM#39155, Active Motif) and H3K27ac (AM#39133, Active Motif). All antibodies used in this study and the ChIP-seq protocols performed have been validated by Active Motif.

Peak calling parameters

Reads were aligned to the human genome (hg38) using the BWA algorithm (v0.7.12). Duplicate reads were removed, and only uniquely mapped reads (mapping quality >= 25) were used for further analysis. Alignments were extended in silico at their 3'-ends to a length of 200 bp, which is the average genomic fragment length in the size-selected library, and assigned to 32-nt bins along the genome. The resulting histograms (genomic "signal maps") were stored in bigWig files.

Peak call for H3K27me3 were performed using the SICER algorithm (v1.1) with a cutoff of P value = 10-10.
SICER 1.1:
Window size: 200 bps
Fragment size: 200 bps. The shift for reads is half of 200
Effective genome size as a fraction of the reference genome of hg38: 0.86
Gap size: 600 bps
Evalue for identification of candidate islands that exhibit clustering: 1000
False discovery rate controlling significance: 1E-10

Peak call for H3K27ac were performed using the MACS algorithm (v2.1.0) with a cutoff of P value = 10-7.
MACS 2.1.0:
effective genome size = 2.70e+09
band width = 200
model fold = [5, 50]
pvalue cutoff = 1.00e-07
Broad region calling is off

Peaks that were on the ENCODE blacklist of known false ChIP-seq peaks were removed. Signal maps and peak locations were used as input data to Active Motifs proprietary analysis program, which creates Excel tables containing detailed information on sample comparison, peak metrics, peak locations and gene annotations. EaSeq software (v1.111) was also used to calculate each peak value and create heatmaps.

Data quality

ChIP-seq quality was assessed in three ways: i. number of peaks called relative to appropriate input sample; ii. number of reads in the significantly called peaks over reads in the background; iii. cross correlation plots.
Please see sequencing depth information above for the peak counts for all experiments.

Software

BWA algorithm (v0.7.12)
MACS algorithm (v2.1.0)
SICER algorithm (v1.1)
EaSeq software (v1.111)

Flow Cytometry

Plots

Confirm that:

☒ The axis labels state the marker and fluorochrome used (e.g. CD4-FITC).

☒ The axis scales are clearly visible. Include numbers along axes only for bottom left plot of group (a 'group' is an analysis of identical markers).

☒ All plots are contour plots with outliers or pseudocolor plots.

☒ A numerical value for number of cells or percentage (with statistics) is provided.

Methodology

Sample preparation

ATL cell populations were obtained using a HAS-flow method, as described previously19. Single-cell suspensions of lymphocytes were stained with fluorescent-labeled antibodies. An unlabeled CADM1 antibody (CM004-6, clone 3E1) and an isotype control chicken immunoglobulin Y (IgY) antibody (2:100) were purchased from MBL. These were biotinylated (primary amine biotinylation) using biotin N-hydroxysuccinimide ester (Sigma-Aldrich). Anti-CD14-Pacific Orange antibody

(MHCD1430, clone TuK4) was purchased from Invitrogen. All other antibodies were obtained from BioLegend. Cells were stained using a combination of anti-CADM1-biotin (MBL, CM004-6, 1:100), anti-CD7-APC (clone CD7-6B7, 5:100), anti-CD3-APC-Cy7 (clone SK7, 5:100), anti-CD4-Pacific Blue (clone RPA-T4, 5:100), and anti-CD14-Pacific Orange (5:100) antibodies. After washing, phycoerythrin (PE)-conjugated streptavidin (SA10041, 2:100, Thermo Fisher Scientific for phase 1 study; Cat#554061, 1:80, BD Biosciences, for phase 2 study) was applied. Propidium iodide (PI, Sigma-Aldrich) or 7-AAD (BD Biosciences, Cat#51-68981) was added to the samples to stain dead cells immediately before flow cytometry.

For intracellular staining of the H3K27me3, we improved the HAS-Flow method. First, PBMCs (5 ×106) were washed and incubated with Ghost DyesTM viability dye (TONBO Biosciences). Then, the cells were stained using a combination of anti-CD3-APC-Cy7, anti-CD4-Pacific Blue, anti-CD7-PE-Cy7 (clone M-T701, 5:100), anti-CD14-Pacific Orange (or -BV510 for phase 2 study), anti-CADM1-biotin, and Streptavidin-PE. The surface-stained cells were then fixed and permeabilized using BD CytofixTM Fixation Buffer (BD Biosciences, 554655) and BD Phosflow Perm buffer IV (BD Biosciences, 560746) according to the manufacturer's instructions. After washing, the permeabilized cells were stained with anti-H3K27me3-Alexa Flour 488 (CST, clone C36B11, 1:50), anti-Histone H3-Alexa Fluor 647 (CST, clone D1H2, 1:100), anti-Rabbit IgG Isotype Control-Alexa Flour 488 (CST, clone DA1E, 1:100), and anti-Rabbit IgG Isotype Control-Alexa Flour 647 (CST, clone DA1E, 1:100).

Instrument

FACSAria II or FACSLyric instrument (BD Biosciences) was used for multicolor flow cytometry and fluorescence-activated cell sorting.
Expression of fluorescent proteins and tumor cell markers (CD4+CADM1+CD7-) were confirmed by flow-cytometery using FACSCalibur or FACSymphony A1 (BD Biosciences), or by automated cell counter using Countess 3 FL (Thermo Fisher Scientific).

Software

The collected data were analyzed by FlowJo (v10.7.1) software (Tree Star).

Cell population abundance

After cell sorting, we were able to assess purity in our cell sorts by assessing HTLV-1 provirus PCR. In addition, expression pattern of cell surface markers such as CADM1 and CD7 were validated using RNA-seq data.

Gating strategy

Gating was determined using fluorescent-minus-one controls for each color used in each FACS experiment to ensure that positive populations were solely associated with the antibody for that specific marker.
CD4+/CADM1+/CD7− cells and CD4+/CADM1−/CD7+ cells were analyzed as malignant ATL cells and non-malignant cells, respectively. Tumor H3K27me3 levels (mean fluorescence intensity, MFI) were calculated by normalization with the data of normal CD4+ T-cells.

☒ Tick this box to confirm that a figure exemplifying the gating strategy is provided in the Supplementary Information.
